# Final analysis of the international observational S-Collate study of peginterferon alfa-2a in patients with chronic hepatitis B

**DOI:** 10.1371/journal.pone.0230893

**Published:** 2020-04-10

**Authors:** Patrick Marcellin, Qing Xie, Seung Woon Paik, Robert Flisiak, Teerha Piratvisuth, Jörg Petersen, Tarik Asselah, Markus Cornberg, Denis Ouzan, Graham R. Foster, Georgios Papatheodoridis, Diethelm Messinger, Loredana Regep, Georgios Bakalos, Ulrich Alshuth, Pietro Lampertico, Heiner Wedemeyer

**Affiliations:** 1 Hepatologie, Université Paris Diderot, Clichy, France; 2 Infectious Diseases, Ruijin Hospital, Shanghai, China; 3 Gastroenterology and Hepatology, Sungkyunkwan University School of Medicine, Seoul, South Korea; 4 Infectious Disease and Hepatology, Medical University of Białystok, Białystok, Poland; 5 Gastroenterology and Hepatology, Prince of Songkla University, Songkhla, Thailand; 6 Liver Unit, Asklepios Klinik St. Georg, Hamburg, Germany; 7 Gastroenterology, Hepatology and Endocrinology, Hannover Medical School, Hannover, Germany; 8 Service d’Hepatologie, Institut Arnault Tzanck, Saint-Laurent-du-Var, France; 9 Liver Unit, Queen Mary University of London, London, United Kingdom; 10 Gastroenterology, Laiko General Hospital, Athens, Greece; 11 PROMETRIS GmbH, Mannheim, Germany; 12 Roche s.r.o., Prague, Czech Republic; 13 F. Hoffmann-La Roche Ltd, Basel, Switzerland; 14 Roche Pharma AG, Grenzach-Wyhlen, Germany; 15 Gastroenterology and Hepatology, University of Milan, Milan, Italy; Yonsei University College of Medicine, REPUBLIC OF KOREA

## Abstract

**Background and aims:**

Sustained off-treatment immune control is achievable in a proportion of patients with chronic hepatitis B treated with peginterferon alfa-2a. We evaluated on-treatment predictors of hepatitis B surface antigen (HBsAg) clearance 3 years after peginterferon alfa-2a treatment and determined the incidence of hepatocellular carcinoma.

**Methods:**

A prospective, international, multicenter, observational study in patients with chronic hepatitis B who have been prescribed peginterferon alfa-2a (40KD) in a real-world setting. The primary endpoint was HBsAg clearance after 3 years’ follow-up.

**Results:**

The modified intention-to-treat population comprised 844 hepatitis B e antigen (HBeAg)-positive patients (540 [64%] completed 3 years’ follow-up), and 872 HBeAg-negative patients (614 [70%] completed 3 years’ follow-up). At 3 years’ follow-up, HBsAg clearance rates in HBeAg-positive and HBeAg-negative populations, respectively, were 2% (16/844) and 5% (41/872) in the modified intention-to-treat population and 5% [16/328] and 10% [41/394] in those with available data. In HBeAg-positive patients with data, Week 12 HBsAg levels <1500, 1500–20,000, and >20,000 IU/mL were associated with HBsAg clearance rates at 3 years’ follow-up of 11%, 1%, and 5%, respectively (Week 24 predictability was similar). In HBeAg-negative patients with available data, a ≥10% decline vs a <10% decline in HBsAg at Week 12 was associated with HBsAg clearance rates of 16% vs 4%. Hepatocellular carcinoma incidence was lower than REACH-B (Risk Estimation for Hepatocellular Carcinoma in Chronic Hepatitis B) model predictions.

**Conclusions:**

Sustained off-treatment immune control is achieved with peginterferon alfa-2a in a real-world setting. HBsAg clearance 3 years after completion of peginterferon alfa-2a can be predicted on the basis of on-treatment HBsAg kinetics.

## Introduction

Patients with chronic hepatitis B (CHB) are at increased risk of cirrhosis, hepatocellular carcinoma (HCC), and liver-related death [1], and CHB is estimated to be responsible for 0.5–1 million deaths each year [2].

The goal of therapy is to prevent disease progression, and the ideal endpoint is sustained off-treatment hepatitis B surface antigen (HBsAg) loss [2]. Current treatment options include nucleos(t)ide analogs (NAs) and interferons. NAs suppress hepatitis B virus (HBV) DNA replication and are well tolerated, but relapse occurs frequently after withdrawal of therapy and HBsAg loss is rare [3]; therefore, NA therapy must be continued for life to remain effective in the majority of patients [2, 4].

Durable off-treatment responses are more likely after a finite course of treatment with peginterferon alfa-2a. Approximately one-third of patients had sustained responses 24 weeks after the end of treatment (EOT) with peginterferon alfa-2a in phase 3 studies [5, 6]. HBsAg levels decreased in many patients during treatment and the extent of reduction in HBsAg was associated with durable serologic response [7–9].

Available data suggest that HBsAg clearance is durable after treatment with peginterferon alfa-2a in some patients [10–12]. The real-world S-Collate study assessed, in routine clinical practice, on-treatment predictors of HBsAg clearance 3 years after cessation of treatment with peginterferon alfa-2a.

## Materials and methods

S-Collate was a prospective, international, multicenter, observational study in patients with CHB prescribed peginterferon alfa-2a (40KD) according to the standard of care and label in each country. Patients co-infected with hepatitis A or C or HIV were ineligible. Peginterferon alfa-2a dosing and treatment duration were at the physician’s discretion. First patient enrollment was April 11, 2009, with last follow-up November 17, 2014. Due to an administrative delay, the study was registered with clinicaltrials.gov (NCT01011738) in November 2009, after the first patient had been recruited. The authors confirm that all ongoing and related trials for this drug/intervention are registered.

### Assessments and follow-up

Assessments as part of routine care in accordance with current guidelines and the local standard of care were documented in an electronic case report form during and for up to 3 years after EOT. Patients withdrawn from treatment remained under observation with data collection, if possible, even after receiving other therapy(ies) for CHB.

The primary efficacy endpoint was HBsAg clearance (proportion of patients HBsAg-negative 3 years post-treatment). Secondary efficacy endpoints included the proportion of patients with HBsAg <1000, <100, and <10 IU/mL, HBsAg seroconversion, and HBV DNA <2000 IU/mL and alanine aminotransferase (ALT) normalization (ALT at or below upper limit of normal for the local laboratory) over time (0.5, 1, 2, and 3 years post-treatment). Secondary endpoints in hepatitis B e antigen (HBeAg)-positive patients were HBeAg loss and seroconversion, and the combined endpoint of HBeAg seroconversion and HBV DNA <2000 IU/mL over time. The combined endpoint of HBV DNA <2000 IU/mL and normal ALT over time was a secondary endpoint in HBeAg-negative patients. All laboratory assays were conducted locally.

The incidence of HCC, diagnosed according to standard practice in each country, was a secondary endpoint. To compare incidence with that expected in this population, expected incidence was calculated according to the risk function of the REACH-B (Risk Estimation for Hepatocellular Carcinoma in Chronic Hepatitis B) model, which uses baseline clinical characteristics (gender, age, ALT level, HBeAg status, and HBV DNA level) to predict risk at 3, 5, and 10 years [13]. Assuming a Weibull distribution for time to HCC, the risk was calculated for up to 4 years in all patients for whom sufficient baseline data were available, and was extrapolated to the overall population.

Safety was assessed using adverse event (AE) data and by other clinical endpoints associated with CHB.

The protocol and patient materials were approved by independent ethics committees at each center (S1 Appendix) before recruitment of any patients. The study was conducted in accordance with the principles of the Declaration of Helsinki and Good Pharmacoepidemiology Practices [14]. All patients provided written informed consent.

### Statistical considerations

Assuming a HBsAg clearance rate of 9–12% at 3 years post-treatment [15, 16], planned enrollment was 1600–2000 patients (600–800 HBeAg-negative and 1000–1200 HBeAg-positive) and the 95% confidence interval (CI) for HBsAg clearance was expected to be ±1.25% to ±1.56%.

The modified intention-to-treat (mITT) population comprised adults classified as HBeAg-positive or HBeAg-negative who received at least one dose of peginterferon alfa-2a. Data were analyzed using two methods: firstly, patients with missing variables for the endpoint in question were included in the analysis and considered non-responders; secondly, analysis was restricted to patients with available data for the endpoint in question (mITT with available data). The safety population included all patients who received ≥1 dose of peginterferon alfa-2a with ≥1 post-baseline safety assessment.

Data in HBeAg-positive and HBeAg-negative patients were analyzed separately using SAS software version 9.4. Associations between baseline and on-treatment factors and HBsAg clearance were explored by univariate and multiple logistic regression (MLR). Baseline factors included age, sex, body weight, body mass index, race (Chinese [China or Hong Kong] vs non-Chinese), main treatment regimens, ALT ratio, HBsAg, and HBV DNA levels. On-treatment factors (at Weeks 12 and 24) included HBsAg, drop in HBsAg (log_10_), HBV DNA, and drop in HBV DNA (log_10_). For selection of the factors to be included in the MLR the Least Absolute Shrinkage and Selection Operator (LASSO) method was applied considering the optimal models to be those with the lowest Schwarz Bayesian criterion (SBC). The area under the receiver operating characteristic curve (ROC AUC) was determined for the final logistic regression models.

A *post hoc* analysis investigated the impact of missing values on HBsAg clearance. Generalized estimating equations (GEE) methods for longitudinal data with missing values were used to estimate the HBsAg clearance rate at EOT and 0.5, 1, 2, and 3 years post-treatment in the mITT population. For the repeated measurements an autoregressive working correlation structure was assumed.

In addition, the fully conditional method was applied to multiple imputation for non-monotone multivariate missing data [17, 18]. As well as HBsAg clearance, the following covariates were considered: age, body mass index, alcohol consumption (yes/no), region (China/Hong Kong vs others), duration of treatment (≥48, ≥24 to <48, and <24 weeks) and baseline ALT ratio, all of which were predictive for missing HBsAg values at 3 years post-treatment. Logistic regression was used to impute missing values for longitudinal/repeated measures of HBsAg clearance (yes/no) at EOT and 0.5, 1, 2, and 3 years post-treatment, a regression method was used for missing continuous variables, and a discrimination function method was used for categorical data. Multiple imputations were repeated 50 times to estimate HBsAg clearance rates.

## Results

First patient enrollment was April 11, 2009, with last follow-up November 17, 2014. Overall, 1842 patients (877 HBeAg-positive, 912 HBeAg-negative, 53 unknown HBeAg status) were enrolled from 219 centers in 26 countries [S2 Appendix]).

**Fig 1:**
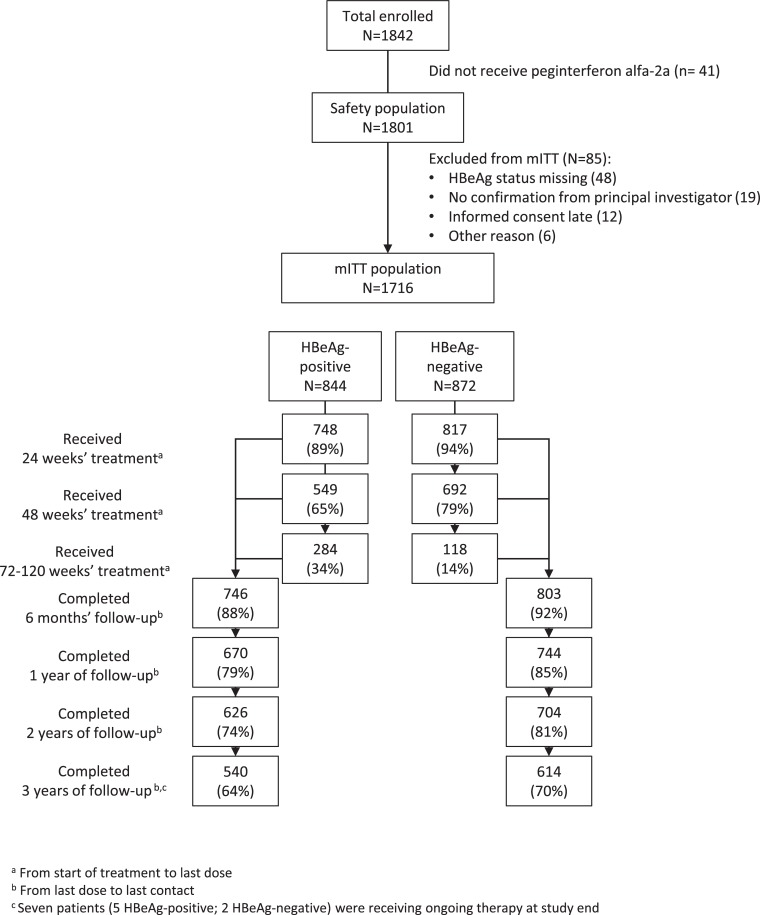
Patient disposition. HBeAg, hepatitis B e antigen; mITT, modified intention-to-treat. ^a^From start of treatment to last dose. ^b^From last dose to last contact. ^c^Seven patients (five HBeAg-positive; two HBeAg-negative) were receiving ongoing therapy at study end.

The mITT population comprised 844 HBeAg-positive (540 [64%] completed 3 years’ follow-up), and 872 HBeAg-negative patients (614 [70%] completed 3 years’ follow-up) (Fig 1). Baseline characteristics are shown in Table 1.

**Table 1:** Baseline disease and demographic characteristics in the mITT population.

Characteristic	HBeAg-positive n = 844	HBeAg-negative n = 872
Male sex, n (%)	592 (70)	644 (74)
Age, years	31.2 (9.3)	38.0 (10.8)
Race, n/N (%)		
Asian	640/796 (80)	285/731 (39)
White	138/796 (17)	419/731 (57)
Black	11/796 (1)	25/731 (3)
Other	7/796 (<1)	2/731 (<1)
BMI, kg/m^2^	23.3 (3.7), n = 829	25.0 (4.0), n = 860
Previous interferon therapy, n (%)	71 (8)	75 (9)
Previous nucleos(t)ide therapy, n (%)	156 (18)	131 (15)
Hepatitis D virus co-infection, n/N (%)	8/369 (2)	28/485 (6)
HBV DNA, log_10_ IU/mL	6.25 (1.70), n = 732	4.53 (1.72), n = 757
HBsAg, log_10_ IU/mL	3.94 (0.77), n = 354	3.45 (0.85), n = 370
ALT, IU/L	179 (152), n = 834	118 (111), n = 846
ALT ratio x ULN	3.3 (2.8), n = 834	2.1 (2.0), n = 846
Genotype, n/N (%)		
A	22/242 (9)	35/220 (16)
B	43/242 (18)	23/220 (10)
C	94/242 (39)	17/220 (8)
D	67/242 (28)	116/220 (53)
Other	16/242 (7)	29/220 (13)
Method to assess liver fibrosis, n (%)		
Biopsy (invasive)	193 (23)	349 (40)
Non-invasive method	163 (19)	183 (21)
Not assessed	488 (58)	340 (39)
Results of liver fibrosis assessment, n/N (%)		
No cirrhosis	305/356 (86)	455/532 (86)
Transition to cirrhosis	37/356 (10)	50/532 (9)
Cirrhosis	14/356 (4)	27/532 (5)
FibroScan value in kPa	9.7 (6.9) n = 56	7.6 (3.5) n = 95

ALT, alanine aminotransferase; ALT ratio, individual patient’s ALT divided by the ULN for the local laboratory; BMI, body mass index; HBsAg, hepatitis B surface antigen; HBV, hepatitis B virus; ULN, upper limit of normal.

Mean (standard deviation) unless otherwise stated.

Peginterferon alfa-2a monotherapy was the most common regimen, administered to 697/844 (83%) HBeAg-positive and 753/872 (86%) HBeAg-negative patients (S1 Table). Treatment duration was 48 weeks in 379/844 (45%) HBeAg-positive and 628/872 (73%) HBeAg-negative patients.

Among HBeAg-positive patients, 697 (83%) received peginterferon alfa-2a monotherapy, 28 (3%) received combination peginterferon alfa-2a plus NA therapy, 30 (4%) received peginterferon alfa-2a add-on therapy, and 89 (11%) received another regimen, while 549 (65%) received treatment for at least 48 weeks, with 149 (18%) treated for 72 weeks or longer. Among HBeAg-negative patients, 753 (86%) received peginterferon alfa-2a monotherapy, 31 (4%) received combination peginterferon alfa-2a plus NA therapy, 47 (5%) received peginterferon alfa-2a add-on therapy, and 41 (5%) received another regimen, while 692 (79%) received treatment for at least 48 weeks, with 70 (8%) treated for 72 weeks or longer (S1 Table).

### Efficacy in HBeAg-positive patients

**Fig 2:**
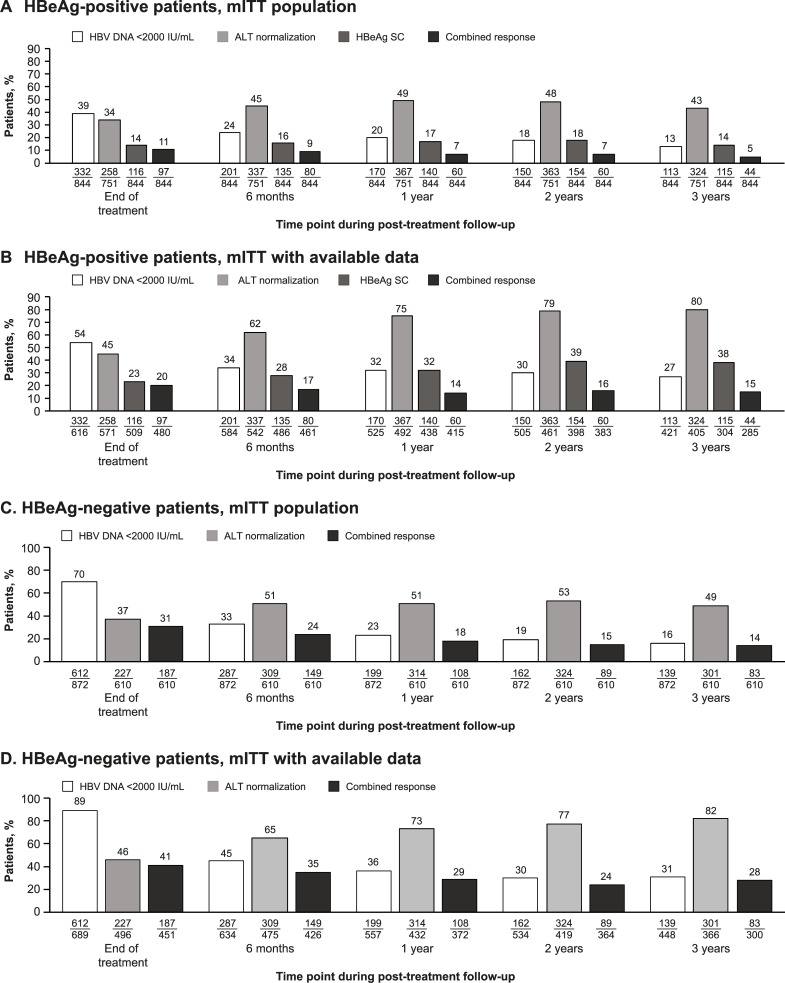
Response rates in HBeAg-positive patients (HBV DNA <2000 IU/mL, ALT normalization, HBeAg seroconversion, and combined responses) and HBeAg-negative patients (HBV DNA <2000 IU/mL, ALT normalization, and combined responses) over time (mITT population and mITT patients with available data). Patients receiving nucleos(t)ide analogs were considered to be non-responders for HBV DNA endpoints at the respective time points. ALT response includes only patients with elevated ALT at baseline. ALT, alanine aminotransferase; HBeAg, hepatitis B e antigen; HBV, hepatitis B virus; mITT, modified intention-to-treat; SC, seroconversion.

The proportion of patients with a virologic response (HBV DNA <2000 IU/mL), HBeAg seroconversion, and a combined response is shown in Fig 2. In the mITT population, 11% had a combined response at EOT and 5% after 3 years’ follow-up (Fig 2A). Excluding patients with missing measurements, these rates were 20% at EOT and 15% after 3 years’ follow-up (Fig 2B).

HBsAg <1500 IU/mL at Weeks 12 or 24 of treatment was associated with higher rates of HBeAg seroconversion and combined response rates (Table 2).

**Table 2:** Response rates after 3 years’ follow-up according to HBsAg level at Weeks 12 and 24 of treatment in HBeAg-positive patients. mITT patients with available data.

HBsAg level, IU/mL	Response 3 years post-treatment, n/N (%)		
	HBeAg seroconversion	Combined response (HBeAg seroconversion and HBV DNA <2000 IU/mL)	HBsAg clearance
Week 12			
<1500	18/33 (55)	12/31 (39)	4/35 (11)
1500–20,000	28/82 (34)	9/76 (12)	1/94 (1)
>20,000	13/39 (33)	3/38 (8)	2/42 (5)
*P*-value^a^	0.0764	0.0010	0.1877
Week 24			
<1500	24/44 (55)	16/40 (40)	6/55 (11)
1500–20,000	30/74 (41)	10/69 (14)	1/77 (1)
>20,000	8/34 (24)	2/33 (6)	1/35 (3)
*P*-value^a^	0.0058	0.0002	0.0432

HBeAg, hepatitis B e antigen; HBsAg, hepatitis B surface antigen; HBV, hepatitis B virus; mITT, modified intention-to-treat.

Patients with missing HBsAg values at Weeks 12 or 24 are not shown.

^a^Two-sided Cochran–Armitage trend test.

**Fig 3:**
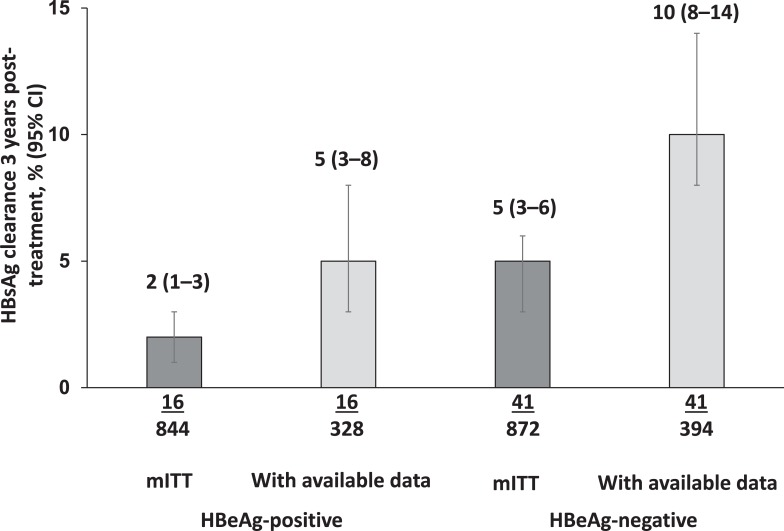
HBsAg clearance after 3 years’ follow-up in the mITT population and mITT patients with available data. CI, confidence interval; HBeAg, hepatitis B e antigen; HBsAg, hepatitis B surface antigen; mITT, modified intention-to-treat.

The HBsAg clearance rate 3 years post-treatment was 2% (16/844) (95% CI 1–3) in the mITT population, and 5% (16/328) (95% CI 3–8) in patients with available data (Fig 3). HBsAg clearance rates in patient subgroups are shown in S2 Table. Of 16 HBeAg-positive patients with HBsAg clearance, 14 had received peginterferon alfa-2a monotherapy.

HBsAg clearance was higher in patients with HBsAg <1500 IU/mL at Weeks 12 (11%) or 24 of treatment (11%) than in patients with higher HBsAg levels at these time points (Table 2).

Among pre-treatment and on-treatment factors analyzed for prediction of HBsAg clearance at 3 years post-treatment (mITT population) using LASSO selection followed by MLR model inspection, only log_10_ HBsAg at Week 24 remained as significant predictor (odds ratio [OR] 0.386, 95% CI 0.236–632, *P* = 0.0001; ROC AUC = 0.802). Addition of log_10_ drop in HBV DNA from baseline to Week 12 or 24 improved the prediction model slightly, but neither variable reached statistical significance in the MLR.

**Fig 4:**
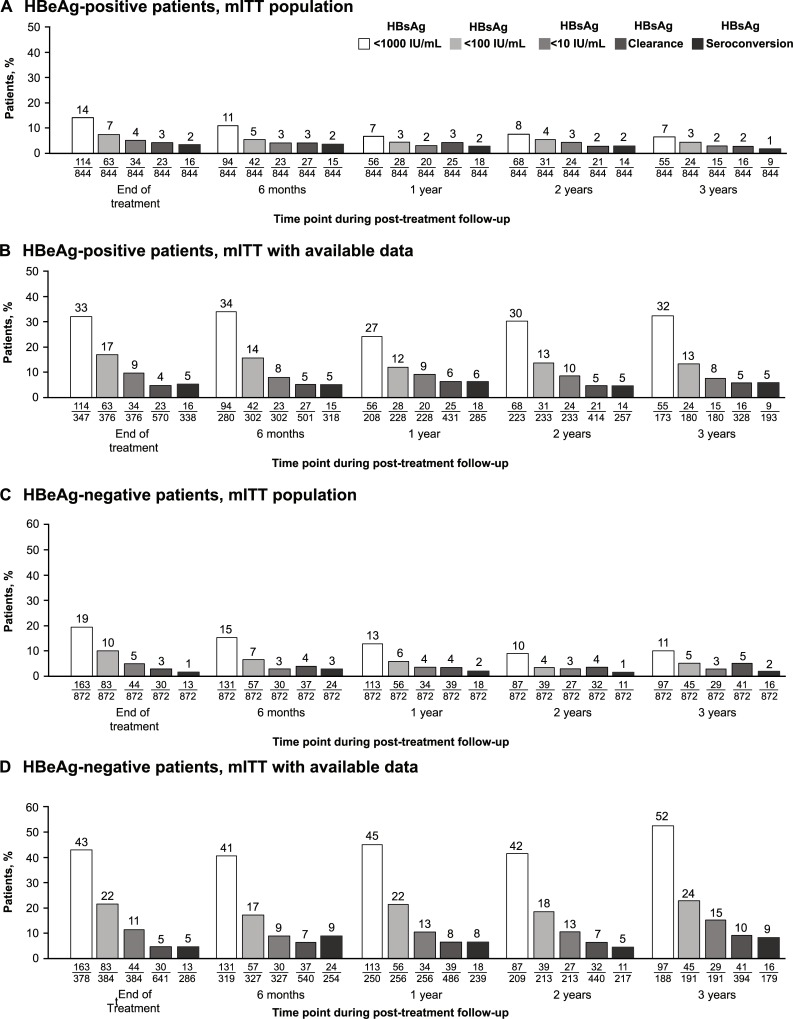
HBsAg levels, HBsAg loss, and HBsAg seroconversion over time in the mITT population and mITT patients with available data. HBeAg, hepatitis B e antigen; HBsAg, hepatitis B surface antigen; mITT, modified intention-to-treat.

The proportion of patients with HBsAg <1000, <100, and <10 IU/mL decreased over time in the mITT population (Fig 4A); however, considering only mITT patients with available data, the proportions remained relatively stable over time, and at the end of 3 years’ follow-up, approximately one-third of HBeAg-positive patients (32%) had HBsAg <1000 IU/mL and 8% had HBsAg <10 IU/mL (Fig 4B). A total of 5% of HBeAg-positive patients with available data had experienced HBsAg seroconversion after 3 years’ follow-up.

Using the GEE methods for longitudinal repeated measurements with missing values, the HBsAg clearance rates were 6% (95% CI 4–8) at 1 year, 6% (95% CI 4–7) at 2 years, and 6% (95% CI 4–8) at 3 years post-treatment. If multiple imputations for missing data were applied the estimated rates were almost identical: 6% (95% CI 4–9) at 1 year, 6% (95% CI 3–8) at 2 years, and 7% (95% CI 4–10) at 3 years (S3 Table).

### Efficacy in HBeAg-negative patients

In the mITT population, 31% had a combined response at EOT and 14% after 3 years’ follow-up (Fig 2C). When patients with missing measurements were excluded, these rates were 41% at EOT and 28% after 3 years’ follow-up (Fig 2D).

The HBsAg clearance rate 3 years post-treatment was 5% (41/872) (95% CI 3–6) in the mITT population, and 10% (41/394) (95% CI 8–14) in patients with available data (Fig 3). Among HBeAg-negative patients, the rate of HBsAg clearance was 4% (31/753) in those who received peginterferon alfa-2a monotherapy, and 11% (5/47) among those who received peginterferon added-on to NA therapy (S2 Table).

For patients with available data, a decline in HBsAg of ≥10% between baseline and Weeks 12 or 24 of treatment was associated with HBsAg clearance rates of 16% and 17%, respectively, after 3 years’ follow-up (Table 3). In contrast, post-treatment HBsAg clearance rates in patients with <10% decline in HBsAg between Weeks 12 and 24 of treatment were 4% and 2%, respectively (Table 3).

**Table 3:** HBsAg clearance rates after 3 years’ follow-up according to the decline in HBsAg level between baseline and Weeks 12 and 24 of treatment in HBeAg-negative patients. mITT patients with available data.

Decline in HBsAg level from baseline	HBsAg clearance 3 years post-treatment, n/N (%)	*P*-value^a^
Week 12		
Any	12/89 (13)	0.0562
None	3/72 (4)	
≥10%	12/77 (16)	0.0128
<10%	3/84 (4)	
Week 24		
Any	14/94 (15)	0.0109
None	1/53 (2)	
≥10%	14/82 (17)	0.0018
<10%	1/65 (2)	

HBeAg, hepatitis B e antigen; HBsAg, hepatitis B surface antigen; mITT, modified intention to treat

^a^Two-sided Fisher exact test.

Among on-treatment factors analyzed for prediction of HBsAg clearance at 3 years post-treatment (mITT population) using LASSO selection followed by MLR model inspection, only a lower HBsAg value at Week 24 remained as significant predictor (odds ratio [OR] 0.250, 95% CI 0.158–0.395, *P* = <0.0001; ROC AUC = 0.920). The odds of HBsAg clearance increased fourfold (= 1/0.250) for each log_10_ decrease in HBsAg at Week 24.

When both pre-treatment and on-treatment predictors were considered, in addition to a lower HBsAg at Week 24 (OR 0.247, 95% CI 0.149–0.408, *P* = <0.0001), only a higher baseline ALT (OR 1.213, 95% CI 0.977–1.505, P = 0.0806) was identified as additional weak predictor, but this did not reach statistical significance.

The proportion of patients with HBsAg <1000, <100, and <10 IU/mL decreased over time in the mITT population (Fig 4C); however, when only patients with available data were included, the proportions remained relatively stable over time and after 3 years’ follow-up approximately half of HBeAg-negative patients (52%) had HBsAg <1000 IU/mL and 15% had HBsAg <10 IU/mL (Fig 4D). A total of 9% of HBeAg-negative patients with available data had experienced HBsAg seroconversion after 3 years’ follow-up.

Using the GEE methods for longitudinal repeated measurements with missing values, the HBsAg clearance rates were 8% (95% CI 6–10) at 1 year, 9% (95% CI 7–11) at 2 years, and 11% (95% CI 8–14) at 3 years post-treatment. If multiple imputations for missing data were applied the estimated rates were almost identical: 9% (95% CI 6–11) at 1 year, 8% (95% CI 5–11) at 2 years, and 11% (95% CI 8–14) at 3 years (S3 Table).

### Adverse events

Among the 1801 patients in the safety population, 103 (6%) withdrew due to AEs and 1294 (72%) and 112 (6%) experienced at least one AE and serious AE (SAE), respectively (Table 4). The most common AEs were thrombocytopenia (14%), pyrexia (13%), and neutropenia (13%) (Table 4), consistent with the known tolerability of peginterferon alfa-2a. Depression was reported in 69 of 1801 (4%) patients.

**Table 4:** AEs in the safety analysis set.

Patients, n (%)	HBeAg-positive n = 863	HBeAg-negative n = 890	Total^a^ N = 1801
Any AE	618 (72)	646 (73)	1294 (72)
Any SAE (regardless of relationship to treatment)	47 (5)	63 (7)	112 (6)
Any treatment-related SAE	18 (2)	22 (2)	40 (2)
AE leading to withdrawal	50 (6)	48 (5)	103 (6)
Death	3 (<1)	9 (1)	12 (<1)
Incidence of individual AEs^b^			
Thrombocytopenia	54 (6)	185 (21)	248 (14)
Pyrexia	147 (17)	92 (10)	241 (13)
Neutropenia	76 (9)	153 (17)	234 (13)
Headache	84 (10)	121 (14)	212 (12)
Asthenia	64 (7)	124 (14)	195 (11)
Leukopenia	56 (6)	124 (14)	186 (10)
Fatigue	89 (10)	93 (10)	185 (10)
Alopecia	113 (13)	67 (8)	181 (10)
ALT flares on treatment			
>5−10 x ULN	73 (9)	76 (9)	154 (9)
>10 x ULN	26 (3)	22 (3)	49 (3)

AE, adverse event; ALT, alanine aminotransferase; HBeAg, hepatitis B e antigen; SAE, serious AE; ULN, upper limit of normal.

^a^Includes 48 patients with unknown HBeAg status.

^b^AEs with an incidence ≥10%.

A total of 40 patients experienced SAEs remotely, possibly or probably related to treatment, including 10 with hematologic events (five neutropenia, three thrombocytopenia, two anemia, and one leukopenia), eight with hepatobiliary events, and four with psychiatric events (two depression and two major depression).

ALT flares >5–10 x the upper limit of normal (ULN) and >10 x ULN were reported in 154 (9%) and 49 (3%) patients, respectively.

Twelve cases of HCC were detected compared with 19 expected in this population using the REACH-B function. After 1 year of follow-up there were three cases of HCC (three expected), after 2 years there were six cases (eight expected), and after 3 years there were nine cases (14 expected). In the 970 Asian/Oriental patients there were three cases of HCC at 4 years compared with ten predicted by REACH-B. The REACH-B score was developed in patients without cirrhosis and in 794 patients without cirrhosis/transition to cirrhosis there were five cases (nine predicted). Conclusions could not be drawn due to small numbers. One patient with HCC underwent liver transplantation.

Two patients died during treatment and 10 during follow-up (Table 4). Eleven deaths were unrelated to study treatment; one was considered remotely related. Four deaths were due to HCC (unrelated), one to pulmonary metastases in a patient with HCC (remotely related), and one to hepatic coma and septic shock (unrelated). Non-hepatic causes of death included road traffic accidents (two), cardiopulmonary arrest, ruptured cerebral aneurysm, malignant melanoma, and gastric cancer.

## Discussion

This prospective, international, multicenter, observational study shows that HBsAg clearance is durable in a proportion of HBeAg-positive and HBeAg-negative patients for up to 3 years after completion of peginterferon alfa-2a therapy. In the mITT population, the rate of HBsAg clearance after 3 years of follow-up was 2% (16/844) in HBeAg-positive patients and 5% (41/872) in HBeAg-negative patients. However, when the large number of patients without information on HBsAg clearance at 3 years’ follow-up (61% and 56%, respectively) are excluded, the final HBsAg clearance rates were 5% (16/328) and 10% (41/394), respectively. Moreover, HBsAg clearance rates increased over 3 years in both cohorts when multiple imputation methods were applied, and predictive covariates used to estimate HBsAg for patients with missing values. These results indicate that the response rates in patients with available data do not overestimate the real response rates. It is plausible that missing HBsAg data were more frequent at follow-up visits where HBsAg clearance was documented at the previous visit than after visits where HBsAg remained detectable.

S-Collate included a large, diverse cohort of patients with active disease treated under conditions reflecting everyday clinical practice. Data are available for 1842 HBeAg-positive and HBeAg-negative patients infected with all major HBV genotypes from 26 countries in Africa, Asia, Europe, the Middle East, and the Pacific region. Approximately one-quarter of the patients had received prior treatment for CHB and many received NA in parallel or subsequent to treatment with peginterferon alfa-2a.

Spontaneous HBsAg clearance and seroconversion do not occur at constant rates in untreated cohorts, but typically occur at low rates in ‘inactive carriers’ who have persistently low or undetectable HBV DNA levels [19–21], and generally occur more commonly in HBeAg-negative than HBeAg-positive patients [22, 23]. Thus, the observed HBsAg clearance rates exceed what would be expected in this population and suggest that treatment with peginterferon alfa-2a modifies the natural history of the disease in a manner that is maintained for several years after stopping therapy.

HBsAg clearance rates increased over time when HBsAg clearance was estimated using the multiple imputation approach. In large randomized studies, the rate of HBsAg seroconversion 6 months post-treatment was 2–7% in HBeAg-positive [5, 24, 25] and 2% in HBeAg-negative [6] patients. HBsAg clearance after 3 years of untreated follow-up in S-Collate was somewhat lower in HBeAg-positive than in HBeAg-negative patients and lower than seen in HBeAg-negative patients after 3–5 years in small follow-up studies [16, 26].

Most patients did not experience HBsAg clearance in S-Collate but suppression of HBsAg below 1000, 100, and 10 IU/mL for up to 3 years post-treatment occurred in a clinically significant proportion. In the mITT population, 7% and 11% of HBeAg-positive and HBeAg-negative patients, respectively, had HBsAg <1000 IU/mL at 3 years’ follow-up; of patients with available data, approximately one-third and one-half, respectively, of HBeAg-positive and HBeAg-negative patients had HBsAg <1000 IU/mL at 3 years’ follow-up.

A substantial proportion of patients had combined responses at EOT and during follow-up. A total of 41% of HBeAg-negative patients with available data had normal ALT and HBV DNA <2000 IU/mL at EOT, and the response rates at 1 and 3 years post-treatment were 29% and 28%, respectively. Among HBeAg-positive patients with available data, 20% achieved HBeAg seroconversion and HBV DNA <2000 IU/mL at EOT, and a response rate of 14–15% was observed at 1 and 3 years post-treatment. Among patients with elevated baseline ALT, 45% had normal ALT at EOT and 80% of patients with available data had normal ALT after 3 years’ follow-up.

These results support and extend the findings of a retrospective analysis of data from HBeAg-positive patients in a phase 3 study of peginterferon alfa-2a [27]. Patients with HBsAg <1500 IU/mL at Weeks 12 and 24 of treatment had high HBsAg clearance rates (11–12%) 6 months post-treatment [27]. In the present study, HBsAg <1500 IU/mL at Weeks 12 or 24 of treatment was associated with HBsAg clearance 3 years post-treatment. HBsAg <1500 IU/mL at Week 12 has been associated with sustained immune control (HBeAg loss and HBV DNA <2000 IU/mL) in HBeAg-positive patients [28], while HBsAg ≥20,000 IU/mL at Week 24 has been associated with non-response (negative predictive value 92–98%) [28]. In the present study, HBsAg levels <1500, 1500–20,000, or >20,000 IU/mL at Week 12 were associated with combined response rates of 39%, 12%, and 8%, respectively, 3 years post-treatment in patients with available data.

In HBeAg-negative patients with available data, a ≥10% decline in HBsAg at Weeks 12 or 24 was associated with higher HBsAg clearance rates (16% and 17%, respectively) at 3 years’ follow-up compared with patients with <10% decline (4% and 2%, respectively). A previous long-term follow-up study [26] showed HBsAg clearance rates of 22–23% at 5 years post-treatment in patients with a ≥10% decline in HBsAg at Weeks 12 and 24 of treatment, while in patients without a 10% decline HBsAg clearance was 4–8%.

The safety of peginterferon alfa-2a was consistent with that reported in large randomized studies [5, 6, 24]. The incidence of SAEs (6%) was similar to that seen in trials using the approved dosage regimen (3–7%) [5, 6]. The low incidence of depression in the present trial (4%) is also consistent with phase 3 studies (4–5%). ALT flares >5 x ULN were reported in 3% of patients.

The incidence of HCC was lower in the S-Collate study than predicted by REACH-B, particularly in Asian patients and those without cirrhosis/transition to cirrhosis. However, given the low numbers, it is not known whether the emerging difference at 4 years is a robust trend, and studies with longer follow-up are required. The young age, small proportion with cirrhosis/transition to cirrhosis, and short duration of follow-up for detecting HCC also make interpretation difficult.

A real-world study such as S-Collate has inherent limitations, including the lack of a control group. It is not possible to state whether HBsAg clearance exceeds a baseline rate in untreated patients or in patients treated with NA. Treatment regimens were not strictly defined and many patients received parallel or overlapping NA treatment. Many patients had missing values, and genotype was unknown in most. Approximately 60% had no HBsAg result after 3 years’ follow-up, limiting any conclusions drawn. The results of this long-term observational study are consistent with those of large phase 3 studies and demonstrate that sustained off-treatment immune control is achieved in a proportion of patients treated with peginterferon alfa-2a. After 3 years’ follow-up, 5% of HBeAg-positive and 10% of HBeAg-negative patients with available data had experienced HBsAg clearance. Rates of HBsAg clearance increased in HBeAg-negative patients over the 3 years after treatment, and HBsAg clearance could be predicted by on-treatment HBsAg kinetics. Incidence of HCC was lower than predicted by REACH-B.

## Supporting information

S1 Checklist (DOCX)

S1 Appendix Trial information. (DOCX)

S2 Appendix Ethical approval–list of Independent Ethics Committees (IECs) or Institutional Review Boards (IRBs): S-Collate study. (DOCX)

S3 Appendix List of investigators: S-Collate study. (DOCX)

S1 Table Treatment regimens. (DOCX)

S2 Table HBsAg clearance rates in predefined subgroups (HBV genotype, treatment group, treatment duration) (mITT patients with available data). (DOCX)

S3 Table Estimates of HBsAg clearance rates in patients with available data and after using the multiple imputation methods for missing data. (DOCX)

S1 Data (PDF)

S1 File (PDF)
