# Supplementary material for: Final analysis of the international observational S-Collate study of peginterferon alfa-2a in patients with chronic hepatitis B
Source: PLoS One. 2020 Apr 10;15(4):e0230893. doi: 10.1371/journal.pone.0230893 (PMC7147799; doi:10.1371/journal.pone.0230893)
Supplement: S1 Appendix — (DOCX) [file pone.0230893.s002.docx]

**S1 appendix: Trial Information**

**Countries of enrollment:**

Austria, Bahrain, Bangladesh, Bosnia and Herzegovina, Bulgaria, China, Egypt, France, Germany, Hong Kong, India, Indonesia, Ireland, Jordan, Korea, Lebanon, Former Yugoslav Republic of Macedonia, Morocco, New Zealand, Pakistan, Poland, Portugal, Romania, Saudi Arabia, Thailand, and the United Kingdom.

**Planned subgroup analyses:**

Subgroups were defined according to hepatitis B virus (HBV) genotype (A, B, C, or D), and treatment duration (48, 72, or 96 weeks, or other).

An analysis was done by treatment regimen for those patients who received nucleos(t)ide analogs (NAs) before, during, or after treatment. Peginterferon monotherapy was defined as no prior treatment with an NA within 24 weeks prior to the start of peginterferon alfa-2a therapy, and no parallel NA treatment given during the first 12 weeks of therapy. Within this category, three subgroups were recognized: pure monotherapy (no NA treatment during or after treatment with peginterferon alfa-2a); NA post-peginterferon alfa-2a (started NA therapy at any time after completing peginterferon alfa-2a); and late parallel NA therapy (started NA therapy > 12 weeks after starting peginterferon alfa-2a and received the NA parallel to peginterferon alfa-2a for ≥ 12 weeks).

Combination peginterferon alfa-2a plus NA therapy was defined as no prior treatment with an NA within a timeframe of 24 to 4 weeks prior to the start of peginterferon alfa-2a therapy, and parallel NA therapy for at least 24 weeks that started no earlier than 4 weeks before, and no later than 12 weeks after the start of treatment. Peginterferon alfa-2a add-on (to NA) therapy was defined as the addition of peginterferon alfa-2a to ongoing NA therapy that had started ≥4 weeks prior to the initiation of peginterferon alfa-2a therapy and both were administered for at least 24 weeks in parallel. For patients who received NAs after the end of peginterferon alfa-2a treatment a HBV DNA value of <2000 IU/mL was not considered to be a response (i.e. a HBV DNA value of ≥2000 IU/mL was assigned) for all HBV DNA measurements obtained during or after NA treatment (unless the NA was discontinued within 8 weeks after the end of peginterferon alfa-2a therapy and prior to the HBV DNA determination in question).
