# Supplementary material for: Final analysis of the international observational S-Collate study of peginterferon alfa-2a in patients with chronic hepatitis B
Source: PLoS One. 2020 Apr 10;15(4):e0230893. doi: 10.1371/journal.pone.0230893 (PMC7147799; doi:10.1371/journal.pone.0230893)
Supplement: S2 Appendix — (DOCX) [file pone.0230893.s003.docx]

**S2 appendix**

**Ethical approval – List of independent ethics committees (IECs) or institutional review boards (IRBs): S-Collate study**

| **Country** | **IEC/IRB** | **City** |
| --- | --- | --- |
| Austria | Ethikkommission der Medizinischen Fakultaet der Universitaet Wien und des AKH der Stadt Wien | Wien |
| Bosnia and Herzegovina | Agencija za lijekove i medicinska sredstva | Banja Luka |
| Bosnia and Herzegovina | Ethic committee of Clinical Center University of Sarajevo | Sarajevo |
| Bosnia and Herzegovina | Ethics Committee Clinical Center Mostar | Mostar |
| Bosnia and Herzegovina | Ethics Committee Cantonal Hospital Zenica | Zenica |
| Bosnia and Herzegovina | Ethics Committee Clinical Center Banja Luka | Banja Luka |
| Bosnia and Herzegovina | Ethics Committee EC University Clinical Center Tuzla | Tuzla |
| Bulgaria | EC for Multicentral Trials | Sofia |
| China | EC of 2nd Affiliated Hospital, Chongqing Medical College | Chongqing |
| China | EC of Beijing 302 Hospital | Beijing |
| China | EC of Beijing Ditan Hospital | Beijing |
| China | EC of Beijing You An Hospital | Beijing |
| China | EC of Bethune International Peace Hospital | Shijiazhuang |
| China | EC of CTM Hospital of Xinjiang Vygur Autonomous Region | Urumqi |
| China | EC of Guangdong Provincial People's Hospital | Guangzhou |
| China | EC of Guangzhou Eighth Municipal People's Hospital | Guangzhou |
| China | EC of Hebei Medical University Third Hospital | Shijiazhuang |
| China | EC of Huashan Hospital | Shanghai |
| China | EC of Jinan Infectious Disease Hospital | Jinan |
| China | EC of Nanjing No.2 Hospital | Nanjing |
| China | EC of Peking University People's Hospital | Beijing |
| China | EC of Ruijin Hospital | Shanghai |
| China | EC of Shanghai Public Health Clinical Center | Shanghai |
| China | EC of Shengjing Hospital of China Medical University | Shenyang |
| China | EC of Shenzhen Third People's Hospital | Shenzhen |
| China | EC of Tangdu Hospital | Xi’an |
| China | EC of the 85 Hospital of PLA | Shanghai |
| China | EC of the First Affiliated Hospital of Wenzhou Medical College | Wenzhou |
| China | EC of the First Hospital of Harbin Medical University; Infection Department | Harbin |
| China | EC of The Sixth People's Hospital of Hangzhou | Hangzhou |
| China | EC of West China Hospital, Sichuan University | Chengdu |
| Germany | EK Baden-Württemberg LÄK | Stuttgart |
| Germany | EK Bonn | Bonn |
| Germany | EK der Albert-Ludwigs-Universität Freiburg | Freiburg |
| Germany | EK der Ärztekammer Nordrhein | Düsseldorf |
| Germany | EK Hamburg ÄK | Hamburg |
| Germany | EK Hannover | Hannover |
| Germany | EK Heidelberg | Heidelberg |
| Germany | EK Hessen LÄK | Frankfurt |
| Germany | EK München TU | München |
| Germany | EK Rheinland-Pfalz LÄK | Mainz |
| Germany | Ethik-Kommission der Ärztekammer Westfalen-Lippe u. der Med. Fakultät d. Westf. Wilhelms-Universität | Münster |
| Germany | Ethik-Kommission der Friedrich-Schiller-Universtät Jena an der Medizinischen Fakultät | Jena |
| Germany | Ethik-Kommission der Medizinischen Fakultät der Friedrich-Alexander-Universität Erlangen-Nürnberg | Erlangen |
| Germany | Ethik-Kommission Medizinische Fakultät der Universität Essen | Essen |
| Hong Kong | NTW Cluster Clinical & Research Ethics Committee | Hong Kong |
| Indonesia | Ethic Committee University of Indonesia | Central Jakarta |
| Korea, Republic of | Ajou University Hospital; IRB | Gyeonggi-do |
| Korea, Republic of | Chungang University Hospital; IRB | Seoul |
| Korea, Republic of | Chungbuk National University Hospital; IRB | Chungcheongbuk-do |
| Korea, Republic of | Chungnam National University Hospital; IRB | Daejeon |
| Korea, Republic of | Daejin Medical Center Bundang Jesaeng General Hospital; IRB | Gyeonggi-do |
| Korea, Republic of | Eulji University Eulji Medical Centre; IRB | Seoul |
| Korea, Republic of | Hallym University Medical Center Chuncheon Sacred Heart Hospital; IRB | Gangwon-do |
| Korea, Republic of | Hallym University Sacred Heart Hospital IRB | Gyeonggi-do |
| Korea, Republic of | Inje University Busan Paik Hospital; IRB | Busan |
| Korea, Republic of | IRB, Inha Uni Hospital | Incheon |
| Korea, Republic of | IRB of Korea Univ Guro Hospital; IRB | Seoul |
| Korea, Republic of | Kyungpook National University Hospital; IRB | Daegu |
| Korea, Republic of | Pusan National University Hospital; IRB | Busan |
| Korea, Republic of | Samsung Medical Center EC | Seoul |
| Korea, Republic of | Severance Hospital-Yonsei University; IRB | Seoul |
| Korea, Republic of | Ulsan University Hospital; IRB | Ulsan |
| Macedonia | Eticka komisija, Agencija za lekovi, Ministersvo za zdravstvo na Republika Makedonija | Skopje |
| Morocco | Comité d'Ethique de la Recherche Biomédicale; Faculté de Médecine et de Pharmacie de Rabat | Rabat |
| New Zealand | Northern X Ethics Committee | Auckland |
| Portugal | Comissão de Ética Hospital Santa Maria | Lisboa |
| Portugal | Comissão de Ética para a Saúde do Hospital Geral | Porto |
| Portugal | Comissão de Ética para a Saúde; Hosp. Sto António dos Capuchos | Lisboa |
| Portugal | Comissão de Ètica para a Saúde - HSJ | Porto |
| Romania | Comisia Nationala de Etica | Bucharest |
| Saudi Arabia | King Fahad Medical City-IRB | Riyadh |
| Saudi Arabia | King Faisal Specialist Hospital & Research Centre; Oncology; Kfsh&Rc-Office of Research Affairs(ORA) | Riyadh |
| Saudi Arabia | North West Armed Forces Hospital; Internal Medicine; NWAFH-Research Ethics Committee | Tabuk |
| Thailand | Research Ethics Com. Fac Med. Chiang Mai University | Chaing Mai |
| Thailand | Siriraj Institutional Review Board | Bangkok |
| Thailand | Songklanagarind Ethics Committee | Songkla |
| United Kingdom | NRES Committee London-City & East | London |
