# Supplementary material for: Final analysis of the international observational S-Collate study of peginterferon alfa-2a in patients with chronic hepatitis B
Source: PLoS One. 2020 Apr 10;15(4):e0230893. doi: 10.1371/journal.pone.0230893 (PMC7147799; doi:10.1371/journal.pone.0230893)
Supplement: S3 Appendix — (DOCX) [file pone.0230893.s004.docx]

**List of investigators: S-Collate study**

| **Investigator** | **Center** |
| --- | --- |
| Maieron, Andreas | A.Ö. Krankenhaus der Elisabethinen Linz, Linz, Austria |
| Stauber, Rudolf | Lkh-Univ. Klinikum Graz, AuenbGraz, Austria |
| Vogel, Wolfgang | Tiroler Landeskrankenanstalten Ges.M.B.H, Innsbruck, Austria |
| Ferenci, Peter | Universitaetsklinik Fuer Innere Medizin Iii, Wien, Austria |
| Gschwantler, Michael | Wilhelminenspital, Wien, Austria |
| Habib, Jaffer | Salmaniya Medical Complex, Manama, Bahrain |
| Rahman, Salimur | Liver Clinic Lab-Aid Specialized Hospital, Dhaka, Bangladesh |
| Mobin, Khan | The Liver Centre, Dhaka, Bangladesh |
| Ibrahimpasic, Nevzeta | Cantonal Hospital Bihac, Bihac, Bosnia and Herzegovina |
| Calkic, Leila | Cantonal Hospital Zenica, Zenica, Bosnia and Herzegovina |
| Kezic, Zdravka  Verhaz, Antonija | Clinical Center Banja Luka, Banjaluka, Bosnia and Herzegovina |
| Vukobrat-Bijedic, Zora | Clinical Center Sarajevo, Sarajevo, Bosnia and Herzegovina |
| Bevanda, Milenko | Clinical Hospital Mostar, Mostar, Bosnia and Herzegovina |
| Zerem, Enver  Ahmetagic, Sead | University Clinical Center Tuzla, Tuzla, Bosnia and Herzegovina |
| Silva, Ana Heloisa | Ambulatório De Hepatites Virais De Foz Do Iguaçu, Foz do Iguacu, Brazil |
| Lindenberg, Andrea | Centro De Doenças Infecto Parasitárias, Campo Grande, Brazil |
| Crespo, Deborah | Clinica De Diagnosticos Medicos, Belem, Brazil |
| Victoria, Flamir | Fundação Medicina Tropical Do Amazonas, Manaus, Brazil |
| Goncales Junior, Fernando | Hospital das Clinicas – UNICAMP, Campinas, Brazil |
| Araujo, Ana Ruth  Rocha, Cristina | Hu Getúlio Vargas - Universidade Federal Do Amazonas, Manaus, Brazil |
| Salcedo, Juan Miguel | Instituto De Pesquisa Em Patologias Tropicais E Cepem, Porto Velho, Brazil |
| Lobato, Cirley Maria | Serviço De Assistência Especializada, Rio Branco, Brazil |
| Franca, Alex | Universidade Federal de Sergipe – UFS, Aracajú, Brazil |
| Martins Junior, Elson | Universidade Metropolitana De Santos, Santos, Brazil |
| Kotzev, Iskren | MHAT Sveta Marina, Varna, Bulgaria |
| Balabanska, Rozalina | MHAT Tokuda Hospital Sofia, Sofia, Bulgaria |
| Gao, Rentao | Anhui Provincial Hospital, He Fei, China |
| Zhang, Hongfei | Beijing 302 Hospital, Beijing, China |
| Xie, Yao | Beijing Ditan Hospital, Beijing, China |
| Chen, Xin Yue | Beijing You An Hospital; Beijing, China |
| Sun, Dianxing | Bethune International Peace Hospital of PLA, Shijiazhuang, China |
| Chen, Xiao-Ping | Guangdong General Hospital, Guangzhou, China |
| Yang, Zhan | Guangzhou Eighth Municipal People's Hospital, Guangzhou, China |
| Li, Xiaoou | Hangzhou Sixth People's Hospital, Hangzhou, China |
| Zhang, Jiming | Huashan Hospital Affiliated to Fudan University, Shanghai, China |
| Chen, Shi Jun | Jinan Infectious Diseases Hospital, Jinan, China |
| Zhao, Wei | Nanjing No.2 Hospital, Nanjing, China |
| Wei, Lai | Peking University People's Hospital, Beijing, China |
| Xie, Qing | Ruijin Hospital, Shanghai Jiao Tong University School of Medicine, Shanghai, China |
| Wu, Shan Ming | Shanghai Public Health Clinical Center, Shanghai, China |
| Dou, Xiao Guang | Shengjing Hospital of China Medical University, Shenyang, China |
| Lu, Jian | Shenzhen Donghu Hospital, Shenzhen, China |
| Li, Yongguo | The 1st Affiliated Hospital of Harbin Medical University, Harbin, China |
| Chengwei, Chen i | The 85th Hospital of P.L.A., Shanghai, China |
| Sheng, Ji-Fang | The First Affiliated Hospital of College of Medicine, Zhejiang University, Hangzhou, China |
| Chen, Yongping | The First Affiliated Hospital of Wenzhou Medical College, Wenzhou, China |
| Hong, Ren | The Second Affiliated Hospital, Chongqing Medical University, Chongqing, China |
| Sun, Yongtao | The Second Affiliated Hospital of The Fourth Military Medical University (Tangdu Hospital), Xian, China |
| Gao, Zhi Liang | The Third Affiliated Hospital of Sun Yat-Sen University, Guangzhou, China |
| Zhao, Caiyan | The Third Hospital of Hebei Medical University, Shi Jiazhuang, China |
| Ning, Qin | Tongji Hosp, Tongji Med. Col, Huazhong Univ. of Sci. & Tech, Wuhan, China |
| Tang, Hong | West China Hospital, Sichuan University, Chengdu, China |
| Wang, Xiaozhong | Xinjiang Uygur Autonomous Region Hospital of Chinese Traditional Medicine, Urumchi, China |
| Monis, Ahmed | Dr. Ahmed Monis Clinic Misr Insurance Building, Giza, Egypt |
| Mostafa, Ibrahim | Dr. Ibrahim Mostafa Clinic, Cairo, Egypt |
| Affifi, Mohamed | Dr. Mohamed Amr Affifi Clinic, Cairo, Egypt |
| El Amir, Mona | Dr. Mona el Amir Private Clinic, Giza, Egypt |
| Fawzy, Nabil | Dr. Nabil Fawzy Private Clinic, Cairo, Egypt |
| Elansary, Nadia | Dr. Nadia Elansary Clinic, Cairo, Egypt |
| Elzanaty, Taher | Dr. Taher Elzanaty Clinic, Giza, Egypt |
| Sheha, Gamal | Gamal Sheha Clinic, Mansoura, Egypt |
| Elshazly, Yehia | Yehia Elshazly Clinic for Hepatology and Gastroenterology, Cairo, Egypt |
| Gasser, Patrick | Cabinet, Nantes, France |
| Delasalle, Patrick | Cabinet, Grasse, France |
| Hanslik, Bertrand | Cabinet Medical, Montpellier, France |
| Constant, Thierry | Cabinet Medical Gastro Enterologie, Toulon, France |
| Buffet, Catherine | CH De Bicetre, Bicetre, France |
| Macaigne, Gilles | CH De Lagny Sur Marne, Lagny sur Marne, France |
| Payen, Jean Louis | CH De Montauban, Montauban, France |
| Pilette, Christophe | CH Du Mans; Le Mans, France |
| Picon, Magali,  Wartelle Bladou, Claire | CH Du Pays D Aix, Aix en Provence, France |
| Pauwels, Arnaud | CH Emmanuel Rain, Gonesse, France |
| Descamps, Jean Michel | CH Georges Renon, Niort, France |
| Arpurt, Jean-Pierre  Coulibaly Baya | CH Henri Duffaut, Avignon, France |
| Kerneis, Jean | CHI De Cornouaille, Quimper, France |
| Cadranel, Jean-François | CH Laennec, 60100 Creil, France |
| Fontanges, Thierry | CH Pierre Oudot, Bourgoin Jallieu, France |
| Benhamou, Yves  Moussalli, Joseph  Ratziu, Vlad | CH Pitie Salpetriere, Paris, France |
| Lambare, Benedicte | CH Sud Francilien, Evry, France |
| Bonny Monier, Corinne | CHU Estaing, Clermont-Ferrand, France |
| Perarnau, Jean Marc | CHU Trousseau, Chambray Les Tours, France |
| Joseph Reinette, Cathia  Renard, Philippe | CH Victor Dupouy, Argenteuil, France |
| Combis, Jean Marc | Clinique Ambroise Pare, Toulouse, France |
| Beorchia, Sylvain | Clinique De La Sauvegarde, Lyon, France |
| Bourliere, Marc  Castellani, Paul | Fondation Hôpital Saint Joseph, Marseille, France |
| Lunel Fabiani, Francoise | Fondation Institut Arthur Vernes, Paris, France |
| Coton, Thierry  Guisset, Michel | HIA Alphonse Laveran, Marseille, France |
| Leroy, Vincent  Zarski, Jean-Pierre | Hôpital Albert Michallon, La Tronche, France |
| Naveau, Sylvie | Hôpital Antoine Beclere, Clamart, France |
| Roulot Marullo, Dominique | Hôpital Avicenne, Bobigny, France |
| Asselah, Tarek  Boyer Darrigrand, Nathalie  Castelnau Marchand, Corinne  Giuily Guigui Nathalie  Marcellin, Patrick  Moucari, Rami  Ripault, Marie Pierre | Hôpital Beaujon, Clichy, France |
| Pospait, Dan | Hôpital Bichat Claude Bernard, Paris, France |
| Ribard, Didier | Hôpital Caremeau, Nimes, France |
| Goriadumont, Odile  Riachi, Ghassan | Hôpital Charles Nicolle, Rouen, France |
| Doffoel, Michel  Habersetzer, Francois | Hôpital Civil, Strasbourg, France |
| Canva Delcambre, Valerie  Mathurin, Philippe | Hôpital Claude Huriez, Lille, France |
| Fontaine, Helene | Hôpital Cochin, Paris, France |
| Dao, Thong  Guillemard, Catherine  Ollivier Hourmand, Isabelle | Hôpital Cote De Nacre, Caen, France |
| Borentain, Patrick  Botta Fridlund, Daniele  Gerolami Santandrea, Rene  Portal, Isabelle | Hôpital De La Conception, Marseille, France |
| Zoulim, Fabien | Hôpital De La Croix Rousse, Lyon, France |
| Tran, Albert | Hôpital De L'Archet, Nice, France |
| Danielou Satre, Helene  Guyader, Dominique | Hôpital De Pontchaillou, Rennes, France |
| Hillon, Patrick | Hôpital Du Bocage, Dijon, France |
| De Ledinghen, Victor | Hôpital Du Haut-Leveque, Pessac, France |
| Loustaud Ratti, Veronique | Hôpital Dupuytren, Limoges, France |
| Bader, Robert | Hôpital Emile Muller, Mulhouse, France |
| Haddad, Nabil | Hôpital Esquirol, St Maurice, France |
| Bernardini, David  Chinoune, Farid  Escudie, Luc | Hôpital Font Pre, Toulon, France |
| Hezode, Christophe  Mallat, Ariane | Hôpital Henri Mondor, Creteil, France |
| Feray, Cyrille  Gournay, Jerome | Hôpital Hotel Dieu Et Hme, Nantes, France |
| Bizollon, Thierry | Hôpital Hotel Dieu, Lyon, France |
| Tranvouez, Jean Luc | Hôpital Jacques Monod, Montivilliers, France |
| Cervoni, Jean Paul  Di Martino, Vincent  Theveno, Thierry | Hôpital Jean Minjoz, Besancon, France |
| Beaugrand, Michel  Trinchet, Jean-Claude | Hôpital Jean Verdier, Bondy, France |
| Nousbaum, Jean Baptiste | Hôpital La Cavale Blanche, Brest, France |
| Mouly, Stephane | Hôpital Lariboisiere, Paris, France |
| Assor, Philippe  Causse, Xavier  Si Ahmed, Si Nafa | Hôpital La Source, Orleans, France |
| Thomann Weiss, Anne Marie | Hôpital Louis Pasteur, Colmar, France |
| Le Guillou, Brigitte  Raabe, Jean Jacques | Hôpital N D Bon Secours, Metz, France |
| Pol, Stanislas | Hôpital Necker - Enfants Malades, Paris, France |
| Nguyen Khac, Eric Duc | Hôpital Nord Medecine A, Amiens, France |
| Capron Chivrac, Dominique | Hôpital Nord; Reseau Hepatologie Picardie, Amiens, France |
| Duclos Vallee, Jean Charles  Samuel, Didier | Hôpital Paul Brousse, Villejuif, France |
| Barange, Karl  Alric, Laurent  Metivier, Sophie | Hôpital Purpan Toulouse, France |
| Bernard Chabert, Brigitte  Heurgue, Alexandra  Thiefin, Gerard | Hôpital Robert Debre, Reims, France |
| Bernard, Pierre Henri  Castera, Laurent  Foucher Guimard, Juliette | Hôpital Saint Andre, Bordeaux, France |
| Andreani, Tony  Carbonell, Nicolas  Heymann Fartoux, Laetitia  Lemoine, Maud  Serfaty, Lawrence | Hôpital Saint Antoine, Paris, France |
| Blanc, Pierre  Larrey, Dominique  Pageaux, Georges-Philippe | Hôpital Saint-Eloi, Montpellier, France |
| Remy, Andre Jean | Hôpital Saint Jean, Perpignan, France |
| Bacq, Yannick  D Alteroche, Louis | Hôpital Trousseau, Chambray Les Tours, France |
| Bronowicki, Jean-Pierre | Hopitaux De Brabois, Vandoeuvre-Les-Nancy, France |
| Cales, Paul  Fouchard Hubert, Isabelle  Lunel Fabiani, Francoise  Obert, Frederic | Hotel Dieu, Angers, France |
| Ouzan Denis | Institut Arnault Tzanck, Saint Laurent Du Var, France |
| Mayr, Christoph | Ärzteforum Seestraße Dres.Frank Strohbach, Christoph Mayr, Berlin, Germany |
| Bauditz, Jürgen  Schott, Eckart | Campus Virchow-Klinikum Charité Centrum 13, Berlin, Germany |
| Somasundaram, Rajan | Charité - Campus Benjamin Franklin, Berlin, Germany |
| Dörffel, Yvonne | Charité Universitätsmedizin Berlin, Berlin, Germany |
| Sarhaddar, Jasmin | Diakoniekrankenhaus Medizinische Klinik II, Germany |
| Kaiser, Stephan  Schnaitmann, Eiko | Dres. Andreas Schaffert Andreas Trein Und Edith, Stuttgart, Germany |
| Möller, Bernd | Dres.Bernd Möller Und Renate Heyne, Berlin, Germany |
| Hueppe, Dietrich | Dres.Dietrich Hüppe Gisela Felten Und Heinz Hartmann, Herne, Germany |
| Naumann, Uwe | Dres. Jörg Gölz Und Arend Moll, Berlin, Germany |
| Christensen, Stefan | Dr.Med.Heiner W.Busch Und Stefan Christensen, Münster, Germany |
| Fenske, Stefan | Infektionsmedizinisches Centrum Hamburg Ich Grindel, Hamburg, Germany |
| Weber, Andreas | Klinikum Nord Medizinische Klinik 6, Nürnberg, Germany |
| Geisler, Fabian | Klinikum Rechts Der Isar Der Tu München, München, Germany |
| Jablonowski, Helmut | Klinikum Salzgitter-Lebenstedt Medizinische Klinik I, Salzgitter, Germany |
| Zachoval, Reinhart | Ludwig-Maximilians-Universitätsklinik Großhadern, Muenchen, Germany |
| Wedemeyer, Hans Heinrich | Medizinische Hochschule Zentrum Innere Medizin, Hannover, Germany |
| Klinker, Hartwig | Medizinische Universitätsklinik, Wuerzburg, Germany |
| Petersen, Jörg | Med. Versorgungszentrum Ifi-Institut, Hamburg, Germany |
| Heyne, Renate | Praxis Dr. Heyne, Berlin, Germany |
| John, Christine | Praxis Dr. Med. Christine John, Berlin, Germany |
| Teuber, Gerlinde | Praxis Pd Dr.Med. Gerlinde Teuber, Frankfurt Am Main, Germany |
| Mauruschat, Sabine | Praxis Sabine Mauruschat, Wuppertal, Germany |
| Link, Ralph | St. Josefs Klinik, Offenburg, Germany |
| Schmidt, Josef-Peter | St. Marien-Hospital Klinik, Hamm, Germany |
| Baumgarten, Axel | Überörtl.Gem.Praxis Dres. Stephan Dupke Axel Baumgarten, Berlin, Germany |
| Schuchmann, Marcus | Uni. Der Johannes Gutenberg-Universitaet, Mainz, Germany |
| Eisenbach, Christoph | Uni Heidelberg Med. Klinik, Heidelberg, Germany |
| Grambihler, Anette | Uniklinik Mainz, Mainz, Germany |
| Gerken, Guido | Universitaetsklinikum Essen, 22 Essen, Germany |
| Spengler, Ulrich | Universitätsklinikum Bonn, Bonn, Germany |
| Strobel, Deike | Universitätsklinikum Erlangen; Erlangen, Germany |
| Thimme, Robert | Universitätsklinikum Freiburg Medizinische Klinik,Freiburg, Germany |
| Discher, Thomas | Universitätsklinikum Gießen und Marburg GmbH, Giessen, Germany |
| Herrmann, Andreas  Stallmach, Andreas | Universitätsklinikum Jena, Jena, Germany |
| Erhardt, Andreas | Universitätsklinikum Klinik f. Gastroenterologie Hepatologie und Infektiologie, Düsseldorf, Germany |
| Malfertheiner, Peter | Universitätsklinikum Magdeburg, Magdeburg, Germany |
| Schmidt, Hartmut Hans-Jürgen | Universitätsklinikum Münster Klinik und Poliklinik für Transplantationsmedizin, Münster, Germany |
| Schmidt, Klaus | Universitätsklinikum Schleswig-Holstein/Campus Lübeck, Lübeck, Germany |
| Günther, Rainer | Universitätsklinikum S.-H. Campus Kiel Klinik für Innere Medizin, Kiel, Germany |
| Klass, Dietmar M. | Universitätsklinikum Ulm, Ulm, Germany |
| Ramadori, Giuliano | Universitätsmedizin Göttingen Georg-August- Universität5, Göttingen, Germany |
| Li, Kin Kong | Tuen Mun Hospital, Hong Kong |
| Sanjay, Jain | Indraprastha Apollo Hospitals, New Delhi, India |
| Goswami, Bhabadev | Institute of Digestive Diseases, Guwahati, India |
| Thorat, Vinay | Krishnai Clinic, Pune, India |
| Sud, Randhir | Medanta -The Medicity, Gurgaon, India |
| Gadhikar, Harshal | Medipoint Clinic, Pune, India |
| Shah, Samir | Opp Jaslok Hospital, Mumbai, India |
| Pai, Nitin | Pai Clinic and Diagnostic Center, Pune, India |
| Shenoy, KT | Sree Gokulam Medical College and Research Foundation, Trivandrum, India |
| Gani, Rino A | Bintaro International Hospital, Tangerang, Indonesia |
| Bestari, Begawan | Boromeus Hospital, Bandung, Indonesia |
| Lelosutan, Sjaffrudin | Central Army Hospital Rspad Gatot Soebroto, Jakarta, Indonesia |
| Hasan, Irsan | Cipto Mangunkusumo General Hospital, Jakarta, Indonesia |
| Boedi, Poernomo | Dr. Soetomo Hospital; Kidney and Hipertansion, Surabaya, Indonesia |
| Djumhana, Ali | Hasan Sadikin Hospital; Digestive Surgery, Bandung, Indonesia |
| Sanityoso, Andri | Klinik Hati, Jakarta, Indonesia |
| Budihusodo, Unggul | Klinik Kimia Farma, Jakarta, Indonesia |
| Lesmana, Laurentius | Medistra Hospital, Jakarta, Indonesia |
| Noer, Sjaifoellah | Mitra International Jatinegara Hospital, Jakarta, Indonesia |
| Akbar, Nurul | Pgi Cikini Hospital, Jakarta, Indonesia |
| Djajadiredja, HR Syarief H. | Santosa Bandung International Hospital, Bandung, Indonesia |
| Lambert, Jack | Mater Misericordiae University Hospital, Dublin, Ireland |
| McKiernan, Susan | St. James Hospital; Dublin, Ireland |
| Hamoudi, Waseem | Al-Bashir Hospital, Amman, Jordan |
| Jadallah, Khaled | King Abdullah University Hospital, Irbid, Jordan |
| Nusair, Majed | Prince Hamzeh Hospital; Amman, Jordan |
| Cho, Sungwon | Ajou Uni Medical Centre; Seoul, South Korea |
| Seonghun, Kim | Chonbuk National Uni Hospital, Jeonju, South Korea |
| Kim, Dong Joon | Chooncheon Sacred Heart Hospital, Chooncheon, South Korea |
| Kim, Hyungjoon | Chungang University Hospital, 2Seoul, South Korea |
| Lee, Byungseok | Chungnam University Hospital, Daejeon, South Korea |
| Ahn, Sang Bong | Eulji General Hospital, Seoul, South Korea |
| Lee, Jinwoo | Inha University Hospital, Incheon, South Korea |
| Lee, Younjae | Inje University Pusan Paik Hospital, Busan, South Korea |
| Yeon, Jongeun | Korea Kuro Hospital, Seoul, South Korea |
| Kweon, Young-Oh | Kyungpook National Uni Hospital, Daegu, South Korea |
| Park, Young Min | Pundang Jesaeng General Hospital Seoungnamsi, South Korea |
| Heo, Jeong | Pusan University Hospital Busan, South Korea |
| Park, Choong Kee | Pyungchon Sacred Heart Hospital, Anyang, South Korea |
| Paik, Seung Woon | Samsung Medical Centre; Seoul, South Korea |
| Yoon, Junghwan | Seoul National Uni Hospital Seoul, South Korea |
| Park, Neunghwa | Ulsan University Hospital, Ulsan, South Korea |
| Han, Kwang-Hyub | Yonsei Uni College of Medicine, Seoul, South Korea |
| Sharara, Ala | American University of Beirut - Medical Center, Beirut, Lebanon |
| Issa, Iyad | Beirut Governmental University Hospital, Beirut, Lebanon |
| Sayegh, Raymond | Hotel-Dieu De France Hospital, Beirut, Lebanon |
| Rassam, Paul | Saint Georges Hospital, Beirut, Lebanon |
| Joksimovic, Nenad  Ivanovsk, Ljubomir | Clinical Center Skopje, Skopje, FYR Macedonia |
| Jamil, Driss | Cabinet Privé Pr D Jamil, Casablanca, Morocco |
| Abdellah, Essaid | Centre Hospitalier Universitaire Ibn Sina, Rabat, Morocco |
| Ibrahimi, Adil | CHU Hassan 2; Fes, Morocco |
| Gane, Edward | Auckland Hospital, Auckland, New Zealand |
| Moyes, Chris | Hepatitis Foundation, Whakatane, New Zealand |
| Dickson, Graeme | Waikato Hospital, Hamilton, New Zealand |
| Abbas, Zaigham | Akbar Center, Karachi, Pakistan |
| Shaukat, Aamir | Allied Hospital, Faisalabad, Pakistan |
| Mirza, Shakeel | Combined Military Hospital, Rawalpindi, Pakistan |
| Fayaz, Badar | Dow University of Health Sciences, Karachi, Pakistan |
| Umar, Muhammad | Holy Family Hospital, Rawalpindi, Pakistan |
| Gill, Muzaffar | Islamabad Specialist Clinic, Islamabad, Pakistan |
| Memon, Sadik | Isra University Department of Medicine, Hyderabad, Pakistan |
| Alam, Altaf | Kanaan Clinic 121, Lahore, Pakistan |
| Ashraf, Pervaiz | Liaqat National Hospital, Karachi, Pakistan |
| Memon, Rauf | Medicare Clinics, Room 110, 1st Floor, 22 Shaheed-E-Millat Road, Karachi, Pakistan |
| Farooqi, Javed | Saeed Anwar Medical Centre, Peshawar, Pakistan |
| Khan, Anwaar | Sheikh Zayed Hospital, Lahore, Pakistan |
| Gietka, Andrzej | Centralny Szpital Kliniczny Mswia, Warszawa, Poland |
| Plesniak, Robert | Centrum Medyczne, Lancut, Poland |
| Halota, Waldemar | Hospital for Infectious Diseases, Bydgoszcz, Poland |
| Berak, Hanna | Inst. Of Infectious & Parasitic Diseases, Warszawa, Poland |
| Baka-Cwierz, Barbara | Krakowski Szpital Specjalistyczny Im. Jana Pawla, Krakow, Poland |
| Flisiak, Robert | Medical Uni Of Bialystok, Bialystok, Poland |
| Jablkowski, Maciej | Medical Uni Of Lodz, Lodz, Poland |
| Lojewski, Władysław | Nzoz Lubuska Specjalistyczna Poradnia Chorob Watroby, Zielona Góra, Poland |
| Radowska, Danuta | Pomorskie Centrum Chorob Zakaznych I Gruzlicy, Gdansk, Poland |
| Piekarska, Anna | Specjalistyczny Szpital Wojewódzki Im. Biegańskiego, Łodz, Poland |
| Mazur, Wlodzimierz | Szpital Specjalistyczny, Chorzow, Poland |
| Deron, Zbigniew | Wojewódzki Specjalistyczny Szpital Im. Bieganskiego, Lodz, Poland |
| Gladysz, Andrzej | Wojewodzki Szpital, Wroclaw, Poland |
| Marinho, Rui Tato | Hospital De Santa Maria, Lisboa, Portugal |
| Macedo, Guilherme | Hospital De Sao Joao, Porto, Portugal |
| Pedroto, Isabel | Hospital Geral De Santo Antonio, Porto, Portugal |
| Calinas, Filipe | Hospital Santo Antonio Dos Capuchos, Lisboa, Portugal |
| Ancuta, Ioan | Cantacuzino Clinical Hospital, Bucharest, Romania |
| Curescu, Manuela | Clinical Infectious Diseases Hospital Victor Babes, Timisoara, Romania |
| Caruntu, Florin  Streinu-Cercel, Adrian | Institutul De Boli Infectioase Matei Bals, Bucharest, Romania |
| Diaconescu, Iulian | Spitalul Clinic De Boli Infectioase Si Tropicale Dr. Victor, Craiova, Romania |
| Motoc, Adriana | The Hospital of Tropical And Infectious Disease Victor Babes, Bucharest, Romania |
| Hamouda, Abdellatif | Dammam Central Hospital, Dammam, Saudi Arabia |
| Ageely, Hussein | King Fahad Central Hospital, Jizan, Saudi Arabia |
| Alomair, Ahmed | King Fahad Medical City, Riyadh, Saudi Arabia |
| Almomen, Sami | King Fahad Specialist Hospital, Dammam, Saudi Arabia |
| Ismail, Mona | King Fahad University Hospital Alkhobar, Saudi Arabia |
| Alashgar, Hamad | King Faisal Specialist Hospital & Research Centre, Riyadh, Saudi Arabia |
| Abdo, Ayman | King Khaled Uni Hospital Riyadh, Saudi Arabia |
| Noorkhan, Naveed | North West Armed Forces Hospital, Tabuk, Saudi Arabia |
| Almadni, Abdullah | Riyadh Military Hospital, Po Box 7897, 11159 Riyadh, Saudi Arabia |
| Thongsawat, Satawat | Chiang Mai Uni Hospital, Chiang Mai, Thailand |
| Tanwandee, Tawesak | Siriraj Hospital, Bangkok, Thailand |
| Piratvisuth, Teerha | Songklanagarind Hospital, Songkhla, Thailand |
| Sidahmed Elhassan Yousif, Elhassan | Rashid Hospital, Dubai, UAE |
| Abouda, George | Hull Royal Infirmary, Hull, UK |
| Brown, Ashley | St Mary's Hospital, London, UK |
| Agarwal, Kosh | King's College Hospital, London, UK |
| Prince, Martin | Manchester Royal Infirmary, Manchester, UK |
| Forton, Daniel | St. Georges Hospital, London, UK |
| Foster, Graham | The Royal London Hospital, London, UK |
| Rosenberg, William | University College London, London, UK |
