# Supplementary material for: Final analysis of the international observational S-Collate study of peginterferon alfa-2a in patients with chronic hepatitis B
Source: PLoS One. 2020 Apr 10;15(4):e0230893. doi: 10.1371/journal.pone.0230893 (PMC7147799; doi:10.1371/journal.pone.0230893)
Supplement: S1 Table — (DOCX) [file pone.0230893.s005.docx]

**S1** **Table. Treatment regimens.**

| **Regimen, n (%)** | **HBeAg-positive**  **n=844** | **HBeAg-negative**  **n=872** |
| --- | --- | --- |
| PegIFN monotherapy | 697 (83) | 753 (86) |
| Pure monotherapy | 429 (51) | 492 (56) |
| PegIFN monotherapy + post-therapy NA | 219 (26) | 253 (29) |
| PegIFN monotherapy + late-parallel NA | 49 (6) | 8 (<1) |
| Parallel therapy | 58 (7) | 78 (9) |
| Combination (PegIFN + NA) | 28 (3) | 31 (4) |
| PegIFN add-on to NA | 30 (4) | 47 (5) |
| Other | 89 (11) | 41 (5) |

HBeAg, hepatitis B e antigen; PegIFN, peginterferon alfa-2a; NA, nucleos(t)ide analog.
