# Supplementary material for: Final analysis of the international observational S-Collate study of peginterferon alfa-2a in patients with chronic hepatitis B
Source: PLoS One. 2020 Apr 10;15(4):e0230893. doi: 10.1371/journal.pone.0230893 (PMC7147799; doi:10.1371/journal.pone.0230893)
Supplement: S2 Table — (DOCX) [file pone.0230893.s006.docx]

**S2** **Table.** **HBsAg clearance rates in predefined subgroups (HBV genotype, treatment group, treatment duration) (mITT patients with available data).**

| **Subgroup, n/N (%)** | **HBsAg clearance** | | | |
| --- | --- | --- | --- | --- |
|  | **HBeAg-positive**  **n=844** | | **HBeAg-negative**  **n=872** | |
|  | mITT | mITT with available data | mITT | mITT with available data |
| HBV genotype | | | | |
| A | 3/22 (14) | 3/12 (25) | 4/35 (11) | 4/11 (36) |
| B | 1/43 (2) | 1/16 (6) | 3/23 (13) | 3/11 (27) |
| C | 1/94 (1) | 1/32 (3) | 0/17 (0) | 0/7 (0) |
| D | 0/67 (0) | 0/26 (0) | 1/116 (<1) | 1/53 (2) |
| Other/mixed genotype | 0/16 (0) | 0/8 (0) | 1/29 (3) | 1/14 (7) |
| Unknown genotype | 11/602 (2) | 11/234 (5) | 32/652 (5) | 32/298 (11) |
| Treatment group | | | | |
| PegIFN monotherapy | 14/697 (2) | 14/261 (5) | 31/753 (4) | 31/334 (9) |
| Pure monotherapy | 10/429 (2) | 10/118 (8) | 31/492 (6) | 31/169 (18) |
| Post-therapy NA | 3/219 (1) | 3/118 (3) | 0/253 (0) | 0/160 (0) |
| Late-parallel NA | 1/49 (2) | 1/25 (4) | 0/8 | 0/5 (0) |
| Parallel therapy |  |  |  |  |
| Combination (PegIFN + NA) | 0/28 (0) | 0/9 (0) | 1/31 (3) | 1/12 (8) |
| PegIFN add-on to NA | 1/30 (3) | 1/13 (8) | 5/47 (11) | 5/27 (19) |
| Other | 1/89 (1) | 1/45 (2) | 4/41 (10) | 4/21 (19) |
| Treatment duration | | | | |
| ≥ 48 weeks | 9/379 (2) | 9/169 (5) | 31/628 (5) | 31/296 (10) |
| ≥ 72 weeks | 3/44 (7) | 3/24 (13) | 2/15 (13) | 2/9 (22) |
| ≥ 96 weeks | 1/32 (3) | 1/17 (6) | 1/12 (8) | 1/6 (17) |

HBeAg, hepatitis B e antigen; HBsAg, hepatitis B surface antigen; HBV, hepatitis B virus; mITT, modified intention-to-treat; NA, nucleos(t)ide; PegIFN, peginterferon alfa-2a.
