# Supplementary material for: Final analysis of the international observational S-Collate study of peginterferon alfa-2a in patients with chronic hepatitis B
Source: PLoS One. 2020 Apr 10;15(4):e0230893. doi: 10.1371/journal.pone.0230893 (PMC7147799; doi:10.1371/journal.pone.0230893)
Supplement: S3 Table — (DOCX) [file pone.0230893.s007.docx]

**S3** **Table. Estimates of HBsAg clearance rates in patients with available data, using GEE models for longitudinal data with missing values and after using the multiple imputation methods for missing data.**

|  | **Patients with available data, n/N  % (95% CI)** | | **GEE analysis for longitudinal data with missing values, % (95% CI)** | | **50 multiple imputations for missing values in longitudinal data,  % (95% CI)** | |
| --- | --- | --- | --- | --- | --- | --- |
|  | **HBeAg-positive** | **HBeAg-negative** | **HBeAg-positive** | **HBeAg-negative** | **HBeAg-positive** | **HBeAg-negative** |
| End of treatment | 23/570  4 (3–6) | 30/641 5 (3–7) | 4 (3–6) | 6 (4–8) | 5 (2–7) | 5 (3–7) |
| 6 months post-treatment | 27/501 5 (4–8) | 37/540 7 (5–9) | 5 (3–6) | 7 (5–9) | 6 (4–8) | 8 (5–10) |
| 1 year post-treatment | 25/431 6 (4–8) | 39/486 8 (6–11) | 6 (4–8) | 8 (6–10) | 6 (4–9) | 9 (6–11) |
| 2 years post-treatment | 21/414 5 (3–8) | 32/440 7 (5–10) | 6 (4–7) | 9 (7–11) | 6 (3–8) | 8 (5–11) |
| 3 years post-treatment | 16/328  5 (3–8) | 41/394  10 (8–14) | 6 (4–8) | 11 (8–14) | 7 (4–10) | 11 (8–14) |

CI, confidence interval; HBeAg, hepatitis B e antigen; HBsAg, hepatitis B surface antigen.

GEE, generalized estimating equation
