# Supplementary material for: Final analysis of the international observational S-Collate study of peginterferon alfa-2a in patients with chronic hepatitis B
Source: PLoS One. 2020 Apr 10;15(4):e0230893. doi: 10.1371/journal.pone.0230893 (PMC7147799; doi:10.1371/journal.pone.0230893)
Supplement: S1 Data — (PDF) [file pone.0230893.s008.pdf]

Table 1: Disposition of Patients by HBeAg Status

tdisp01_3000_01 Disposition of Patients by HBeAg Status
Protocol(s): MV22009
Analysis: Modified Intent-to-treat

	HBeAg positive N = 844 No. (%)	HBeAg negative N = 872 No. (%)
Patients enrolled	844 (100)	872 (100)
Patients who received study drug	844 (100)	872 (100)
Patients who received 24 weeks of treatment(a)	748 (89)	817 (94)
Patients who received 48 weeks of treatment(a)	549 (65)	692 (79)
Patients who received 72 weeks of treatment(a)	149 (18)	70 (8)
Patients who received 96 weeks of treatment(a)	90 (11)	31 (4)
Patients who received 120 weeks of treatment(a)	45 (5)	17 (2)
Patients who completed 6 months of follow-up(b)	746 (88)	803 (92)
Patients who completed 1 year of follow-up(b)	670 (79)	744 (85)
Patients who completed 2 years of follow-up(b)	626 (74)	704 (81)
Patients who completed 3 years of follow-up(b)	540 (64)	614 (70)
Patients with ongoing treatment at study end	5 (<1)	2 (<1)

(a) Time from start of treatment to last dose. A patient is considered to have received x weeks of treatment if the time of last dose is on or after the scheduled time of the xth injection.
(b) Time from last dose to last contact. A patient is considered to have completed x months/years of follow-up if the time of last contact is within or after the corresponding time window.

Program : $PROD/cdp10586/i22009b/tdisp01.sas / Output : $PROD/cdp10586/mv22009/reports/tdisp01_3000_01.lst
04SEP2015 17:25 Page 1 of 1

Table 2: Treatment Regimens by Treatment Duration for HBeAg Positive Patients

dm1104tr_3005 Treatment Regimens by Treatment Duration
Protocol(s): MV22009
Analysis: MODIFIED INTENT-TO-TREAT - HBEAG POSITIVE   Center: ALL CENTERS

	48 weeks N = 379	72 weeks N = 44	96 weeks N = 32	Other N = 389
Main treatment regimens				
MONO PEG	324 (85%)	34 (77%)	23 (72%)	316 (81%)
COMBO PEG + NUC	9 (2%)	2 (5%)	3 (9%)	14 (4%)
PEG ADD-ON TO NUC	11 (3%)	3 (7%)	3 (9%)	13 (3%)
OTHER	35 (9%)	5 (11%)	3 (9%)	46 (12%)
n	379	44	32	389
Treatment regimens with Mono Peg subgroups				
PURE MONO PEG	204 (54%)	23 (52%)	15 (47%)	187 (48%)
MONO PEG + POST NUC	104 (27%)	4 (9%)	3 (9%)	108 (28%)
MONO PEG + LATE NUC PARALLEL	16 (4%)	7 (16%)	5 (16%)	21 (5%)
COMBO PEG + NUC	9 (2%)	2 (5%)	3 (9%)	14 (4%)
PEG ADD-ON TO NUC	11 (3%)	3 (7%)	3 (9%)	13 (3%)
OTHER	35 (9%)	5 (11%)	3 (9%)	46 (12%)
n	379	44	32	389

Percentages are based on n (number of valid values). Percentages not calculated if n < 10.
DM11 28AUG2015:17:14:36 (1 of 1)

Table 3: Treatment Regimens by Treatment Duration for HBeAg Negative Patients

dm1104tr_3006 Treatment Regimens by Treatment Duration
Protocol(s): MV22009
Analysis: MODIFIED INTENT-TO-TREAT - HBEAG NEGATIVE   Center: ALL CENTERS

	48 weeks N = 628	72 weeks N = 15	96 weeks N = 12	Other N = 217
Main treatment regimens				
MONO PEG	568 (90%)	12 (80%)	10 (83%)	163 (75%)
COMBO PEG + NUC	23 (4%)	1 (7%)	-	7 (3%)
PEG ADD-ON TO NUC	15 (2%)	1 (7%)	2 (17%)	29 (13%)
OTHER	22 (4%)	1 (7%)	-	18 (8%)
n	628	15	12	217
Treatment regimens with Mono Peg subgroups				
PURE MONO PEG	362 (58%)	12 (80%)	8 (67%)	110 (51%)
MONO PEG + POST NUC	200 (32%)	-	2 (17%)	51 (24%)
MONO PEG + LATE NUC PARALLEL	6 (<1%)	-	-	2 (<1%)
COMBO PEG + NUC	23 (4%)	1 (7%)	-	7 (3%)
PEG ADD-ON TO NUC	15 (2%)	1 (7%)	2 (17%)	29 (13%)
OTHER	22 (4%)	1 (7%)	-	18 (8%)
n	628	15	12	217

Percentages are based on n (number of valid values). Percentages not calculated if n < 10.
DM11 28AUG2015:18:28:50 (1 of 1)

Table 4: Demographic Data by HBeAg Status

dm1101dm_3000 Demographic Data by HBeAg Status
Protocol(s): MV22009
Analysis: MODIFIED INTENT-TO-TREAT   Center: ALL CENTERS

	HBeAg positive N = 844	HBeAg negative N = 872
Sex		
MALE	592 (70%)	644 (74%)
FEMALE	252 (30%)	228 (26%)
n	844	872
Race		
ASIAN/ORIENTAL	640 (80%)	285 (39%)
BLACK	11 (1%)	25 (3%)
CAUCASIAN/WHITE	138 (17%)	419 (57%)
OTHER	7 (<1%)	2 (<1%)
n	796	731
Age in years		
Mean	31.2	38.0
SD	9.33	10.82
SEM	0.32	0.37
Median	29.0	37.0
Min-Max	18 - 71	18 - 74
n	844	872
Weight in kg		
Mean	67.82	73.59
SD	14.002	14.170
SEM	0.486	0.482
Median	65.00	72.00
Min-Max	41.0 - 127.5	42.0 - 135.0
n	830	863
Height in cm		
Mean	170.2	171.3
SD	8.30	8.73
SEM	0.29	0.30
Median	170.0	172.0
Min-Max	150 - 202	147 - 198
n	829	860

n represents number of patients contributing to summary statistics.
Percentages are based on n (number of valid values). Percentages not calculated if n < 10.
DM11 28AUG2015:18:02:37   (1 of 2)

Table 4: Demographic Data by HBeAg Status (cont.)

dm1101dm_3000 Demographic Data by HBeAg Status
Protocol(s): MV22009
Analysis: MODIFIED INTENT-TO-TREAT   Center: ALL CENTERS

	HBeAg positive N = 844	HBeAg negative N = 872
Ethnicity		
HISPANIC	6 (<1%)	10 (1%)
NON-HISPANIC	649 (91%)	585 (85%)
NOT KNOWN	62 (9%)	95 (14%)
n	717	690
Body mass index in kg/sqm		
Mean	23.28	24.98
SD	3.688	3.993
SEM	0.128	0.136
Median	22.77	24.49
Min-Max	15.2 - 40.0	15.4 - 42.6
n	829	860
Body mass index in kg/sqm		
<=20	157 (19%)	70 (8%)
>20-25	437 (53%)	401 (47%)
>25-30	190 (23%)	299 (35%)
>30	45 (5%)	90 (10%)
n	829	860

n represents number of patients contributing to summary statistics.
Percentages are based on n (number of valid values). Percentages not calculated if n < 10.
DM11 28AUG2015:18:02:37 (2 of 2)

Table 5: Baseline Disease Characteristics by HBeAg Status

dm1101dc_3000 Baseline Disease Characteristics by HBeAg Status
Protocol(s): MV22009
Analysis: MODIFIED INTENT-TO-TREAT   Center: ALL CENTERS

	HBeAg positive N = 844	HBeAg negative N = 872
HBV DNA in log10 IU/mL		
Mean	6.25	4.53
SD	1.701	1.721
SEM	0.063	0.063
Median	6.60	4.52
Min-Max	1.1 - 9.7	0.0 - 8.9
n	732	757
HBsAg in IU/mL		
Mean	29701.8	9704.1
SD	75033.59	17840.82
SEM	3987.99	927.50
Median	10662.0	3556.1
Min-Max	21 - 1178253	0 - 180393
n	354	370
HBsAg in log10 IU/mL		
Mean	3.94	3.45
SD	0.768	0.849
SEM	0.041	0.044
Median	4.03	3.55
Min-Max	1.3 - 6.1	0.0 - 5.3
n	354	370
HBsAg (qualitative)		
NEGATIVE	-	1 (<1%)
POSITIVE	781 (100%)	723 (100%)
n	781	724
HBeAg (qualitative)		
NEGATIVE	-	872 (100%)
POSITIVE	844 (100%)	-
n	844	872
Anti-HBs (qualitative)		
NEGATIVE	560 (96%)	404 (96%)
POSITIVE	23 (4%)	15 (4%)
n	583	419

n represents number of patients contributing to summary statistics.
Percentages are based on n (number of valid values). Percentages not calculated if n < 10.
DM11 29SEP2015:11:47:46   (1 of 5)

Table 5: Baseline Disease Characteristics by HBeAg Status (cont.)

dm1101dc_3000 Baseline Disease Characteristics by HBeAg Status
Protocol(s): MV22009
Analysis: MODIFIED INTENT-TO-TREAT   Center: ALL CENTERS

	HBeAg positive N = 844	HBeAg negative N = 872
Anti-HBe (qualitative)		
NEGATIVE	681 (90%)	28 (5%)
POSITIVE	73 (10%)	579 (95%)
n	754	607
Genotype		
A	22 (9%)	35 (16%)
A/G	1 (<1%)	-
B	43 (18%)	23 (10%)
B/C	3 (1%)	1 (<1%)
C	94 (39%)	17 (8%)
D	67 (28%)	116 (53%)
D/F	-	1 (<1%)
D/F or H	-	1 (<1%)
E	11 (5%)	25 (11%)
F	1 (<1%)	1 (<1%)
n	242	220
Genotype (coded)		
A	22 (9%)	35 (16%)
B	43 (18%)	23 (10%)
C	94 (39%)	17 (8%)
D	67 (28%)	116 (53%)
OTHER	16 (7%)	29 (13%)
n	242	220
Mode of infection		
PERINATAL	236 (71%)	141 (47%)
SEXUAL	30 (9%)	36 (12%)
TRANSFUSION	16 (5%)	28 (9%)
OCCUPATIONAL	6 (2%)	12 (4%)
INJECTION DRUG USE	3 (<1%)	8 (3%)
OTHER	41 (12%)	72 (24%)
n	332	297

n represents number of patients contributing to summary statistics.
Percentages are based on n (number of valid values). Percentages not calculated if n < 10.
DM11 29SEP2015:11:47:46   (2 of 5)

Table 5: Baseline Disease Characteristics by HBeAg Status (cont.)

dm1101dc_3000 Baseline Disease Characteristics by HBeAg Status
Protocol(s): MV22009
Analysis: MODIFIED INTENT-TO-TREAT   Center: ALL CENTERS

	HBeAg positive N = 844	HBeAg negative N = 872
Duration of infection in years		
Mean	15.3	15.4
SD	12.20	13.63
SEM	0.43	0.49
Median	13.0	11.0
Min-Max	0 - 71	0 - 62
n	793	766
Regular alcohol consumption?		
NO	768 (91%)	805 (93%)
YES	72 (9%)	65 (7%)
n	840	870
Weekly alcohol consumption in units/drinks		
Mean	30.88	10.88
SD	113.063	12.924
SEM	12.486	1.463
Median	7.00	7.00
Min-Max	0.0 - 1000.0	0.0 - 64.0
n	82	78
Previous therapy with interferon		
NO/UNKNOWN	773 (92%)	797 (91%)
YES	71 (8%)	75 (9%)
n	844	872
Previous therapy with antiviral agents		
NO/UNKNOWN	838 (99%)	868 (100%)
YES	6 (<1%)	4 (<1%)
n	844	872
Previous therapy with NAs		
NO/UNKNOWN	688 (82%)	741 (85%)
YES	156 (18%)	131 (15%)
n	844	872
Co-infection with HDV		
NO	361 (98%)	457 (94%)
YES	8 (2%)	28 (6%)
n	369	485

n represents number of patients contributing to summary statistics.
Percentages are based on n (number of valid values). Percentages not calculated if n < 10.
DM11 29SEP2015:11:47:46   (3 of 5)

Table 5: Baseline Disease Characteristics by HBeAg Status (cont.)

dm1101dc_3000 Baseline Disease Characteristics by HBeAg Status
Protocol(s): MV22009
Analysis: MODIFIED INTENT-TO-TREAT Center: ALL CENTERS

	HBeAg positive N = 844	HBeAg negative N = 872
HBV vaccination prior to therapy		
NO	825 (98%)	836 (96%)
YES	18 (2%)	33 (4%)
n	843	869
Method to assess liver fibrosis		
BIOPSY (INVASIVE)	193 (23%)	349 (40%)
NON-INVASIVE	163 (19%)	183 (21%)
NOT ASSESSED	488 (58%)	340 (39%)
n	844	872
Result of liver fibrosis assessment		
NO CIRRHOSIS	305 (86%)	455 (86%)
TRANSITION TO CIRRHOSIS	37 (10%)	50 (9%)
CIRRHOSIS	14 (4%)	27 (5%)
n	356	532
Fibroscan value in kPA		
Mean	9.69	7.55
SD	6.920	3.504
SEM	0.925	0.360
Median	7.80	6.70
Min-Max	1.5 - 37.4	1.0 - 23.9
n	56	95
Scoring of Scheuer system		
0	4 (8%)	10 (12%)
1	25 (51%)	39 (48%)
2	17 (35%)	23 (28%)
3	3 (6%)	7 (9%)
4	-	2 (2%)
n	49	81

n represents number of patients contributing to summary statistics.
Percentages are based on n (number of valid values). Percentages not calculated if n < 10.
DM11 29SEP2015:11:47:46 (4 of 5)

dm1101dc_3000 Baseline Disease Characteristics by HBeAg Status
Protocol(s): MV22009
Analysis: MODIFIED INTENT-TO-TREAT   Center: ALL CENTERS

	HBeAg positive N = 844	HBeAg negative N = 872
Scoring of METAVIR/Batts Ludwig		
0	4 (5%)	15 (9%)
1	31 (41%)	53 (30%)
2	28 (37%)	83 (48%)
3	10 (13%)	15 (9%)
4	2 (3%)	8 (5%)
n	75	174
Scoring of Knodell		
0	13 (26%)	19 (24%)
1	29 (58%)	48 (62%)
3	8 (16%)	9 (12%)
4	-	2 (3%)
n	50	78
Scoring of Ishak		
0	6 (6%)	11 (11%)
1	30 (32%)	26 (27%)
2	24 (26%)	32 (33%)
3	18 (19%)	11 (11%)
4	10 (11%)	10 (10%)
5	3 (3%)	3 (3%)
6	2 (2%)	3 (3%)
n	93	96
ALT in IU/L		
Mean	178.6	117.6
SD	152.40	111.42
SEM	5.28	3.83
Median	133.0	79.3
Min-Max	11 - 1218	9 - 802
n	834	846
ALT ratio		
Mean	3.25	2.14
SD	2.771	2.026
SEM	0.096	0.070
Median	2.42	1.44
Min-Max	0.2 - 22.1	0.2 - 14.6
n	834	846

n represents number of patients contributing to summary statistics.
Percentages are based on n (number of valid values). Percentages not calculated if n < 10.
DM11 29SEP2015:11:47:46 (5 of 5)

Table 6: Response Rate of HBsAg Clearance 3 Years Post-Treatment by HBeAg Status

tep01_3000_PE_01 Response Rate of HBsAg Clearance 3 Years Post-Treatment by HBeAg Status
Protocol(s): MV22009
Analysis: Modified Intent-to-treat

		HBeAg positive N=844			HBeAg negative N=872		
		n1/ n2	%	(95%-CI)	n1/ n2	%	(95%-CI)
3 years post-treatment							
HBsAg clearance	(a)	16/ 844	2	(1, 3)	41/ 872	5	(3, 6)
	(b)	16/ 328	5	(3, 8)	41/ 394	10	(8, 14)

n1: Number of patients with response in the analysis group. n2: See (a) and (b).
95%-CI: Exact confidence limits for the binomial proportion.
(a) Analysis A: Response in relation to all patients in the analysis group (n2).
(b) Analysis B: Response in relation to all patients in the analysis group with measurement (n2).

Program : $PROD/cdp10586/i22009b/tep01.sas / Output : $PROD/cdp10586/mv22009/reports/tep01_3000_PE_01.lst
04SEP2015 17:27 Page 1 of 1

Table 7: Exposure to PEG IFN by HBeAg Status

dm1101mt_9000 Exposure to Pegasys by HBeAg Status
Protocol(s): MV22009 (I22009B)
Analysis: SAFETY (48)   Center: ALL CENTERS

	E-positive N = 653	E-negative N = 783	Unknown N = 39	Total N = 1475
**Initial dose in ug**				
90	-	-	1 (3%)	1 (<1%)
135	35 (5%)	15 (2%)	2 (5%)	52 (4%)
180	618 (95%)	768 (98%)	36 (92%)	1422 (96%)
n	653	783	39	1475
**Treatment duration in days**				
Mean	262.4	302.5	256.5	283.5
SD	99.59	73.70	111.38	89.42
SEM	3.90	2.63	17.84	2.33
Median	330.0	330.0	330.0	330.0
Min-Max	1 - 377	1 - 377	17 - 358	1 - 377
n	653	783	39	1475
**Treatment duration in weeks**				
(1) 3 (>0-6)	25 (4%)	10 (1%)	3 (8%)	38 (3%)
(2) 12 (>6-18)	50 (8%)	32 (4%)	4 (10%)	86 (6%)
(3) 24 (>18-30)	140 (21%)	71 (9%)	5 (13%)	216 (15%)
(4) 36 (>30-42)	47 (7%)	33 (4%)	4 (10%)	84 (6%)
(5) 48 (>42-54)	391 (60%)	637 (81%)	23 (59%)	1051 (71%)
n	653	783	39	1475
**Cumulative dose in ug**				
Mean	6707.2	7754.0	6558.5	7258.9
SD	2554.67	1965.41	2868.07	2329.43
SEM	99.97	70.24	459.26	60.65
Median	8460.0	8640.0	8460.0	8640.0
Min-Max	180 - 9720	180 - 9720	540 - 9360	180 - 9720
n	653	783	39	1475

n represents number of patients contributing to summary statistics.
Percentages are based on n (number of valid values). Percentages not calculated if n < 10.
Patients who had treatment interrupted and then resumed treatment are included as having received treatment for the full period of time.
Cumulative dose is estimated (injections were not recorded individually).
DM11 19APR2013:15:29:57   (1 of 1)

Table 8: Response Rates over Time by HBeAg Status: HBsAg and HBV DNA Related Endpoints

tep01_3000_01_01 Response Rates over Time by HBeAg Status: HBsAg and HBV DNA Related Endpoints
Protocol(s): MV22009
Analysis: Modified Intent-to-treat

		HBeAg positive N=844			HBeAg negative N=872		
		n1/ n2	%	(95%-CI)	n1/ n2	%	(95%-CI)
End of treatment							
HBsAg <1000 IU/mL	(a)	114/ 844	14	(11, 16)	163/ 872	19	(16, 21)
	(b)	114/ 347	33	(28, 38)	163/ 378	43	(38, 48)
HBsAg <100 IU/mL	(a)	63/ 844	7	(6, 9)	83/ 872	10	(8, 12)
	(b)	63/ 376	17	(13, 21)	83/ 384	22	(18, 26)
HBsAg <10 IU/mL	(a)	34/ 844	4	(3, 6)	44/ 872	5	(4, 7)
	(b)	34/ 376	9	(6, 12)	44/ 384	11	(8, 15)
HBsAg clearance	(a)	23/ 844	3	(2, 4)	30/ 872	3	(2, 5)
	(b)	23/ 570	4	(3, 6)	30/ 641	5	(3, 7)
HBsAg seroconversion	(a)	16/ 844	2	(1, 3)	13/ 872	1	(1, 3)
	(b)	16/ 338	5	(3, 8)	13/ 286	5	(2, 8)
HBV DNA <2000 IU/mL	(a)	332/ 844	39	(36, 43)	612/ 872	70	(67, 73)
	(b)	332/ 616	54	(50, 58)	612/ 689	89	(86, 91)

n1: Number of patients with response in the analysis group. n2: See (a) and (b).
95%-CI: Exact confidence limits for the binomial proportion.
(a) Analysis A: Response in relation to all patients in the analysis group (n2).
(b) Analysis B: Response in relation to all patients in the analysis group with measurement (n2).
Endpoints incl. HBV DNA post-treatment: Patients receiving NUCs at this time point are considered non-responders.

Program : $PROD/cdp10586/i22009b/tep01.sas / Output : $PROD/cdp10586/mv22009/reports/tep01_3000_01_01.lst
04SEP2015 17:32 Page 1 of 5

Table 8: Response Rates over Time by HBeAg Status: HBsAg and HBV DNA Related Endpoints (cont.)

tep01_3000_01_01 Response Rates over Time by HBeAg Status: HBsAg and HBV DNA Related Endpoints
Protocol(s): MV22009
Analysis: Modified Intent-to-treat

		HBeAg positive N=844			HBeAg negative N=872		
		n1/ n2	%	(95%-CI)	n1/ n2	%	(95%-CI)
6 months post-treatment							
HBsAg <1000 IU/mL	(a)	94/ 844	11	(9, 13)	131/ 872	15	(13, 18)
	(b)	94/ 280	34	(28, 39)	131/ 319	41	(36, 47)
HBsAg <100 IU/mL	(a)	42/ 844	5	(4, 7)	57/ 872	7	(5, 8)
	(b)	42/ 302	14	(10, 18)	57/ 327	17	(13, 22)
HBsAg <10 IU/mL	(a)	23/ 844	3	(2, 4)	30/ 872	3	(2, 5)
	(b)	23/ 302	8	(5, 11)	30/ 327	9	(6, 13)
HBsAg clearance	(a)	27/ 844	3	(2, 5)	37/ 872	4	(3, 6)
	(b)	27/ 501	5	(4, 8)	37/ 540	7	(5, 9)
HBsAg seroconversion	(a)	15/ 844	2	(1, 3)	24/ 872	3	(2, 4)
	(b)	15/ 318	5	(3, 8)	24/ 254	9	(6, 14)
HBV DNA <2000 IU/mL	(a)	201/ 844	24	(21, 27)	287/ 872	33	(30, 36)
	(b)	201/ 584	34	(31, 38)	287/ 634	45	(41, 49)

n1: Number of patients with response in the analysis group. n2: See (a) and (b).
95%-CI: Exact confidence limits for the binomial proportion.
(a) Analysis A: Response in relation to all patients in the analysis group (n2).
(b) Analysis B: Response in relation to all patients in the analysis group with measurement (n2).
Endpoints incl. HBV DNA post-treatment: Patients receiving NUCs at this time point are considered non-responders.

Program : $PROD/cdp10586/i22009b/tep01.sas / Output : $PROD/cdp10586/mv22009/reports/tep01_3000_01_01.lst
04SEP2015 17:32 Page 2 of 5

Table 8: Response Rates over Time by HBeAg Status: HBsAg and HBV DNA Related Endpoints (cont.)

tep01_3000_01_01 Response Rates over Time by HBeAg Status: HBsAg and HBV DNA Related Endpoints
Protocol(s): MV22009
Analysis: Modified Intent-to-treat

		HBeAg positive N=844			HBeAg negative N=872		
		n1/ n2	%	(95%-CI)	n1/ n2	%	(95%-CI)
1 year post-treatment							
HBsAg <1000 IU/mL	(a)	56/ 844	7	(5, 9)	113/ 872	13	(11, 15)
	(b)	56/ 208	27	(21, 33)	113/ 250	45	(39, 52)
HBsAg <100 IU/mL	(a)	28/ 844	3	(2, 5)	56/ 872	6	(5, 8)
	(b)	28/ 228	12	(8, 17)	56/ 256	22	(17, 27)
HBsAg <10 IU/mL	(a)	20/ 844	2	(1, 4)	34/ 872	4	(3, 5)
	(b)	20/ 228	9	(5, 13)	34/ 256	13	(9, 18)
HBsAg clearance	(a)	25/ 844	3	(2, 4)	39/ 872	4	(3, 6)
	(b)	25/ 431	6	(4, 8)	39/ 486	8	(6, 11)
HBsAg seroconversion	(a)	18/ 844	2	(1, 3)	18/ 872	2	(1, 3)
	(b)	18/ 285	6	(4, 10)	18/ 239	8	(5, 12)
HBV DNA <2000 IU/mL	(a)	170/ 844	20	(17, 23)	199/ 872	23	(20, 26)
	(b)	170/ 525	32	(28, 37)	199/ 557	36	(32, 40)

n1: Number of patients with response in the analysis group. n2: See (a) and (b).
95%-CI: Exact confidence limits for the binomial proportion.
(a) Analysis A: Response in relation to all patients in the analysis group (n2).
(b) Analysis B: Response in relation to all patients in the analysis group with measurement (n2).
Endpoints incl. HBV DNA post-treatment: Patients receiving NUCs at this time point are considered non-responders.

Program : $PROD/cdp10586/i22009b/tep01.sas / Output : $PROD/cdp10586/mv22009/reports/tep01_3000_01_01.lst
04SEP2015 17:32 Page 3 of 5

Table 8: Response Rates over Time by HBeAg Status: HBsAg and HBV DNA Related Endpoints (cont.)

tep01_3000_01_01 Response Rates over Time by HBeAg Status: HBsAg and HBV DNA Related Endpoints
Protocol(s): MV22009
Analysis: Modified Intent-to-treat

		HBeAg positive N=844			HBeAg negative N=872		
		n1/ n2	%	(95%-CI)	n1/ n2	%	(95%-CI)
2 years post-treatment							
HBsAg <1000 IU/mL	(a)	68/ 844	8	(6, 10)	87/ 872	10	(8, 12)
	(b)	68/ 223	30	(25, 37)	87/ 209	42	(35, 49)
HBsAg <100 IU/mL	(a)	31/ 844	4	(3, 5)	39/ 872	4	(3, 6)
	(b)	31/ 233	13	(9, 18)	39/ 213	18	(13, 24)
HBsAg <10 IU/mL	(a)	24/ 844	3	(2, 4)	27/ 872	3	(2, 4)
	(b)	24/ 233	10	(7, 15)	27/ 213	13	(9, 18)
HBsAg clearance	(a)	21/ 844	2	(2, 4)	32/ 872	4	(3, 5)
	(b)	21/ 414	5	(3, 8)	32/ 440	7	(5, 10)
HBsAg seroconversion	(a)	14/ 844	2	(1, 3)	11/ 872	1	(1, 2)
	(b)	14/ 257	5	(3, 9)	11/ 217	5	(3, 9)
HBV DNA <2000 IU/mL	(a)	150/ 844	18	(15, 21)	162/ 872	19	(16, 21)
	(b)	150/ 505	30	(26, 34)	162/ 534	30	(26, 34)

n1: Number of patients with response in the analysis group. n2: See (a) and (b).
95%-CI: Exact confidence limits for the binomial proportion.
(a) Analysis A: Response in relation to all patients in the analysis group (n2).
(b) Analysis B: Response in relation to all patients in the analysis group with measurement (n2).
Endpoints incl. HBV DNA post-treatment: Patients receiving NUCs at this time point are considered non-responders.

Program : $PROD/cdp10586/i22009b/tep01.sas / Output : $PROD/cdp10586/mv22009/reports/tep01_3000_01_01.lst
04SEP2015 17:32 Page 4 of 5

Table 8: Response Rates over Time by HBeAg Status: HBsAg and HBV DNA Related Endpoints (cont.)

tep01_3000_01_01 Response Rates over Time by HBeAg Status: HBsAg and HBV DNA Related Endpoints
Protocol(s): MV22009
Analysis: Modified Intent-to-treat

		HBeAg positive N=844			HBeAg negative N=872		
		n1/ n2	%	(95%-CI)	n1/ n2	%	(95%-CI)
3 years post-treatment							
HBsAg <1000 IU/mL	(a)	55/ 844	7	(5, 8)	97/ 872	11	(9, 13)
	(b)	55/ 173	32	(25, 39)	97/ 188	52	(44, 59)
HBsAg <100 IU/mL	(a)	24/ 844	3	(2, 4)	45/ 872	5	(4, 7)
	(b)	24/ 180	13	(9, 19)	45/ 191	24	(18, 30)
HBsAg <10 IU/mL	(a)	15/ 844	2	(1, 3)	29/ 872	3	(2, 5)
	(b)	15/ 180	8	(5, 13)	29/ 191	15	(10, 21)
HBsAg clearance	(a)	16/ 844	2	(1, 3)	41/ 872	5	(3, 6)
	(b)	16/ 328	5	(3, 8)	41/ 394	10	(8, 14)
HBsAg seroconversion	(a)	9/ 844	1	(0, 2)	16/ 872	2	(1, 3)
	(b)	9/ 193	5	(2, 9)	16/ 179	9	(5, 14)
HBV DNA <2000 IU/mL	(a)	113/ 844	13	(11, 16)	139/ 872	16	(14, 19)
	(b)	113/ 421	27	(23, 31)	139/ 448	31	(27, 36)

n1: Number of patients with response in the analysis group. n2: See (a) and (b).
95%-CI: Exact confidence limits for the binomial proportion.
(a) Analysis A: Response in relation to all patients in the analysis group (n2).
(b) Analysis B: Response in relation to all patients in the analysis group with measurement (n2).
Endpoints incl. HBV DNA post-treatment: Patients receiving NUCs at this time point are considered non-responders.

Program : $PROD/cdp10586/i22009b/tep01.sas / Output : $PROD/cdp10586/mv22009/reports/tep01_3000_01_01.lst
04SEP2015 17:32 Page 5 of 5

Table 9: Response Rates over Time by HBeAg Status: HBeAg Related Endpoints

tep01_3005_03_01 Response Rates over Time by HBeAg Status: HBeAg Related Endpoints
Protocol(s): MV22009
Analysis: Modified Intent-to-treat - HBeAg Positive

		HBeAg positive N=844 n1/ n2 % (95%-CI)
End of treatment		
HBeAg loss	(a)	149/ 844 18 (15, 20)
	(b)	149/ 554 27 (23, 31)
HBeAg seroconversion	(a)	116/ 844 14 (11, 16)
	(b)	116/ 509 23 (19, 27)
HBeAg seroconversion and HBV DNA <2000 IU/mL	(a)	97/ 844 11 (9, 14)
	(b)	97/ 480 20 (17, 24)

n1: Number of patients with response in the analysis group. n2: See (a) and (b).
95%-CI: Exact confidence limits for the binomial proportion.
(a) Analysis A: Response in relation to all patients in the analysis group (n2).
(b) Analysis B: Response in relation to all patients in the analysis group with measurement (n2).
Endpoints incl. HBV DNA post-treatment: Patients receiving NUCs at this time point are considered non-responders.

Program : $PROD/cdp10586/i22009b/tep01.sas / Output : $PROD/cdp10586/mv22009/reports/tep01_3005_03_01.lst
04SEP2015 17:33 Page 1 of 5

Table 9: Response Rates over Time by HBeAg Status: HBeAg Related Endpoints

tep01_3005_03_01 Response Rates over Time by HBeAg Status: HBeAg Related Endpoints
Protocol(s): MV22009
Analysis: Modified Intent-to-treat - HBeAg Positive

		HBeAg positive N=844 n1/ n2 % (95%-CI)
6 months post-treatment		
HBeAg loss	(a)	180/ 844 21 (19, 24)
	(b)	180/ 516 35 (31, 39)
HBeAg seroconversion	(a)	135/ 844 16 (14, 19)
	(b)	135/ 486 28 (24, 32)
HBeAg seroconversion and HBV DNA <2000 IU/mL	(a)	80/ 844 9 (8, 12)
	(b)	80/ 461 17 (14, 21)

n1: Number of patients with response in the analysis group. n2: See (a) and (b).
95%-CI: Exact confidence limits for the binomial proportion.
(a) Analysis A: Response in relation to all patients in the analysis group (n2).
(b) Analysis B: Response in relation to all patients in the analysis group with measurement (n2).
Endpoints incl. HBV DNA post-treatment: Patients receiving NUCs at this time point are considered non-responders.

Program : $PROD/cdp10586/i22009b/tep01.sas / Output : $PROD/cdp10586/mv22009/reports/tep01_3005_03_01.lst
04SEP2015 17:33 Page 2 of 5

Table 9: Response Rates over Time by HBeAg Status: HBeAg Related Endpoints

tep01_3005_03_01 Response Rates over Time by HBeAg Status: HBeAg Related Endpoints
Protocol(s): MV22009
Analysis: Modified Intent-to-treat - HBeAg Positive

		HBeAg positive N=844 n1/ n2 % (95%-CI)
1 year post-treatment		
HBeAg loss	(a)	182/ 844 22 (19, 24)
	(b)	182/ 460 40 (35, 44)
HBeAg seroconversion	(a)	140/ 844 17 (14, 19)
	(b)	140/ 438 32 (28, 37)
HBeAg seroconversion and HBV DNA <2000 IU/mL	(a)	60/ 844 7 (5, 9)
	(b)	60/ 415 14 (11, 18)

n1: Number of patients with response in the analysis group. n2: See (a) and (b).
95%-CI: Exact confidence limits for the binomial proportion.
(a) Analysis A: Response in relation to all patients in the analysis group (n2).
(b) Analysis B: Response in relation to all patients in the analysis group with measurement (n2).
Endpoints incl. HBV DNA post-treatment: Patients receiving NUCs at this time point are considered non-responders.

Program : $PROD/cdp10586/i22009b/tep01.sas / Output : $PROD/cdp10586/mv22009/reports/tep01_3005_03_01.lst
04SEP2015 17:33 Page 3 of 5

Table 9: Response Rates over Time by HBeAg Status: HBeAg Related Endpoints

tep01_3005_03_01 Response Rates over Time by HBeAg Status: HBeAg Related Endpoints
Protocol(s): MV22009
Analysis: Modified Intent-to-treat - HBeAg Positive

		HBeAg positive N=844 n1/ n2 % (95%-CI)
2 years post-treatment		
HBeAg loss	(a)	198/ 844 23 (21, 26)
	(b)	198/ 430 46 (41, 51)
HBeAg seroconversion	(a)	154/ 844 18 (16, 21)
	(b)	154/ 398 39 (34, 44)
HBeAg seroconversion and HBV DNA <2000 IU/mL	(a)	60/ 844 7 (5, 9)
	(b)	60/ 383 16 (12, 20)

n1: Number of patients with response in the analysis group. n2: See (a) and (b).
95%-CI: Exact confidence limits for the binomial proportion.
(a) Analysis A: Response in relation to all patients in the analysis group (n2).
(b) Analysis B: Response in relation to all patients in the analysis group with measurement (n2).
Endpoints incl. HBV DNA post-treatment: Patients receiving NUCs at this time point are considered non-responders.

Program : $PROD/cdp10586/i22009b/tep01.sas / Output : $PROD/cdp10586/mv22009/reports/tep01_3005_03_01.lst
04SEP2015 17:33 Page 4 of 5

Table 9: Response Rates over Time by HBeAg Status: HBeAg Related Endpoints

tep01_3005_03_01 Response Rates over Time by HBeAg Status: HBeAg Related Endpoints
Protocol(s): MV22009
Analysis: Modified Intent-to-treat - HBeAg Positive

		HBeAg positive N=844 n1/ n2 % (95%-CI)
3 years post-treatment		
HBeAg loss	(a)	163/ 844 19 (17, 22)
	(b)	163/ 331 49 (44, 55)
HBeAg seroconversion	(a)	115/ 844 14 (11, 16)
	(b)	115/ 304 38 (32, 44)
HBeAg seroconversion and HBV DNA <2000 IU/mL	(a)	44/ 844 5 (4, 7)
	(b)	44/ 285 15 (11, 20)

n1: Number of patients with response in the analysis group. n2: See (a) and (b).
95%-CI: Exact confidence limits for the binomial proportion.
(a) Analysis A: Response in relation to all patients in the analysis group (n2).
(b) Analysis B: Response in relation to all patients in the analysis group with measurement (n2).
Endpoints incl. HBV DNA post-treatment: Patients receiving NUCs at this time point are considered non-responders.

Program : $PROD/cdp10586/i22009b/tep01.sas / Output : $PROD/cdp10586/mv22009/reports/tep01_3005_03_01.lst
04SEP2015 17:33 Page 5 of 5

Table 10: Normalization of ALT over Time by HBeAg Status

tep01_3007_02_01 Normalization of ALT over Time by HBeAg Status
Protocol(s): MV22009
Analysis: Modified Intent-to-treat - Baseline ALT >ULN

		HBeAg positive N=751			HBeAg negative N=610		
		n1/ n2	%	(95%-CI)	n1/ n2	%	(95%-CI)
Week 12							
Normalization of ALT	(a)	174/ 751	23	(20, 26)	139/ 610	23	(20, 26)
	(b)	174/ 685	25	(22, 29)	139/ 564	25	(21, 28)
Week 24							
Normalization of ALT	(a)	210/ 751	28	(25, 31)	167/ 610	27	(24, 31)
	(b)	210/ 624	34	(30, 38)	167/ 529	32	(28, 36)
Week 36							
Normalization of ALT	(a)	191/ 751	25	(22, 29)	185/ 610	30	(27, 34)
	(b)	191/ 482	40	(35, 44)	185/ 461	40	(36, 45)
Week 48							
Normalization of ALT	(a)	202/ 751	27	(24, 30)	213/ 610	35	(31, 39)
	(b)	202/ 454	44	(40, 49)	213/ 433	49	(44, 54)
End of treatment							
Normalization of ALT	(a)	258/ 751	34	(31, 38)	227/ 610	37	(33, 41)
	(b)	258/ 571	45	(41, 49)	227/ 496	46	(41, 50)

n1: Number of patients with response in the analysis group. n2: See (a) and (b).
95%-CI: Exact confidence limits for the binomial proportion.
(a) Analysis A: Response in relation to all patients in the analysis group (n2).
(b) Analysis B: Response in relation to all patients in the analysis group with measurement (n2).

Program : $PROD/cdp10586/i22009b/tep01.sas / Output : $PROD/cdp10586/mv22009/reports/tep01_3007_02_01.lst
04SEP2015 17:32 Page 1 of 2

Table 10: Normalization of ALT over Time by HBeAg Status (cont.)

tep01_3007_02_01 Normalization of ALT over Time by HBeAg Status
Protocol(s): MV22009
Analysis: Modified Intent-to-treat - Baseline ALT >ULN

		HBeAg positive N=751		HBeAg negative N=610	
		n1/ n2	% (95%-CI)	n1/ n2	% (95%-CI)
6 months post-treatment					
Normalization of ALT	(a)	337/ 751	45 (41, 49)	309/ 610	51 (47, 55)
	(b)	337/ 542	62 (58, 66)	309/ 475	65 (61, 69)
1 year post-treatment					
Normalization of ALT	(a)	367/ 751	49 (45, 53)	314/ 610	51 (47, 56)
	(b)	367/ 492	75 (71, 78)	314/ 432	73 (68, 77)
2 years post-treatment					
Normalization of ALT	(a)	363/ 751	48 (45, 52)	324/ 610	53 (49, 57)
	(b)	363/ 461	79 (75, 82)	324/ 419	77 (73, 81)
3 years post-treatment					
Normalization of ALT	(a)	324/ 751	43 (40, 47)	301/ 610	49 (45, 53)
	(b)	324/ 405	80 (76, 84)	301/ 366	82 (78, 86)

n1: Number of patients with response in the analysis group. n2: See (a) and (b).
95%-CI: Exact confidence limits for the binomial proportion.
(a) Analysis A: Response in relation to all patients in the analysis group (n2).
(b) Analysis B: Response in relation to all patients in the analysis group with measurement (n2).

Program : $PROD/cdp10586/i22009b/tep01.sas / Output : $PROD/cdp10586/mv22009/reports/tep01_3007_02_01.lst
04SEP2015 17:32 Page 2 of 2

Table 11: Response Rates over Time by HBeAg Status: HBV DNA <2,000 IU/mL and Normalization of ALT

tep01_3007_05_01 Response Rates over Time by HBeAg Status: HBV DNA <2000 IU/mL and Normalization of ALT
Protocol(s): MV22009
Analysis: Modified Intent-to-treat - Baseline ALT >ULN

		HBeAg positive N=751		HBeAg negative N=610	
		n1/ n2	% (95%-CI)	n1/ n2	% (95%-CI)
**End of treatment**					
HBV DNA <2000 IU/mL and normalization of ALT	(a)	159/ 751	21 (18, 24)	187/ 610	31 (27, 34)
	(b)	159/ 515	31 (27, 35)	187/ 451	41 (37, 46)
**6 months post-treatment**					
HBV DNA <2000 IU/mL and normalization of ALT	(a)	140/ 751	19 (16, 22)	149/ 610	24 (21, 28)
	(b)	140/ 498	28 (24, 32)	149/ 426	35 (30, 40)
**1 year post-treatment**					
HBV DNA <2000 IU/mL and normalization of ALT	(a)	126/ 751	17 (14, 20)	108/ 610	18 (15, 21)
	(b)	126/ 456	28 (24, 32)	108/ 372	29 (24, 34)
**2 years post-treatment**					
HBV DNA <2000 IU/mL and normalization of ALT	(a)	100/ 751	13 (11, 16)	89/ 610	15 (12, 18)
	(b)	100/ 431	23 (19, 27)	89/ 364	24 (20, 29)
**3 years post-treatment**					
HBV DNA <2000 IU/mL and normalization of ALT	(a)	82/ 751	11 (9, 13)	83/ 610	14 (11, 17)
	(b)	82/ 369	22 (18, 27)	83/ 300	28 (23, 33)

n1: Number of patients with response in the analysis group. n2: See (a) and (b).
95%-CI: Exact confidence limits for the binomial proportion.
(a) Analysis A: Response in relation to all patients in the analysis group (n2).
(b) Analysis B: Response in relation to all patients in the analysis group with measurement (n2).
Endpoints incl. HBV DNA post-treatment: Patients receiving NUCs at this time point are considered non-responders.

Program : $PROD/cdp10586/i22009b/tep01.sas / Output : $PROD/cdp10586/mv22009/reports/tep01_3007_05_01.lst
04SEP2015 17:33 Page 1 of 1

Table 12: Exposure to PEG IFN by HBeAg Status

dm1101mt_5000 Exposure to PEG IFN by HBeAg Status
Protocol(s): MV22009
Analysis: SAFETY   Center: ALL CENTERS

	HBeAg positive N = 863	HBeAg negative N = 890	Unknown N = 48	Total N = 1801
**Initial dose in ug**				
90	-	-	1 (2%)	1 (<1%)
135	88 (10%)	29 (3%)	3 (6%)	120 (7%)
180	775 (90%)	861 (97%)	44 (92%)	1680 (93%)
n	863	890	48	1801
**Treatment duration in days**				
Mean	366.8	341.0	340.5	353.3
SD	245.01	156.00	245.49	206.15
SEM	8.36	5.24	35.43	4.87
Median	330.0	330.0	331.5	330.0
Min-Max	1 - 1578	1 - 1653	17 - 1478	1 - 1653
n	858	886	48	1792
**Treatment duration in weeks**				
(1) 3 (>0-6)	24 (3%)	10 (1%)	3 (6%)	37 (2%)
(2) 12 (>6-18)	49 (6%)	32 (4%)	4 (8%)	85 (5%)
(3) 24 (>18-30)	141 (16%)	71 (8%)	5 (10%)	217 (12%)
(4) 36 (>30-42)	47 (5%)	33 (4%)	4 (8%)	84 (5%)
(5) 48 (>42-54)	386 (45%)	637 (72%)	23 (48%)	1046 (58%)
(6) 60 (>54-66)	41 (5%)	29 (3%)	2 (4%)	72 (4%)
(7) 72 (>66-78)	44 (5%)	15 (2%)	2 (4%)	61 (3%)
(8) 84 (>78-90)	26 (3%)	22 (2%)	-	48 (3%)
(9) 96 (>90-102)	32 (4%)	12 (1%)	2 (4%)	46 (3%)
(10) 108 (>102-114)	17 (2%)	6 (<1%)	1 (2%)	24 (1%)
(11) 120 (>114-126)	13 (2%)	3 (<1%)	-	16 (<1%)
(12) >=132 (>126)	38 (4%)	16 (2%)	2 (4%)	56 (3%)
n	858	886	48	1792
**Cumulative dose in ug**				
Mean	9093.2	8620.9	8500.3	8843.8
SD	5667.42	3601.86	6071.07	4775.43
SEM	193.48	121.01	876.28	112.81
Median	8640.0	8640.0	8640.0	8640.0
Min-Max	180 - 38700	180 - 33300	540 - 38160	180 - 38700
n	858	886	48	1792

n represents number of patients contributing to summary statistics.
Percentages are based on n (number of valid values). Percentages not calculated if n < 10.
Patients who had treatment interrupted and then resumed treatment are included as having received treatment for the full period of time.
Cumulative dose is estimated (injections were not recorded individually).
DM11 28AUG2015:18:26:56 (1 of 1)

Table 13: Adverse Events with an Incidence Rate of at least 5% by HBeAg Status

ae1301a1_5000 Most Frequent Adverse Events with an Incidence Rate of at Least 5% by HBeAg Status
Use of Cutoff Point of 4.5%
Protocol(s): MV22009
Analysis: SAFETY   Center: ALL CENTERS

Adverse Event	HBeAg positive N = 863 No. (%)	HBeAg negative N = 890 No. (%)	Unknown N = 48 No. (%)	Total N = 1801 No. (%)
THROMBOCYTOPENIA	54 (6)	185 (21)	9 (19)	248 (14)
PYREXIA	147 (17)	92 (10)	2 (4)	241 (13)
NEUTROPENIA	76 (9)	153 (17)	5 (10)	234 (13)
HEADACHE	84 (10)	121 (14)	7 (15)	212 (12)
ASTHENIA	64 (7)	124 (14)	7 (15)	195 (11)
LEUKOPENIA	56 (6)	124 (14)	6 (13)	186 (10)
FATIGUE	89 (10)	93 (10)	3 (6)	185 (10)
ALOPECIA	113 (13)	67 (8)	1 (2)	181 (10)
INFLUENZA LIKE ILLNESS	69 (8)	92 (10)	4 (8)	165 (9)
MYALGIA	81 (9)	74 (8)	2 (4)	157 (9)
WEIGHT DECREASED	58 (7)	76 (9)	7 (15)	141 (8)
PRURITUS	41 (5)	51 (6)	4 (8)	96 (5)
WHITE BLOOD CELL COUNT DECREASED	73 (8)	18 (2)	1 (2)	92 (5)
DECREASED APPETITE	48 (6)	35 (4)	2 (4)	85 (5)
DEPRESSION	23 (3)	44 (5)	2 (4)	69 (4)
NAUSEA	33 (4)	28 (3)	4 (8)	65 (4)
ALANINE AMINOTRANSFERASE INCREASED	39 (5)	23 (3)	1 (2)	63 (3)
ARTHRALGIA	20 (2)	41 (5)	2 (4)	63 (3)
ANAEMIA	11 (1)	49 (6)	1 (2)	61 (3)
GAMMA-GLUTAMYLTRANSFERASE INCREASED	3 (<1)	3 (<1)	3 (6)	9 (<1)

Investigator text for Adverse Events encoded using MedDRA version 17.1.
Percentages are based on N.
Multiple occurrences of the same adverse event in one individual counted only once.
AE13 28AUG2015:17:11:02 (1 of 1)

Table 14: Adverse Events and Laboratory Abnormalities Leading to Dose Modification of Treatment with PEG IFN by HBeAg Status

tmedt01_5000_01 Dose Modifications of Treatment with PEG IFN for Adverse Events or Laboratory Abnormalities by HBeAg Status
Protocol(s): MV22009
Analysis: Safety

	HBeAg positive n = 863 No. (%)	HBeAg negative n = 890 No. (%)	Unknown n = 48 No. (%)	Total n = 1801 No. (%)
Patients with Dose Modification	73 (8)	89 (10)	1 (2)	163 (9)
ALT disorder	3 (<1)	5 (<1)	0 (0)	8 (<1)
Anemia	0 (0)	0 (0)	0 (0)	0 (0)
Asthenia	1 (<1)	3 (<1)	0 (0)	4 (<1)
Depression	1 (<1)	5 (<1)	0 (0)	6 (<1)
Infection	2 (<1)	3 (<1)	0 (0)	5 (<1)
Neutropenia	31 (4)	51 (6)	0 (0)	82 (5)
Thrombocytopenia	14 (2)	15 (2)	0 (0)	29 (2)
Other	24 (3)	11 (1)	1 (2)	36 (2)
Adverse Events	70 (8)	76 (9)	1 (2)	147 (8)
ALT disorder	2 (<1)	4 (<1)	0 (0)	6 (<1)
Anemia	0 (0)	0 (0)	0 (0)	0 (0)
Asthenia	1 (<1)	3 (<1)	0 (0)	4 (<1)
Depression	1 (<1)	5 (<1)	0 (0)	6 (<1)
Infection	2 (<1)	3 (<1)	0 (0)	5 (<1)
Neutropenia	30 (3)	40 (4)	0 (0)	70 (4)
Thrombocytopenia	13 (2)	12 (1)	0 (0)	25 (1)
Other	24 (3)	11 (1)	1 (2)	36 (2)
Laboratory related	4 (<1)	14 (2)	0 (0)	18 (<1)
ALT disorder	1 (<1)	1 (<1)	0 (0)	2 (<1)
Anemia	0 (0)	0 (0)	0 (0)	0 (0)
Neutropenia	2 (<1)	12 (1)	0 (0)	14 (<1)
Thrombocytopenia	1 (<1)	3 (<1)	0 (0)	4 (<1)
Other	0 (0)	0 (0)	0 (0)	0 (0)

Patients may have more than one reason for dose modification.
Dose modifications include dose reductions and withheld doses but not permanent discontinuation of treatment.

Program : $PROD/cdp10586/i22009b/tmedt01.sas / Output : $PROD/cdp10586/mv22009/reports/tmedt01_5000_01.lst
04SEP2015 17:52 Page 1 of 1

Table 15: Highest ALT Level on-Treatment by HBeAg Status

tal0102_5000_01 Highest ALT Level On-Treatment by HBeAg Status
Protocol(s): MV22009
Analysis: Safety

			ALT Levels									
Analysis Group	N	n	Normal <=1 (xULN)		>1 - 2 (xULN)		>2 - 5 (xULN)		>5 - 10 (xULN)		>10 (xULN)		Missing
HBeAg positive	863	826	139	17%	294	36%	294	36%	73	9%	26	3%	37
HBeAg negative	890	873	142	16%	307	35%	326	37%	76	9%	22	3%	17
Unknown	48	39	8	21%	13	33%	12	31%	5	13%	1	3%	9
Total	1801	1738	289	17%	614	35%	632	36%	154	9%	49	3%	63

ULN = upper limit of normal

Program : $PROD/cdp10586/i22009b/tal0102.sas / Output : $PROD/cdp10586/mv22009/reports/tal0102_5000_01.lst
04SEP2015 17:53 Page 1 of 1

Table 16: Highest ALT Level Post- Treatment by HBeAg Status

tal0103_5000_01 Highest ALT Level Post-Treatment by HBeAg Status
Protocol(s): MV22009
Analysis: Safety

			ALT Levels								
Analysis Group	N	n	Normal <=1 (xULN)		>1 - 2 (xULN)		>2 - 5 (xULN)		>5 - 10 (xULN)		>10 (xULN)		Missing
HBeAg positive	863	725	332	46%	202	28%	128	18%	44	6%	19	3%	138
HBeAg negative	890	775	409	53%	210	27%	101	13%	40	5%	15	2%	115
Unknown	48	41	22	54%	10	24%	5	12%	1	2%	3	7%	7
Total	1801	1541	763	50%	422	27%	234	15%	85	6%	37	2%	260

ULN = upper limit of normal

Program : $PROD/cdp10586/i22009b/tal0103.sas / Output : $PROD/cdp10586/mv22009/reports/tal0103_5000_01.lst
04SEP2015 17:54 Page 1 of 1
