# Supplementary material for: Final analysis of the international observational S-Collate study of peginterferon alfa-2a in patients with chronic hepatitis B
Source: PLoS One. 2020 Apr 10;15(4):e0230893. doi: 10.1371/journal.pone.0230893 (PMC7147799; doi:10.1371/journal.pone.0230893)
Supplement: S1 File — (PDF) [file pone.0230893.s009.pdf]

F. HOFFMANN-LA ROCHE
CLINICAL STUDY PROTOCOL

Protocol MV22009

A Multicenter, Prospective, Observational, Non-Interventional Cohort Study Evaluating On-Treatment Predictors of Response in Subjects with HBeAg positive or HBeAg negative Chronic Hepatitis B Receiving Therapy with PEGASYS® (Peginterferon alfa-2a 40KD)

Protocol Approval

Protocol Number/ Version:	Version A	
Date:	18 December 2008	[redacted]
International Medical Leader:	[redacted] MD	
Biostatistician:	[redacted]	[redacted]

Confidentiality Statement

The information contained in this document, especially unpublished data, is the property of F. Hoffmann-La Roche Ltd/Inc/AG/Roche Global Business (or under its control), and therefore provided to you in confidence as an investigator, potential investigator or consultant, for review by you, your staff and an applicable Independent Ethics Committee/Institutional Review Board. It is understood that this information will not be disclosed to others without written authorization from Roche except to the extent necessary to obtain informed consent from those persons to whom the investigational product may be administered.

SYNOPSIS OF PROTOCOL NUMBER MV22009

TITLE	A Multicenter, Prospective, Observational, Non-Interventional Cohort Study Evaluating On-Treatment Predictors of Response in Subjects with HBeAg positive or HBeAg negative Chronic Hepatitis B Receiving Therapy with PEGASYS® (Peginterferon alfa-2a 40KD)
ACRONYM	S-COLLATE
SPONSOR	F. Hoffmann-La Roche
STUDY PHASE	Non–Interventional
INDICATION	Chronic hepatitis B (CHB)
OBJECTIVES	<u>Primary</u><br>• To assess in routine clinical practice on-treatment predictors of HBsAg clearance in subjects with HBeAg positive or negative chronic hepatitis B virus infection (CHB) receiving therapy with PEGASYS® (Peginterferon alfa-2a 40KD) and followed for up to 3 years after treatment cessation<br><u>Secondary</u><br>• Evaluation of the incidence of sustained suppression of HBV DNA<br>• In subjects with HBeAg positive CHB: incidence of HBeAg seroconversion<br>• Evaluation of the incidence of normalization of serum ALT<br>• To assess in routine clinical practice pre-treatment predictors of HBsAg clearance in subjects with HBeAg positive or negative CHB who receive therapy with PEGASYS® and are followed for up to 3 years<br>• To assess predictors of HBsAg seroconversion in subjects with HBeAg positive or negative CHB<br>• Evaluation of the incidence of clinical endpoints, where data available, in responders versus non-responders to treatment: Death, transplantation, HCC, liver decompensation, development of cirrhosis (in patients without cirrhosis at baseline)
TRIAL DESIGN	Prospective, international, multicenter, observational, non-interventional cohort study in CHB subjects receiving therapy with PEGASYS®.<br>Dosing and treatment duration are at the discretion of the investigator in accordance with local labeling.
NUMBER OF SUBJECTS	1,600-1,800 subjects eligible for standard analysis (~2,000 subjects to be enrolled)<br>(40% of subjects with HBeAg negative CHB and 60% with HBeAg positive CHB to reflect population incidence)
TARGET POPULATION	Adult subjects treated for CHB with PEGASYS® according to standard of care and in line with the current summary of product characteristics (SPC) / local labeling who have no contra-indication to PEGASYS® therapy as per the local label.

LENGTH OF STUDY	Subjects will be observed for the duration of their treatment with PEGASYS® and for up to 3 years thereafter.<br>First subject expected to be enrolled in second Quarter 2009 and the last subject expected to be enrolled 2 years later.
END OF STUDY	End is expected in second Quarter 2015 or when last subject completes observation, if earlier.
INVESTIGATIONAL MEDICAL PRODUCT(S)<br>DOSE/ ROUTE/ REGIMEN	Not Applicable
NON-INVESTIGATIONAL MEDICAL PRODUCT(S)	Not applicable
DOSE/ ROUTE/ REGIMEN	Not Applicable
ASSESSMENTS OF:<br>- EFFICACY	Primary Variable:<br>• HBsAg clearance defined as percentage of subjects who become HBsAg negative during the observation period<br><br>Secondary Variables:<br>• In subjects with **HBeAg positive** CHB:<br>➢ Percentage of subjects with suppression of HBV DNA to <2,000 IU/mL during the observation period<br>➢ HBeAg seroconversion defined as percentage of subjects who become HBeAg negative and anti-HBe positive during the observation period<br>➢ Percentage of subjects with a loss of HBeAg during the observation period<br>➢ Percentage of subjects with HBeAg seroconversion and HBV DNA suppression (<2,000 IU/mL) during the observation period<br>➢ Percentage of subjects with suppression of HBV DNA to <80 IU/mL during the observation period<br><br>• In subjects with **HBeAg negative** CHB:<br>➢ Percentage of subjects with suppression of HBV DNA to <2,000 IU/mL during the observation period<br>➢ Percentage of subjects with suppression of HBV DNA to <80 IU/mL during the observation period<br><br>• Serum ALT and ALT ratio<br>• HBsAg seroconversion defined as percentage of subjects who become HBsAg negative and anti-HBs positive during the observation period<br>Other Variables of Interest for Subset of Subjects with Available Data:<br>• Change in quantitative HBsAg from baseline during the observation period<br>• In subjects with **HBeAg positive** CHB:<br>➢ Change in quantitative HBeAg from baseline during the observation period

	• Incidence of clinical endpoints associated with CHB reported in the medical record: Transplantation, HCC, liver decompensation, development of cirrhosis (in patients without cirrhosis at baseline) until 3 years post treatment<br>• All cause mortality and reason for death
- SAFETY	• Adverse event rate and profile
- PHARMACOKINETICS/ PHARMACODYNAMICS	Not applicable
- PHARMACOECONOMICS/ QUALITY OF LIFE (QOL)	Not applicable
CLINICAL AND LABORATORY ASSESSMENTS TO BE DOCUMENTED IN THE eCRF	The following observations and assessments generally form part of routine clinical practice in management of CHB treatment. Where data are available in the medical record, these will be recorded in the eCRF.<br>• Demographics (age, gender, ethnic origin, height and weight)<br>• CHB pre-treatment characteristics: date and mode of infection, HBeAg status positive or negative, alcohol consumption and if available to the investigator: HBV genotype A-H, liver fibrosis assessed by biopsy or liver elastography or indices calculated from blood tests<br>• Quantitative HBV DNA by PCR Test in IU/mL (test name and lower limit of detection to be provided) prior to, during and after treatment (see data collection overview)<br>• Serum ALT including information on the upper limit of normal of the respective test prior to, during and after treatment (see data collection overview)<br>• In **HBeAg positive** subjects:<br>➢ Qualitative HBeAg prior to, during and after treatment (see data collection overview)<br>➢ Quantitative HBeAg, if available, prior to, during and after treatment (see data collection overview)<br>➢ Anti-HBe<br>• HBsAg test prior to, during and after treatment: either a quantitative HBsAg test (e.g. Abbott Architect® Test or other quantitative test) or,<br>if not available, a qualitative test is acceptable (test name and lower limit of detection to be provided; see data collection overview)<br>• Anti-HBs<br>• Actual doses of PEGASYS® received and reasons for dose changes<br>• Intended and actual duration of PEGASYS® therapy<br>• Concomitant medication for CHB<br>➢ Nucleoside/nucleotide analogues<br>➢ Herbal drugs or vaccination for HBV<br>• Adverse events<br>• Dates of the following clinical events: death, transplantation, HCC, liver decompensation, development of cirrhosis (in patients without cirrhosis at baseline) until 3 years post treatment

STATISTICAL ANALYSES	Sample Size Calculation:<br><br>If in total 2,000 subjects will be enrolled, then it is expected that about 800 subjects (40%) will be HBeAg negative and 1,200 subjects will be HBeAg positive (60%). Based on various long-term studies it can be assumed, that the HBsAg clearance rate at 3 years will have a magnitude of about 10%. If the actual clearance rate is between 9% and 12% then in the entire population the width of the exact 95% confidence intervals for HBsAg clearance is expected to be in the range of ±1.25% to ±1.56% (for 1,600 to 2,000 patients), whereas the corresponding widths for HBeAg negative and positive subjects are expected to be in the range of ±2.00% to ±2.67% (for 600 to 800 patients) and ±1.58% to ±2.00% (for 1,000 to 1,200 patients), respectively.<br><br>Logistic regression analyses are planned to investigate the predictive value of various baseline and early on-treatment factors for HBsAg clearance, both for HBeAg negative and HBeAg positive subjects. If at least 500 HBeAg negative subjects can be included in a logistic regression analysis with a binary response variable Y (e.g. HBsAg clearance 3 years post-treatment) and a binary independent variable X (e.g. HBsAg below/above a certain level at week 12), of which 50% are in group X=0 and 50% are in group X=1 then at least 80% power is given to detect a difference in the response probability of 6% vs. 14% (equivalent to an odds ratio of 0.392). The assumed R^2 of X with other independent variables in the model is 0.1. The power will be higher, if more subjects (e.g. in HBeAg positive subjects) can be considered in the multiple logistic regression (MLR) or if the odds-ratio is lower than 0.392.<br><br>Analysis Plan<br><br>All statistical analyses will be grouped by HBeAg positive and HBeAg negative subjects.<br><br>Response rate for all binary endpoints will be calculated as the number of subjects with the respective response divided by the number of subjects in the respective population. Exact 95% (2-sided) confidence interval from the binomial distribution will be provided for the various response variables.<br><br>MLR analyses will be performed to examine the predictive value of various baseline factors and early on-treatment factors (e.g. HBsAg level at week 12) on efficacy endpoints. The positive and negative predictive values of virological response at weeks 12 and 24 on the achievement of HBsAg clearance at post-treatment time points will be examined for HBeAg positive and negative subjects separately.<br><br>The primary and secondary efficacy endpoints will be analyzed both for the ITT and the standard population.

Data Collection Overview

A Multicenter, Prospective, Observational, Non-Interventional Cohort Study Evaluating On-Treatment Predictors of Response in Subjects with HBeAg positive or HBeAg negative Chronic Hepatitis B Receiving Therapy with PEGASYS® (Peginterferon alfa-2a 40KD)

**Assessment / Procedure** (optional, available data will be collected)	**Pre-Therapy**	**During PEGASYS® Therapy (weeks)**				**Post PEGASYS® Therapy (up to 36 months)**			
Weeks from start of therapy	**-24 to 0**	Any time points, e.g. weeks:							
		**12**	**24**	**36**	**48**				
**Months from end of therapy**						**Any time points, e.g. months:**			
						**6**	**12**	**24**	**36**
Informed consent *	×								
Demographics	×								
Medical history	×								
Selection criteria according to SPC/local labeling	×								
Exclusion of co-infection with HAV, HCV, HIV	×								
Confirmation that female subject is not pregnant	×								
Genotyping of HBV	×								
Quantitative HBV DNA in IU/mL	×**	×	×	×	×	×	×	×	×
Serum ALT	×**	×	×	×	×	×	×	×	×
In HBeAg positive subjects: qualitative HBeAg, anti-HBe	×	×	×	×	×	×	×	×	×
In HBeAg positive subjects: quantitative HBeAg	×	×	×	×	×	×	×	×	×
HBsAg Test (quantitative test e.g. Abbott Architect® Test or other quantitative test or, if not available, qualitative test), anti-HBs	×	×	×	×	×	×	×	×	×
Liver assessment (e.g. biopsy)	×								
PEGASYS® exposure		×	×	×	×				
Concomitant HBV medication	×	×	×	×	×	×	×	×	×
Occurrence date of death, transplantation, HCC, liver decom-pensation, development of cirrhosis		×	×	×	×	×	×	×	×
Adverse Events		×	×	×	×	×	×	×	×

* where local regulations allow or require it, informed consent may be given up to 4 weeks after treatment initiation

** if available 2 values within the 24 weeks prior to treatment initiation should be documented, one as close as possible to treatment initiation

TABLE OF CONTENTS ... Page

PART I: STUDY DESIGN AND CONDUCT ... 12

1. BACKGROUND AND RATIONALE ... 12
   - 1.1 Background ... 12
   - 1.2 Rationale ... 14
2. OBJECTIVES OF THE STUDY ... 15
   - 2.1 Primary Objective ... 15
   - 2.2 Secondary Objectives ... 15
3. COHORT STUDY DESIGN ... 15
   - 3.1 Overview of Cohort Study Design ... 15
   - 3.2 Number of Subjects and Enrollment into Cohort Study ... 16
   - 3.3 Centers ... 16
4. COHORT STUDY POPULATION ... 17
   - 4.1 Target Population ... 17
   - 4.2 Concomitant Medication and Treatment ... 17
5. ASSESSMENTS FOR DOCUMENTATION ... 18
   - 5.1 Data Collection Overview ... 18
   - 5.2 Pre-Therapy Data for Documentation in this Cohort Study ... 19
   - 5.3 Data Collected During Observation Period ... 20
   - 5.3.1 Ongoing Laboratory Data Collected in Relation to Therapy Response ... 20
   - 5.3.2 Documentation of PEGASYS® and other HBV Therapy ... 20
   - 5.3.3 Safety Assessments ... 20
6. END POINTS OF THE STUDY ... 21
   - 6.1 Primary Variable ... 21
   - 6.2 Secondary Efficacy Variables ... 21
   - 6.3 Other Secondary Variables of Interest for Subset of Subjects with Available Data ... 21
   - 6.4 Other Secondary Variables ... 22
   - 6.5 Secondary Safety Variables ... 22
7. SAFETY ISSUES ... 22
   - 7.1 Adverse Events and Laboratory Abnormalities ... 22
   - 7.1.1 Clinical Adverse Events ... 22
   - 7.1.1.1 Intensity ... 22
   - 7.1.1.2 Relationship ... 22
   - 7.1.2 Laboratory Test Abnormalities ... 24

7.2 Handling of Safety Parameters ... 24
7.2.1 Serious Adverse Events (Immediately Reportable to Roche) ... 24
7.2.2 Treatment and Follow-up of Adverse Events ... 26
7.2.3 Follow-up of Abnormal Laboratory Test Values ... 26
7.2.4 Pregnancy ... 26
7.3 Premature Withdrawal ... 26

8. STATISTICAL CONSIDERATIONS AND ANALYTICAL PLAN ... 26
8.1 General ... 26
8.2 Primary and Secondary Study Variables ... 27
8.2.1 Primary Variable ... 27
8.2.2 Secondary Efficacy Variables ... 28
8.2.3 Other Secondary Variables of Interest for Subset of Subjects with Available Data ... 28
8.2.4 Other Secondary Variables ... 29
8.2.5 Safety Variables ... 29
8.3 Statistical and Analytical Methods ... 29
8.3.1 Analysis Plan ... 29
8.3.2 Types of Analyses ... 31
8.3.2.1 Definition of Analysis Populations ... 31
8.3.2.2 Exclusion of Data from Analysis ... 31
8.3.2.3 Subgroup Analyses ... 32
8.3.2.4 Safety Data Analysis ... 32
8.3.2.5 Interim Analysis ... 33
8.4 Sample Size ... 33

9. DATA COLLECTION, MANAGEMENT AND QUALITY ASSURANCE ... 35

10. REFERENCES ... 36

PART II: ETHICS AND GENERAL STUDY ADMINISTRATION ... 40

11. ETHICAL ASPECTS ... 40
11.1 Guidelines for Epidemiological Studies ... 40
11.2 Informed Consent ... 40
11.3 Independent Ethics Committees/Institutional Review Board ... 40

12. CONDITIONS FOR MODIFYING THE PROTOCOL ... 41

13. CONDITIONS FOR TERMINATING THE STUDY ... 41

14. STUDY DOCUMENTATION, ECRFS AND RECORD KEEPING ... 41
14.1 Investigator's Files / Retention of Documents ... 41

14.2 Source Documents and Background Data 42
14.3 Audits and Inspections 42
14.4 Electronic Case Report Forms 42
14.5 Contract Research Organizations 43

15. MONITORING THE STUDY 43

16. CONFIDENTIALITY OF TRIAL DOCUMENTS AND SUBJECT RECORDS 43

17. PUBLICATION OF DATA AND PROTECTION OF TRADE SECRETS 43

LIST OF TABLES Page

Table 1 Data collected from the pre-therapy period 19
Table 2 Analysis Time Windows 27
Table 3 Cross-tabulation of Early Virological Response and HBsAg clearance 30
Table 4 Precision of Exact 95% Confidence Intervals for HBsAg clearance rate 34

LIST OF APPENDICES Page

Appendix 1 Child-Pugh Classification of Severity of Liver Disease 39

GLOSSARY OF ABBREVIATIONS

Ab	Antibody
AE	Adverse event
Ag	Antigen
ALT (SGPT)	Alanine aminotransferase
ANOVA	Analysis of variance
AP	Alkaline phosphatase
AST (SGOT)	Aspartate aminotransferase
BP	Blood pressure
CHB	Chronic hepatitis B
CHC	Chronic hepatitis C
CI	Confidence interval
CRA	Clinical Research Associate
CXR	Chest x-ray
eCRF	Electronic Case Report Form
EDC	Electronic Data Capture
GPP	Good Pharmacoepidemiology Practices
GCP	Good Clinical Practice
HAV	Hepatitis A virus
HBcAg	Hepatitis B core antigen
HBeAg	Hepatitis B envelope antigen
HBsAg	Hepatitis B surface antigen
HBV	Hepatitis B virus
HCC	Hepatocellular carcinoma
HCG	Human chorionic gonadotropin
HCV	Hepatitis C virus
HIV	Human immunodeficiency virus
ICH	International Conference on Harmonization
IDB	Investigational Drug Brochure
IEC	Independent Ethics Committee

GLOSSARY OF ABBREVIATIONS (cont'd)

IFN	Interferon alpha
IRB	Institutional Review Board
IgM	Immunoglobulin M antibody
ITT	Intent-to-treat
iv	Intravenous
LFT	Liver function test
μg	Microgram
MIU	Million International Units
mL	Milliliter
MLR	Multiple logistic regression
mRNA	Messenger ribonucleic acid
ng	Nanogram
PCR	Polymerase chain reaction
PD	Pharmacodynamic
PE	Pharmacoeconomic
PEGASYS®	Peginterferon alfa-2a 40 KD
PK	Pharmacokinetic
po	per os
PR	Pulse rate
QoL	Quality of life
RNA	Ribonucleic acid
sAE	Serious adverse event
sc	Subcutaneous
SI	Système International
SRB	Safety Review Board
t.b.d.	To be determined
tiw	Three times per week
ULN	Upper limit of normal
WBC	White blood count

PART I: STUDY DESIGN AND CONDUCT

1. BACKGROUND AND RATIONALE

1.1 Background

Hepatitis B virus (HBV) constitutes a global health threat. Up to 2 billion people worldwide have been infected, of these 400 million suffer from chronic HBV infection resulting in 520,000 deaths each year [1, 2]. Since the early 1980's the infection can be effectively prevented through vaccination [3]. It is now one of the most widely used vaccines in the world and the prevalence of HBV carriers has dropped dramatically in the impacted populations. However, the burden on healthcare systems from the existing pool of chronically infected people is still increasing, since the long-term complications of chronic hepatitis B only become apparent after about 20–30 years.

Carriers of HBV are at increased risk of developing serious complications, including cirrhosis, hepatic decompensation and hepatocellular carcinoma (HCC). Fifty percent of HCC are caused by chronic HBV infection. [1, 4, 5]

The prevalence of HBV infection and patterns of transmission vary greatly throughout the world [1]. In regions with high HBV endemicity (prevalence above 8%), the source of infection is mainly vertically, i.e. perinatal transmission from mother to newborn or infection occurs during early childhood. Immunization programs in neonates have resulted in a dramatic reduction in the prevalence of HBV in children. Areas with low epidemicity (<1%) include Europe and North America. There the source of infection is mainly through sexual contacts and needle sharing among injection drug users [1].

The risk of developing chronic HBV infection after acute exposure ranges from 90% in newborns from infected mothers to 25-30% in children under 5 and declining rapidly with age to only about 5 % in adults [4]. Acute infection develops into chronic if immunological responses to HBV are not induced or are not capable of controlling the virus [6]. Transition from acute to chronic infection appears to represent a failure of immune clearance in virus-infected cells and is marked by persistence of high levels of HBV DNA and HBeAg in serum [7].

The definition of chronic HBV infection is the persistence of hepatitis B surface antigen (HBsAg) for more than 6 months, which was the first hepatitis B-related antigen to be discovered [8]. The natural history of chronic hepatitis B is complex and not completely defined; reviewed in [9]. Initially, chronic disease is characterized (particularly when infected vertically) for one or two decades by an immunotolerant phase where viral loads are high and immune response and active liver inflammation are low. It is thought that very little liver injury occurs during this phase.

During this period the virus produces hepatitis B 'e' antigen (HBeAg), which is thought to aid immune tolerance. This phase of the disease can be terminated by the immune system by achieving a measure of control over the viral infection characterized by a loss of the viral HBe antigen from the circulation and replacement by host derived antibodies, anti-HBe. This transition can be preceded by marked elevations in serum ALT levels, decreasing concentration of HBV

DNA in serum and appearance of HBcAg-specific T cells in the circulation. Following this HBeAg seroconversion, the disease can enter a third phase of relative inactivity and the HBeAg seroconverted status is associated with a considerable improvement in long term morbidity and mortality.

Such HBeAg seroconversion can occur spontaneously at rates of 5-10% per annum [1]. This phase is also termed the inactive carrier state. It may lead to resolution of HBV infection where serum HBsAg becomes undetectable and anti-HBs is detected. Clearance of HBsAg is the hallmark of successful immunological control of HBV infection and is associated with favorable clinical outcomes including lower incidence of cirrhosis, HCC and improved survival [10, 1, 4, 5, 29].

Unfortunately, in some patients HBeAg seroconversion is accompanied by the selection of HBV variants with mutations in the pre-core/core promoter region which reduce or abolish expression of HBeAg [11] thereby evading the immune control. A proportion of these HBeAg negative patients may develop higher levels of HBV replication and progress to a further late phase of reactivated, so called HBeAg-negative chronic hepatitis [1]. HBeAg-negative chronic hepatitis is thus characterized by detection of HBsAg and anti-HBe without HBeAg in serum, detectable HBV DNA and elevated aminotransferase levels. The average age of these patients at diagnosis is 36-45 years, i.e. they are older than patients with HBeAg positive chronic hepatitis, representing a later and more severe phase in the course of HBV infection, characterized by progressive liver damage [12]. The disease has a poor prognosis, with only rare incidences of spontaneous remission; its prevalence is increasing throughout the world.

Interferon (IFN) was the first therapy found to be effective in treating chronic hepatitis B in the early 1990's.

Hoffmann-La Roche has developed a chemically modified IFN by the covalent attachment of a branched methoxy 40KD polyethylene glycol moiety to IFN [13]. Peginterferon alfa-2a 40KD (PEGASYS®) has a decreased systemic clearance rate and a rapid but sustained absorption so that PEGASYS® circulates in the blood much longer than does the parent compound [13] allowing for a more convenient once-weekly dosing as compared to 3 weekly injections with standard IFN.

In February 2005 the EMEA (European Agency for the Evaluation of Medicinal Products) and in May 2005 the FDA (Food and Drug Administration) have granted final marketing approval for PEGASYS® (peginterferon alfa-2a 40KD) in the indication hepatitis B. The approval for hepatitis C had been granted already in 2001.

The efficacy of PEGASYS® in chronic hepatitis B was first shown in a phase II study where 194 subjects with HBeAg positive CHB were randomly assigned to PEGASYS® doses of 90, 180 and 270 μg weekly or the licensed dose of the parent conventional IFN for 24 weeks. The primary endpoint of this study was the combined response of HBeAg loss, HBV DNA suppression and normalization of ALT 24 weeks after treatment cessation. The analysis of this pilot trial revealed a two fold improvement in efficacy of PEGASYS® over unpegylated IFN (24 vs.12 %) and the 270 μg dose did not show any indication of additional benefit [14].

Following completion of the phase II study, two large-scale pivotal studies of PEGASYS® in patients with HBeAg positive and patients with HBeAg negative CHB were conducted.

In the first phase III study 814 HBeAg positive CHB patients were randomized to receive PEGASYS® monotherapy or PEGASYS® plus lamivudine or lamivudine alone for 48 weeks. Response assessed 24 weeks after treatment cessation showed the highest rate of HBeAg seroconversion of 32% in the PEGASYS® monotherapy group as compared to 27% and 19%, respectively. At the end of treatment viral suppression was strongest in the combination therapy arm, however, this did not translate into long-term efficacy [15].

The second phase III study was investigating 552 HBeAg negative CHB patients who were randomized to the same treatment regimens as above (i.e. PEGASYS® monotherapy or PEGASYS® plus lamivudine or lamivudine alone for 48 weeks). Virologic response (defined as HBV DNA <20,000 copies/mL) assessed 24 weeks after treatment cessation was comparable in the groups that received PEGASYS® (43 and 44%) and superior to the lamivudine group (29%) [16]. To determine the long term benefit from treatment, patients were offered participation in an observational follow-up protocol. Data are published for up to 4 years after treatment cessation. The ultimate goal of antiviral therapy is HBsAg clearance which was achieved by 11% of patients in the PEGASYS® groups and 2% in the lamivudine group 4 years after treatment cessation [17]. This rate was slowly increasing from 3% 6 months after treatment cessation to 9% 3 years after treatment cessation and finally 11% 4 years after treatment cessation in the PEGASYS® groups. No patient in the lamivudine group had achieved HBsAg clearance up to 3 years post treatment and only 2% cleared HBsAg 4 years after treatment cessation. [17, 18].

Interestingly, the safety and tolerability of 180 μg PEGASYS® in patients with chronic hepatitis B compares favorably with that observed in hepatitis C, with a lower incidence of common interferon-related adverse events, particularly a lower incidence of depression, and a reduced impact on health related quality of life [19].

1.2 Rationale

Available data from therapy with pegylated interferon reveal that up to 9-15% of HBeAg negative and positive CHB patients have the chance of achieving HBsAg seroconversion 3-5 years after treatment cessation [17, 18, 20, 21]. HBsAg seroconversion represents the control of the virus by the host immune system. It is desirable to identify this group of patients with predictive factors early during the treatment phase.

Data from pivotal studies show that pre-treatment/baseline factors predictive of response in patients with chronic hepatitis B include a high ALT level and a low HBV DNA level [4, 1] as well as high activity scores on liver biopsy (at least A2) [29]. In both HBeAg-negative and HBeAg-positive patients, treatment response to IFN based therapy appeared to be genotype dependent [1, 4]. During treatment a HBV DNA decrease to less than 20,000 IU/mL at week 12 is associated with a 50% chance of HBeAg seroconversion in HBeAg positive patients and with a 50% chance of sustained response in HBeAg negative patients [29, 30, 31]. Another on-treatment predictor for HBeAg seroconversion in HBeAg positive patients seems to be the decrease of quantitative HBeAg at week 24 of treatment [29]. An early decrease in quantitative HBsAg from

baseline to weeks 12 or 24 has been identified as further on-treatment predictor for sustained HBsAg clearance and virological response in HBeAg negative patients [22, 23, 24, 25]. In HBeAg positive patients, on-treatment HBsAg decline during PEGASYS® treatment correlated with HBeAg seroconversion 24 weeks and 1 year post treatment [26].

The purpose of the present observational study is to assess the importance of baseline and on-treatment predictive factors for treatment success following PEGASYS® therapy, in a large population in a real-life setting.

Since the best possible long-term outcome of treatment in any patient with CHB is HBsAg seroconversion [1, 4, 5, 29] it is the major aim of this study to collect data on this parameter in a large population. It can take up to several years for the immune system to achieve HBsAg seroconversion. Therefore the follow-up time after treatment cessation in this study was set to 3 years rather than choosing the option of a roll-over study after the treatment period which proved challenging for data collection in other long-term studies [18, 20].

2. OBJECTIVES OF THE STUDY

2.1 Primary Objective

To assess in routine clinical practice on-treatment predictors of HBsAg clearance in subjects with HBeAg positive or negative chronic hepatitis B virus infection (CHB) receiving therapy with PEGASYS® (Peginterferon alfa-2a 40KD) and followed for up to 3 years after treatment cessation

2.2 Secondary Objectives

- Evaluation of the incidence of sustained suppression of HBV DNA
- In subjects with HBeAg positive CHB: incidence of HBeAg seroconversion
- Evaluation of the incidence of normalization of serum ALT
- To assess in routine clinical practice pre-treatment predictors of HBsAg clearance in subjects with HBeAg positive or negative CHB who receive therapy with PEGASYS® and are followed for up to 3 years
- To assess predictors of HBsAg seroconversion in subjects with HBeAg positive or negative CHB
- Evaluation of the incidence of clinical endpoints, where data available, in responders versus non-responders to treatment: Death, transplantation, HCC, liver decompensation, development of cirrhosis (in patients without cirrhosis at baseline)

3. COHORT STUDY DESIGN

3.1 Overview of Cohort Study Design

This is a prospective, international, multicenter, observational, non-interventional cohort study in subjects with CHB receiving therapy with PEGASYS®.

A cohort of adult subjects who are receiving PEGASYS® treatment for chronic hepatitis B (HBeAg positive or negative) according to standard of care and in line with local labeling will be followed for the duration of their treatment with PEGASYS® and for up to 3 years after PEGASYS® therapy was terminated.

Subjects should not be co-infected with HIV, HAV or HCV.

PEGASYS® dosing and treatment duration are at the discretion of the investigator in accordance with local clinical practice and local labeling.

During PEGASYS® treatment, laboratory assessments are routinely performed in accordance with current guidelines and local standard of care, and it is routine clinical practice to closely monitor subjects with HBV over long periods of time, particularly after a treatment course with Interferon. During this cohort study, available data from the medical record relevant for treatment outcome will be documented in the electronic Case Report Form (eCRF) during treatment with PEGASYS® and for up to 3 years after PEGASYS® therapy was terminated (please refer to Data Collection Overview, section 5.1).

Subjects withdrawn from treatment should remain in this observational cohort and data documentation should continue if any possible even if the subject will receive other therapy for CHB (e.g. nucleoside/nucleotide analogues or IFN). Such medication should be recorded in the eCRF.

The end of study will be the date of the last visit of the last subject undergoing the study. Last subject, last visit is **either** the date of the last subject visit to complete the study, **or** the date at which the last data point from the last subject, which is required for statistical analysis (i.e. key safety and efficacy results for decision making) was received, whichever is the later date.

3.2 Number of Subjects and Enrollment into Cohort Study

Subjects qualifying for the cohort will be assigned to individual study subject numbers in the order of enrollment. Each participating center will be identified by a unique center number assigned by Roche or its designee. In order to obtain data from 1,800 subjects eligible for standard analysis (8.3.2.2), ~2,000 subjects will be observed. Should less subjects than expected with sufficient data for standard analysis be available from routine clinical practice, up to 3,000 subjects might be observed.

For each new subject, the investigator or designee will create a new subject file on the web-based eCRF and a subject number will automatically be allocated by the system.

3.3 Centers

This study will be conducted at ~ 250 centers in Austria, Bosnia, Bulgaria, Brazil, Canada, China, France, Germany, Hong Kong, India, Indonesia, Ireland, Italy, Jordan, Korea, Lebanon, Macedonia, Mexico, Slovakia, Slovenia, Switzerland and Thailand.
Further countries and centers may be added or substituted if underperforming.

4. COHORT STUDY POPULATION

4.1 Target Population

Adult subjects receiving treatment for CHB with PEGASYS® according to standard of care and in line with the current summary of product characteristics (SPC) [27] / local labeling who have no contra-indication to PEGASYS® therapy as per the local label are eligible for this cohort:

- Male and female subjects ≥18 years of age
- HBeAg positive or HBeAg negative serologically proven chronic hepatitis B (CHB) with or without cirrhosis
- Elevated serum ALT >ULN (upper limit of normal) but ≤10 × ULN according to local label
- Subjects with no contra-indications to PEGASYS® therapy as detailed in the label (Hypersensitivity to the active substance, to alpha interferons, or to any of the excipients; Autoimmune hepatitis; Severe hepatic dysfunction or decompensated cirrhosis of the liver; A history of severe pre-existing cardiac disease, including unstable or uncontrolled cardiac disease in the previous six months)
- Subjects who are not co-infected with HAV, HCV or HIV
- Subjects should not receive concomitant therapy with telbivudine (because concomitant peginterferon therapy is contraindicated according to the telbivudine label)
- Female subjects not pregnant or breast feeding when PEGASYS® treatment commenced, and aware of the requirement to use an effective method of contraception during therapy
- Written informed consent where local regulations allow or require it

4.2 Concomitant Medication and Treatment

Concomitant medication for HBV (e.g. nucleoside/nucleotide analogues) will be documented in the eCRF with start and end dose and treatment duration (start and stop dates) as well as reasons for early withdrawal of therapy. Should a subject receive herbal drugs or HBV vaccination during the observation period this should be documented adequately.

5. ASSESSMENTS FOR DOCUMENTATION

5.1 Data Collection Overview

A Multicenter, Prospective, Observational, Non-Interventional Cohort Study Evaluating On-Treatment Predictors of Response in Subjects with HBeAg positive or HBeAg negative Chronic Hepatitis B Receiving Therapy with PEGASYS® (Peginterferon alfa-2a 40KD)

Assessment / Procedure (optional, available data will be collected)	Pre-Therapy	During PEGASYS® Therapy (weeks)				Post PEGASYS® Therapy (up to 36 months)			
Weeks from start of therapy	-24 to 0	Any time points, e.g. weeks:							
		12	24	36	48				
Months from end of therapy						Any time points, e.g. months:			
						6	12	24	36
Informed consent *	×								
Demographics	×								
Medical history	×								
Selection criteria according to SPC/local labeling	×								
Exclusion of co-infection with HAV, HCV, HIV	×								
Confirmation that female subject is not pregnant	×								
Genotyping of HBV	×								
Quantitative HBV DNA in IU/mL	×**	×	×	×	×	×	×	×	×
Serum ALT	×**	×	×	×	×	×	×	×	×
In HBeAg positive subjects: qualitative HBeAg, anti-HBe	×	×	×	×	×	×	×	×	×
In HBeAg positive subjects: quantitative HBeAg	×	×	×	×	×	×	×	×	×
HBsAg Test, (quantitative test e.g. Abbott Architect® Test or other quantitative test or, if not available, qualitative test), anti-HBs	×	×	×	×	×	×	×	×	×
Liver assessment (e.g. biopsy)	×								
PEGASYS® exposure		×	×	×	×				
Concomitant HBV medication	×	×	×	×	×	×	×	×	×
Occurrence date of death, trans-plantation, HCC, liver decom-pensation, development of cirrhosis		×	×	×	×	×	×	×	×
Adverse Events		×	×	×	×	×	×	×	×

* where local regulations allow or require it, informed consent may be given up to 4 weeks after treatment initiation

** if available 2 values within the 24 weeks prior to treatment initiation should be documented, one as close as possible to treatment initiation

5.2 Pre-Therapy Data for Documentation in this Cohort Study

The following data collection overview (Table 1) represents assessments that are routinely performed as part of current standard of care in the majority of centers prior to the initiation of therapy with PEGASYS®. Data available from information documented in the subject's medical records will be entered into the eCRF. Pre-treatment data may be documented retrospectively up to 4 weeks after treatment initiation.

Table 1 Data collected from the pre-therapy period

Demographics	Age (date of birth), gender, ethnic origin, height and body weight
Medical History	Possible mode and assumed year of infection (perinatal, injection drug use, sexual, occupational, transfusion, unknown, other)<br><br>Alcohol consumption (regular alcohol consumption, average number of units/drinks per week)
Prior and concomitant medication for HBV	Standard interferon, pegylated interferon, nucleoside/nucleotide analogues: start of therapy, dosage regimen (start and end dose), cessation of therapy.<br>In addition herbal drugs and vaccination for HBV
HBV disease characteristics	HBV genotype (A-H) (if available)
Virology /Immunology (Test done within 24 weeks prior to treatment initiation)	Quantitative HBV DNA* in IU/mL (Test name and lower limit of detection to be provided)<br><br>In HBeAg positive subjects: Qualitative HBeAg and anti-HBe, quantitative HBeAg if available (Test name and lower limit of detection to be provided)<br><br>HBsAg Test, either a quantitative HBsAg Test (e.g. Abbott Architect® Test or other quantitative test) or, if not available, a qualitative test is acceptable (test name and lower limit of detection to be provided) and anti-HBs<br><br>HDV-RNA in patients with hepatitis delta co-infection
Clinical Chemistry (Test done within 24 weeks prior to treatment initiation)	ALT * including information on the upper limit of normal of the respective test
Liver assessment (if available):	Results from liver biopsy and documentation of the histopathological scoring system used (e.g. METAVIR, Ishak, Knodell, Scheuer)<br><br>From non-invasive methods:<br>- liver elastography (specify kPa assessed by FibroScan)<br>- indices calculated from blood tests

* if available 2 values within the 24 weeks prior to treatment initiation should be documented, one as close as possible to treatment initiation

5.3 Data Collected During Observation Period

5.3.1 Ongoing Laboratory Data Collected in Relation to Therapy Response

During PEGASYS® treatment laboratory assessments are routinely performed in accordance with current guidelines and local standard of care, and it is routine clinical practice to closely monitor subjects with HBV over long periods of time, particularly after a treatment course with Interferon. When performed during the observational period, available results from the range of assessments described below will be documented in the eCRF. Please refer to Data Collection Overview (section 5.1) for time windows of documentation. Most data will be documented around week 12, 24, 36 and 48 of therapy, twice a year for one year after treatment termination and then once yearly for up to 3 years after treatment discontinuation.

Laboratory assessments:

- Quantitative HBV DNA in IU/mL (along with test name and lower limit of detection)
- Serum ALT including information on the upper limit of normal of the respective test
- In HBeAg positive subjects:
  - Qualitative HBeAg
  - Quantitative HBeAg, if available (along with test name and lower limit of detection)
  - Anti-HBe
- HBsAg test
  - Quantitative HBsAg test (e.g. Abbott Architect® Test or other quantitative test) or, if not available, a qualitative test is acceptable (test name and lower limit of detection to be provided)
- Anti-HBs

5.3.2 Documentation of PEGASYS® and other HBV Therapy

The starting dose, dose adjustments, the intended and actual treatment duration of PEGASYS® therapy will be documented in the eCRF as well as reasons for dose changes and duration of therapy that is shorter or longer than originally planned.

In addition, concomitant medication for HBV (e.g. nucleoside/nucleotide analogues) will be documented with start and end dose and treatment duration (start and stop dates) as well as reasons for early withdrawal of therapy during the total study phase including follow-up. Should a subject receive herbal drugs or HBV vaccination during the observation period this should be documented adequately.

5.3.3 Safety Assessments

Clinical endpoints associated with CHB will be recorded during and up to 3 years after treatment: Death, transplantation, HCC, liver decompensation, development of cirrhosis (in patients without cirrhosis at baseline) (if data available).

Clinical adverse events (AEs) will be recorded in the eCRF during the total observation period of up to 4 years.

6. END POINTS OF THE STUDY

6.1 Primary Variable

HBsAg clearance defined as percentage of subjects who become HBsAg negative during the observation period.

6.2 Secondary Efficacy Variables

- In subjects with **HBeAg positive** CHB:
  - Percentage of subjects with suppression of HBV DNA to <2,000 IU/mL during the observation period
  - HBeAg seroconversion defined as percentage of subjects who become HBeAg negative and anti-HBe positive during the observation period
  - Percentage of subjects with a loss of HBeAg during the observation period
  - Percentage of subjects with HBeAg seroconversion and HBV DNA suppression (<2,000 IU/mL) during the observation period
  - Percentage of subjects with suppression of HBV DNA to <80 IU/mL during the observation period

- In subjects with **HBeAg negative** CHB:
  - Percentage of subjects with suppression of HBV DNA to <2,000 IU/mL during the observation period
  - Percentage of subjects with suppression of HBV DNA to <80 IU/mL during the observation period

- Serum ALT and ALT ratio
- HBsAg seroconversion defined as percentage of subjects who become HBsAg negative and anti-HBs positive during the observation period

6.3 Other Secondary Variables of Interest for Subset of Subjects with Available Data

- Change in quantitative HBsAg from baseline during the observation period
- In subjects with **HBeAg positive** CHB:
  - Change in quantitative HBeAg from baseline during the observation period

- Incidence of clinical endpoints associated with CHB reported in the medical record: Transplantation, HCC, liver decompensation, development of cirrhosis (in patients without cirrhosis at baseline) until 3 years post treatment
- All cause mortality and reason for death

6.4 Other Secondary Variables

The following confounding variables will be recorded to assess their impact on efficacy:

- PEGASYS® exposure as assessed by documentation of starting dose and dose changes including reasons

6.5 Secondary Safety Variables

- Adverse event rate and profile

7. SAFETY ISSUES

7.1 Adverse Events and Laboratory Abnormalities

7.1.1 Clinical Adverse Events

Per the International Conference of Harmonization (ICH), an Adverse Event (AE) is any untoward medical occurrence in a patient or clinical investigation subject administered a pharmaceutical product and which does not necessarily have to have a causal relationship with this treatment. An AE can therefore be any unfavorable and unintended sign (including an abnormal laboratory finding, for example), symptom, or disease temporally associated with the use of a medicinal product, whether or not considered related to the medicinal product. Pre-existing conditions which worsen during a study are to be reported as AE.

All clinical adverse events (AEs) encountered during the clinical study will be reported on the AE page of the eCRF. Intensity of adverse events will be graded on a three-point scale (mild, moderate, severe) and reported in detail as indicated on the eCRF. Relationship of the adverse event to the treatment should also be assessed.

7.1.1.1 Intensity

Mild:	discomfort noticed but no disruption of normal daily activity
Moderate:	discomfort sufficient to reduce or affect normal daily activity
Severe:	incapacitating with inability to work or perform normal daily activity

7.1.1.2 Relationship

Relationship of the adverse event to the treatment should be assessed using the following criteria:

PROBABLE (must have first three)

This category applies to those adverse experiences which are considered, with a high degree of certainty, to be related to the test drug. An adverse experience may be considered probable if:

1. It follows a reasonable temporal sequence from administration of the drug.

2. It could not be reasonably explained by the known characteristics of the patient's clinical state, environmental or toxic factors, or other modes of therapy administered to the patient.

3. It disappears or decreases on cessation or reduction in dose (there are important exceptions when an adverse experience does not disappear upon discontinuation of the drug, yet drug relatedness clearly exists; e.g., 1) bone marrow depression; 2) tardive dyskinesias.)

4. It follows a known pattern of response to the suspected drug.

5. It reappears upon rechallenge.

POSSIBLE (must have first two)

This category applies to those adverse experiences in which the connection with the test drug administration appears unlikely but cannot be ruled out with certainty. An adverse experience may be considered possible if, or when:

1. It follows a reasonable temporal sequence from administration of the drug.

2. It may have been produced by the patient's clinical state, environmental or toxic factors, or their modes of therapy administered to the patient.

3. It follows a known response pattern to the suspected drug.

REMOTE (must have first two)

In general, this category is applicable to an adverse event which meets the following criteria:

1. It does <u>not</u> follow a reasonable temporal sequence from administration of the drug.

2. It could readily have been produced by the patient's clinical state, environmental or toxic factors, or other modes of therapy administered to the patient.

3. It does not follow a known response pattern to the suspected drug.

4. It does not reappear or worsen when the drug is readministered.

UNRELATED

This category is applicable to those adverse experiences which, after careful medical consideration at the time of evaluation, are judged to be clearly and incontrovertibly due to extraneous causes (disease, environment, etc.) and do not meet the criteria for drug relationship listed under REMOTE, POSSIBLE or PROBABLE.

Table for Determining Adverse Events Relationship to Investigational Product				
	Probable	Possible	Remote	Unrelated
Clearly due to extraneous causes	-	-	-	+
Reasonable temporal association with drug administration	+	+	-	-
May be produced by subject's clinical state, etc.	-	+	+	+
Known response pattern to suspected drug	+	+	-	-
Disappears or decreases on cessation or reduction in dose	+	-	-	-
Reappears on rechallenge	+	-	-	-

7.1.2 Laboratory Test Abnormalities

Laboratory test results will be recorded on the laboratory results form of the eCRF.

Study monitors will review eCRF entries on an ongoing basis. Unexplained abnormal laboratory test values should be repeated immediately if medically relevant and followed until either they have returned to the normal range and/or are adequately explained.

Any laboratory result abnormality fulfilling the criteria for a serious adverse event (SAE) should be immediately reported on an SAE reporting form, in addition to being recorded as an AE in the eCRF (see also section 7.2.1 on SAE reporting).

Laboratory test value abnormalities should not be reported as adverse events on the "Adverse Event" electronic form of the eCRF unless the abnormal laboratory assessment meets any one of the criteria listed below:

- is considered to be an SAE
- results in discontinuation from study treatment
- results in a requirement for a change in concomitant therapy (e.g. addition of, interruption of, discontinuation of, or any other change in a concomitant medication, therapy or treatment)

7.2 Handling of Safety Parameters

7.2.1 Serious Adverse Events (Immediately Reportable to Roche)

ANY CLINICAL ADVERSE EVENT OR ABNORMAL LABORATORY TEST VALUE THAT IS **SERIOUS** (INCLUDING DEATH, OVERDOSE OR CONGENITAL ANOMALY) OCCURRING DURING THE COURSE OF THE STUDY, IRRESPECTIVE OF THE TREATMENT RECEIVED BY THE PATIENT, MUST BE REPORTED TO ROCHE (i.e. to designee [redacted], Germany, being the Clinical Research Organization in charge) ON AN SAE REPORTING FORM WITHIN **ONE** WORKING DAY OF OCCURRENCE,

FAX NO: [redacted]

The definition and reporting requirements of the ICH Guideline for Clinical Safety Data Management, Definitions and Standards for Expedited Reporting, Topic E2 will be adhered to.

A serious adverse event is any experience that suggests a significant hazard, contraindication, side effect or precaution. With respect to human clinical experience, this includes any experience which

- is fatal (results in death; NOTE: death is an outcome, not an event);
- is life-threatening (NOTE: the term "life-threatening" refers to an event in which the patient was at immediate risk of death at the time of the event; it does not refer to an event which could hypothetically have caused a death had it been more severe);
- requires inpatient hospitalization or prolongation of an existing hospitalization;
- results in persistent or significant disability/ incapacity;
- is a congenital anomaly/ birth defect;
- is medically significant or requires intervention to prevent one or other of the outcomes listed above

Medical and scientific judgment should be exercised in deciding whether expedited reporting is appropriate in other situations, such as important medical events that may not be immediately life-threatening or result in death or hospitalization but may jeopardize the patient or may require intervention to prevent one of the outcomes listed in the definitions above. These situations should also usually be considered serious.

The term **severe** is a measure of **intensity**, thus a severe adverse event is not necessarily serious. For example, nausea of several hours' duration may be rated as severe, but may not be clinically serious.

A **death** occurring during the study, including the protocol-defined follow-up, which comes to the attention of the investigator, whether considered treatment-related or not, must be reported.

Such preliminary reports will be followed by detailed descriptions later which will include copies of hospital case reports, autopsy reports and other documents when requested and applicable.

Related Serious Adverse Events must be collected and reported regardless of the time elapsed from the last study drug administration, even if the study has been closed.

For serious and all other AEs, the following must be assessed and recorded on the AE page of the eCRF: intensity, relationship to test substance, action taken regarding test substance, and outcome to date.

The investigator must notify the Institutional Review Board (IRB) [Independent Ethics Committee (IEC)] of such an event in writing as soon as it is practical and in accordance with international and local laws and regulations.

Note: The definitions for and procedures for reporting SAEs to Health Authorities will be taken from the ICH guidelines. The definition of "expectedness" will include those events encountered for PEGASYS® in other clinical trials, clinical events described for the first generation

PEGASYS® compound, and AEs listed in the IDB and IDB addenda (provided to all investigators with study documentation).

7.2.2 Treatment and Follow-up of Adverse Events

Patients experiencing AEs should be treated by accepted clinical procedures. All AEs should be followed until resolved or stabilized.

7.2.3 Follow-up of Abnormal Laboratory Test Values

In the event of unexplained abnormal laboratory test values, the tests should be repeated immediately if medically relevant and followed up until they have returned to the normal range and/or an adequate explanation of the abnormality is found. If a clear explanation is established it should be recorded on the eCRF.

7.2.4 Pregnancy

Pregnancy is to be strictly avoided during treatment with PEGASYS®. However, if a female subject becomes pregnant during the study she must be instructed to stop PEGASYS® and immediately inform the investigator. Pregnancies occurring up to 3 months after the completion of PEGASYS® must also be reported to the investigator. The investigator should report all pregnancies within 24 hours to Roche using the Clinical Trial Pregnancy Reporting form.

The investigator should counsel the patient, discuss the risks of continuing with the pregnancy and the possible effects on the fetus. Monitoring of the patient should continue until conclusion of the pregnancy. Information on the outcome of the pregnancy must be provided to the sponsor.

7.3 Premature Withdrawal

Subjects have the right to withdraw from the cohort study at any time for any reason. The investigator also has the right to withdraw subjects from the cohort study if it is in the best interest of the subject. An excessive rate of withdrawals can render the study uninterpretable; therefore, unnecessary withdrawal of subjects should be avoided. Should a subject decide to withdraw, all efforts will be made to complete and report the observations as thoroughly as possible.

Subjects withdrawn from treatment should remain in this observational cohort and data documentation should continue if any possible even if the subject will receive other therapy for CHB (e.g. nucleoside/nucleotide analogues or IFN). Such medication should be recorded in the eCRF.

8. STATISTICAL CONSIDERATIONS AND ANALYTICAL PLAN

8.1 General

Due to the non-interventional design of the study, the time points for laboratory measurements are not mandatory. However all available measurements (for details see Section 5) prior and after

start of PEGASYS® treatment, up to 3 years post-treatment, should be entered into the eCRF. These measurements will be assigned to the following time windows:

Table 2 Analysis Time Windows

Phase	Time point	Time Window
Pre-Therapy	Baseline	Last value prior to start of study treatment (i.e. ≤study day 1)
Treatment	Weeks from start of treatment	Study day during treatment period
	12	56 - 126
	24	127 - 210
	36	211- 294
	48	295 - 378
Post-Treatment	Years after actual end of treatment	Follow-up day after stop of treatment
	0.5	92 - 273
	1	274 - 547
	2	548 - 912
	3	913 – 1278

All statistical analyses will be grouped by HBeAg positive and HBeAg negative subjects.

8.2 Primary and Secondary Study Variables

8.2.1 Primary Variable

The primary variable of the study is the post-treatment HBsAg clearance defined as the percentage of subjects who are HBsAg negative at three years post-treatment. For subjects who have multiple HBsAg measurements in the time interval specified for 3 years post-treatment (see Table 2) the last available measurement will be used for analysis. If a subject has no HBsAg measurements at 3 years post-treatment, but at the two previous consecutive post-treatment measurements (at least 270 days apart) the two HBsAg measurements and the two HBV DNA measurements are negative, then the last HBsAg value will be carried forward to all following time points until 3 years post-treatment. For all other subjects with missing HBsAg measurements at 3 years, non response regarding HBsAg clearance will be assumed.

Response rate will be calculated as the number of subjects with HBsAg clearance divided by the number of subjects of the respective analysis population (see Section 8.3.2).

8.2.2 Secondary Efficacy Variables

- In subjects with **HBeAg positive** CHB:
  - Percentage of subjects with a suppression of HBV DNA to <2,000 IU/mL by a quantitative PCR Test at the post-treatment time points as specified in Table 2.
  - HBeAg seroconversion defined as percentage of subjects who become HBeAg negative and anti-HBe positive at end of treatment and the post-treatment time points specified in Table 2
  - Percentage of subjects with a loss of HBeAg at end of treatment and the post-treatment time points specified in Table 2.
  - Percentage of subjects with HBeAg seroconversion and HBV DNA suppression (<2,000 IU/mL) at end of treatment and the post-treatment time points specified in Table 2
  - Percentage of subjects with suppression of HBV DNA to <80 IU/mL at end of treatment and the post-treatment time points specified in Table 2

- In subjects with **HBeAg negative** CHB:
  - Percentage of subjects with a suppression of HBV DNA to <2,000 IU/mL by a quantitative PCR Test at the post-treatment time points as specified in Table 2
  - Percentage of subjects with suppression of HBV DNA to <80 IU/mL at end of treatment and the post-treatment time points specified in Table 2

- Percentage of subjects with a HBsAg clearance at end of treatment and at the post-treatment time points specified in Table 2. (with the exception of the primary variable)
- Serum ALT and calculated ALT ratio at the time points specified in Table 2
- Normalization of ALT at the time points specified in Table 2
- Change in quantitative HBV DNA from baseline for the time points specified in Table 2
- HBsAg seroconversion defined as percentage of subjects who become HBsAg negative and anti-HBs positive at end of treatment and the post-treatment time points specified in Table 2

8.2.3 Other Secondary Variables of Interest for Subset of Subjects with Available Data

- Change in quantitative HBsAg from baseline for the time points specified in Table 2
- In subjects with **HBeAg positive** CHB:
  - Change in quantitative HBeAg from baseline for the time points specified in Table 2

- Frequency of clinical endpoints associated with CHB reported in the medical record: liver transplantation, HCC, liver decompensation, development of cirrhosis (in patients without cirrhosis at baseline) until 3 years post-treatment.
- All cause mortality and reason of death

8.2.4 Other Secondary Variables

The following confounding variables will be recorded to assess their impact on efficacy:

- PEGASYS® exposure as assessed by documentation of starting dose and dose changes including reasons

8.2.5 Safety Variables

- Adverse event rate and profile

8.3 Statistical and Analytical Methods

8.3.1 Analysis Plan

The demographic data will be summarized as mean, standard deviation, standard error of the mean, median, interquartile range and range for continuous variables, and in tables of frequencies and percentages for categorical variables.

The primary and secondary efficacy endpoints will be analyzed both for the ITT and the standard population. Response rate for all binary endpoints will be calculated as the number of subjects with the respective response divided by the number of subjects in the respective population. Exact 95% (2-sided) confidence interval from the binomial distribution will be provided for the various response variables.

Due to the non-interventional design of the study measurements might be missing at various time points specified in Table 2. Apart from the last value forward approach defined for the primary endpoint (see Section 8.2.1), the following replacement method will be used for all binary response variable in case of missing measurements: If the last available measurement and the next available measurement for the response outcome variable fulfill both the response criteria, then it is assumed that the response criteria is also fulfilled at the interim time points with missing measurements (e.g. HBV DNA <2,000 IU/mL is fulfilled 1 and 3 years post-treatment and the HBV DNA measurement is missing 3 years post-treatment, then HBV DNA <2,000 IU/mL is also assumed for the 2 years post-treatment time point). In all other cases, subjects without measurements at the respective time points will be considered non-responders regarding this response variable.

Multiple logistic regression (MLR) analyses will be performed to examine the predictive value of various baseline factors or early on-treatment factors on efficacy endpoints. In the first step all relevant explanatory factors will be examined in a univariate logistic regression analysis. In the following stepwise selection procedures only those factors will be considered, which were significant at a level of 0.1. This will allow to exclude irrelevant factors with a high number of missing values, because in the stepwise selection process only subjects with a complete set of explanatory variables can be included. In the stepwise model building process, a variable will be added to the model if the adjusted chi-square statistic is significant at the 0.1 level and a variable will be deleted from the model if the Wald chi-square statistic is not significant at the 0.05 level. In a final step the MLR analysis will be repeated adding each factor not considered for the stepwise approach to verify again the relevance of the excluded variable for the selected model.

Demographic and baseline disease characteristics such as age, gender, weight, BMI, region, ALT ratio, genotype, HBsAg level, HBV DNA level, previous interferon treatment for CHB (yes/no) will be examined in the MLR. Early on-treatment factors are for example HBsAg level at weeks 12 and 24 below a certain level (e.g. <1,500 IU/mL in HBeAg positive patients) and/or the change in HBsAg from baseline to week 12 (e.g. at least a 0.5-log drop). For significant predictors odds-ratio and corresponding 95% confidence intervals will be provided.

The positive and negative predictive values of virological response at weeks 12 and 24 on the achievement of HBsAg clearance at post-treatment time points (see Table 1) will be examined for HBeAg positive and HBeAg negative subjects separately.

The following evidence of early virological response (at weeks 12 and 24) will be explored:

- HBsAg <1,500 IU/mL at week 12 (and 24) in HBeAg positive patients
- At least a 0.5-log drop in HBsAg from baseline to week 12 (and 24)
- Other factors or cut-offs identified in the logistic regression analyses described above

The probability that the subject who develops an early virological response will achieve HBsAg clearance post-treatment is called the positive predictive value (PPV) of the early virological response. The probability that the subject who fails to develop an early virological response also will fail to achieve HBsAg clearance post-treatment is called the negative predictive value (NPV) of the early virological response. As an example using data given in Table 3, PPV is calculated as a/(a+c). NPV is calculated as d/(b+d).

Table 3 Cross-tabulation of Early Virological Response and HBsAg clearance

Number of subjects	Clearance	No clearance	Total
Early Virological response			
YES	a	c	a + c
NO	b	d	b + d
TOTAL	a + b	c + d	a + b + c + d

Note: a, b, c, d are number of subjects in each cell

For all PPV and NPV 95% confidence intervals will be calculated.

Sensitivity analyses will be performed to examine the effect and pattern of dropouts and missing values on key efficacy parameters.

One objective of the study is to investigate, whether treatment response has an impact on the following clinical endpoints: time to death of any cause, time to CHB related death, time to liver transplantation, time to HCC and time to liver decompensation. In subjects without liver cirrhosis at baseline also the time to liver cirrhosis will be analyzed. The duration to the occurrence of the events will be counted from start of PEGASYS® treatment. The last assessment the subject is known to be event-free will be used for censoring. Cox-proportional hazard models will be used considering post-baseline response factors (e.g. first time point with HBV DNA <2,000 IU/mL) as explanatory time-dependent covariates. In addition, baseline variables (already listed above for the MLR) will be also considered in the models as possible explanatory covariate.

Change from baseline variables will be summarized using mean, standard deviation, standard error of the mean, median, interquartile range, range and 95% confidence intervals for the mean.

8.3.2 Types of Analyses

8.3.2.1 Definition of Analysis Populations

Intent-to-Treat (ITT) analysis population is defined to include all subjects who received at least one dose of PEGASYS®. The primary and all other secondary efficacy parameters will be analyzed using the ITT population.

Standard analysis population will exclude a treated subject if the subject meets any of the criteria listed in Section 8.3.2.2. The main focus of this population is to perform statistical analyses in subjects with sufficiently complete data (no early dropout, no missing key information) and who will fulfill those inclusion criteria, which can be considered essential for conclusions regarding efficacy. The primary and all other secondary efficacy parameters will be analyzed using the standard population.

Safety analysis population is defined to include only subjects who received at least one dose of PEGASYS® and have at least one post-baseline safety assessment.

It is planned to perform several interim analyses (see Section 8.3.2.5), each considering only those subjects, who have started PEGASYS® treatment prior to an interim specific enrollment cut-off. Furthermore, only those study variables will be analyzed in the interim analysis, which could have been measured prior to the interim specific data cut-off. For example, the first interim analysis focusing on the 6 months post treatment endpoints is planned with the first half of all subjects to be enrolled, as soon as these subjects are treatment free for at least 6 months, so that all their study variables up to 6 months post-treatment should be available for the statistical analysis, if actually measured in the subjects.

In all interim analyses this restriction will be applied to all three analysis populations specified above.

8.3.2.2 Exclusion of Data from Analysis

The following subjects will be excluded from the standard analysis:

1. No positive HBsAg result more than 6 months prior to commencing PEGASYS®

2. No negative anti-HBs results prior to commencing PEGASYS®
3. HBV DNA <2,000 IU/mL (by PCR) at last measurement prior to commencing PEGASYS® therapy
4. Subjects who have previously received pegylated interferon
5. Subjects who are co-infected with HAV, HCV or HIV
6. Subjects with hepatitis delta co-infection
7. Subjects who never took any PEGASYS®
8. Subjects with missing quantitative HBsAg measurement at week 12
9. In HBeAg positive subjects: No positive qualitative HBeAg or no negative anti-HBe within 6 months prior to commencing PEGASYS®
10. In HBeAg negative subjects: No negative qualitative HBeAg or no positive anti-HBe within 6 months prior to commencing PEGASYS®

Additional criteria might be considered when writing the Statistical Analysis Plan.

8.3.2.3 Subgroup Analyses

The following exploratory subgroup analyses will be performed for the primary efficacy endpoint and key secondary efficacy endpoints:

- For subjects with genotypes A, B, C, D
- Subjects with hepatitis delta co-infection (if a sufficient number of patients will be included)
- Subjects with prior standard interferon therapy
- Subjects with prior pegylated interferon therapy

Further subgroups might be specified in the Statistical Analysis Plan.

8.3.2.4 Safety Data Analysis

Adverse events (AEs) will be assigned preferred terms and categorized into body systems according to the Medical Dictionary for Drug Regulatory Affairs (MedDRA) classification of the World Health Organization (WHO) terminology.

The proportion of subjects with AEs will be calculated by dividing the number of subjects who experienced the AE by the number of subjects evaluable for safety analysis. AEs will be summarized by disease groups (HBeAg negative, HBeAg positive and total), by body system and event within each body system.

ALT will be analyzed according to Roche's "International Guideline for the Handling and Reporting of Laboratory Data" [32]. ALT data will be converted from any units not Systeme International (SI) to SI units. Lab data will therefore be processed and reported in terms of SI units. Furthermore ALT will transformed to the Roche standard reference range at the time of conversion to SI units in order to allow comparisons of subjects from different centers.

The following equation will be used for transforming of ALT:

$$R_T = R_U \frac{S_H}{I_H}$$

Where: R_T = transformed result

R_U = untransformed result

S_H = upper limit of Roche standard reference range

I_H = upper limit of investigator's reference range

Changes in ALT and ALT-ratio from baseline will be summarized by the disease group using descriptive statistical methods.

8.3.2.5 Interim Analysis

There will be several interim analyses as soon as a sufficient number of subjects have evaluable data for the various time points. The distribution of the week-12 assessments (i.e. quantitative HBeAg and HBsAg, HBV DNA etc.) will be analyzed after at least 200 subjects have their week 12 data available. A further interim analysis focusing on the 6 months post treatment endpoints is planned with the first half of all subjects to be enrolled, as soon as these subjects are treatment free for at least 6 months and their measured data up to 6 months post-treatment are available in the database. Further interim analyses focusing on later post-treatment outcome variables are scheduled yearly thereafter.

No alpha adjustment is considered necessary, because no confirmatory hypothesis testing will be performed in the study.

8.4 Sample Size

If ~2,000 subjects will be enrolled, then it is expected that about 800 subjects (40%) will be HBeAg negative and 1,200 subjects will be HBeAg positive (60%). If 1,800 subjects are evaluable for the standard population (see 8.3.2.2) the distribution of HBeAg negative and positive subjects is expected to be 720 and 1080 subjects, respectively. Based on various long-term studies it can be assumed, that the HBsAg clearance rate at 3 years will have a magnitude of about 9-12% in HBeAg positive subjects and in HBeAg negative subjects. The precision of the exact 95% confidence intervals for the HBsAg clearance rate is displayed in Table 4 as a function of the number of evaluable subjects in both disease groups (600 to 800 HBsAg negative subjects and 1,000 to 1,200 HBeAg positive subjects, thus 1,600 to 2,000 subjects in total) and the actual clearance rate (range from 9% to 12% [18, 20]). The higher number of subjects refers to the ITT population, whereas the lower number of subjects could be appropriate for the standard population.

Table 4 Precision of Exact 95% Confidence Intervals for HBsAg clearance rate

Clearance rate	Number of subjects	Precision*
9%	600	2.333
	720	2.111
	800	2.000
	1000	1.800
	1080	1.685
	1200	1.584
	1600	1.375
1.	1800	1.334
	2000	1.250
10%	600	2.333
	720	2.223
	800	2.125
	1000	1.900
	1080	1.759
	1200	1.667
	1600	1.500
2.	1800	1.389
	2000	1.300
11%	600	2.500
	720	2.250
	800	2.125
	1000	1.900
	1080	1.871
	1200	1.750
	1600	1.563
3.	1800	1.445
	2000	1.350
12%	600	2.667
	720	2.417
	800	2.250
	1000	2.000
	1080	1.908
	1200	1.834
	1600	1.563
4.	1800	1.500
	2000	1.400

*distance of the lower and upper boundary of the 95% confidence interval from the estimated clearance rate.

Thus, in the entire population with 1,600 to 2,000 patients the width of the exact 95% CI for HBsAg clearance is expected to be in the range of ±1.25% to ±1.56%, whereas the corresponding

widths for HBeAg negative and positive subjects are expected to be in the range of ±2.00% to ±2.67% (for 600 to 800 subjects) and ±1.58% to ±2.00% (for 1,000 to 1,200 subjects), respectively.

Logistic regression analyses are planned to investigate the predictive value of various baseline and early on-treatment factors for HBsAg clearance. A logistic regression of a binary response variable Y (e.g. HBsAg clearance 3 years post-treatment) on a binary independent variable X (e.g. HBsAg below/above a cut-off at week 12) with a sample size of 500 evaluable HBeAg negative subjects (of which 50% are in group X=0 and 50% are in group X=1) achieves at least 80% power to detect a difference in the probability P (Y=1) of 6% vs. 14%. This change corresponds to an odds ratio of 0.392. This calculation assumes that other independent variables will remain in the final model and that R^2 of X with other independent variables is 0.1. R is the multiple correlation coefficient between X and the other remaining covariates. The power will be higher, if more subjects (e.g. in HBeAg positive subjects) can be considered in the MLR or if the odds-ratio is lower than 0.392.

9. DATA COLLECTION, MANAGEMENT AND QUALITY ASSURANCE

Data for this study will be recorded via an Electronic Data Capture (EDC) system using web-based electronic Case Report Forms (eCRFs). It will be transcribed by the site from the paper source documents onto the eCRF.

Accurate and reliable data collection will be assured by verification and cross–check of the eCRFs against the investigator's records by the study monitor (source document verification) following Good Pharmacoepidemiology Practices (GPP) Guidelines [28]. Source document verification will be conducted in 10 – 15% of observed subjects in each participating center.

A comprehensive validation check program utilizing front-end checks in the eCRF and back-end checks in the database will verify the data. Discrepancies and queries will be generated accordingly in the eCRF for online resolution by the investigator at the site.

In addition the eCRF data will be reviewed on an ongoing basis for medical and scientific plausibility.

10. REFERENCES

1. de Franchis R, Hadengue A, Lau G, Lavanchy D, Lok A, McIntyre N et al. EASL International Consensus Conference on Hepatitis B, 13-14 September, 2002, Geneva, Switzerland: Consensus statement (long version). J Hepatol 2003;39 Suppl 1:S3-S25.

2. Lai CL, Ratziu V, Yuen MF, Poynard T.Viral hepatitis B. Lancet 2003;362:2089-94.

3. Blumberg, Baruch. Autobiography. Available from: URL: http//www. nobelprize.org/

4. Lok AS, McMahon BJ. AASLD practice guidelines 2007: chronic hepatitis B. Hepatology 2007;45:507-39.

5. Liaw YF, Leung N, Guan R, Lau GKK, Merican I, McCaughan G et al. Asian-pacific consensus statement on the management of chronic hepatitis B: a 2005 update. Liver International 2005; 25:472-89.

6. Maini MK, Boni C, Lee CK, Larrubia JR, Reignat S, Ogg GS et al. The role of virus-specific CD8(+) cless in liver damage and viral control during persistent hepatitis B virus infection. J Exp Med 2000;191:1269-80.

7. Chisari FV, Ferrari C. Hepatitis B virus immunopathogenesis. Ann Rev Immunol 1995;13:29-60.

8. Blumberg B, Alter HJ, Visnich S. A new antigen in leukemia sera. JAMA 1965; 191:541-46.

9. Hoofnagle JH, Doo E, Liang TJ, Fleischer R, Lok ASF. Management of hepatitis B: summary of a clinical research workshop. Hepatology 2007;45:1056-75.

10. Manesis EK, Hadziyannis ES, Angelopoulou OP, Hadziyannis SJ. Prediction of treatment-related HBsAg loss in HBeAg-negative chronic hepatitis B: a clue from serum HBsAg levels. Antiviral Therapy 2007;12: 73-82.

11. Funk ML, Rosenberg DM, Lok ASF. World-wide epidemiology of HBeAg-negative chronic hepatitis B and associated precore and core promoter variants. J Viral Hepat 2002;9:52-61.

12. Hadziyannis SJ, Vassilopoulos D. Hepatitis B e antigen-negative chronic hepatitis B. Hepatology 2001;34:617-24.

13. Investigator´s Brochure Ro 25-8310 PEGASYS® (Peginterferon alfa-2a) and Ro 20-9963 COPEGUS® (Ribavirin), 10th Version, July 2007.

14. Cooksley WG, Piravisuth T, Lee SD, Mahachai V, Chao YC, Tanwandee T et al. Peginterferon alfa-2a (40kD): an advance in the treatment of hepatitis B e antigen-positive chronic hepatitis B. J Viral Hepatitis 2003; 10:298-305.

15. Lau GKK, Piratvisuth T, Luo KX, Marcellin P, Thongsawat S, Cooksley G et al. Peginterferon Alfa-2a, Lamivudine, and the Combination for HBeAg-Positive Chronic Hepatitis B. N Engl J Med 2005; 352:2682-95.

16. Marcellin P, Lau GKK, Bonino F et al. Peginterferon alfa-2a alone, lamivudine alone, and the two in combination in patients with HBeAg-negative chronic hepatitis B. N Engl J Med 2004;351:1206-17.

17. Marcellin P, Piratvisuth T, Brunetto M, Bonino F, Lau GKK, Farci P, et al. Virological and biochemical response in patients with HBeAg-negative chronic hepatitis B treated with peginterferon alfa-2a (40KD) with or without lamivudine: results of 4-year follow-up. J Hepatol 2008;48 Suppl 2: S46.

18. Marcellin P, Bonino F, Lau GKK, Farci P, Yurdaydin C, Piratvisuth T et al. 3 years post-treatment response after peginterferon alfa-2a for HBeAg-negative chronic hepatitis B. Submitted to Gastroenterology.

19. Marcellin P, Lau GKK, Zeuzem S, Heathcote EJ, Pockros PJ, Reddy KR et al. Comparing the safety, tolerability and quality of life in patients with chronic hepatitis B vs chronic hepatitis C treated with peginterferon alpha-2a. Liver Int. 2008;28(4):477-85.

20. Buster EH, Flink HJ, Cakaloglu Y, Simon K, Trojan J, Tabak F et al. Sustained HBeAg and HBsAg loss after long-term follow-up of HBeAg-positive patients treated with peginterferon alfa-2b. Gastroenterology 2008, 135:459-67

21. Brunetto M, Cavallone D, Moriconi F, Colombatto PU, Moscato G, Maina AM et al. Kinetics of HBsAg decline during and following treatment of CHB: Early and rapid HBsAg decline during peginterferon alfa-2a is predictive of HBsAg clearance. Hepatology 2008; 48 Suppl 4:740A (AASLD 2008).

22. Brunetto M, Bonino F, Lau GKK, Farci P, Yurdaydin C, Piratvisuth T et al. On-treatment HBsAg decline in HBeAg-negative patients as a predictor of response to peginterferon alfa-2a (40KD) therapy 3 years post-treatment: potential for response-guided therapy. J Hepatol 2008;48 Suppl 2:S254.

23. Marcellin P, Brunetto M, Bonino F, Hadziyannis SJ, Kapprell H-P, McCloud PI. In patients with HBeAg-negative chronic hepatitis B HBsAg serum levels early during treatment with peginterferon alfa-2a predict HBsAg clearance 4 years post-treatment. Hepatology 2008; 48 Suppl 4:718A (AASLD 2008).

24. Brunetto M, Bonino F, Marcellin P, Lau GKK, Farci P, Yurdaydin C et al. HBV DNA suppression induced by peginterferon alfa-2a (40KD) but not by lamivudine results in HBsAg loss and seroconversion at 3 years post-treatment. J Hepatol 2008;48 Suppl 2:S254.

25. Rijckborst V, ter Borg MJ, Akarca US, Grima P, Flisiak R, Vafiadis-Zouboulis I et al. Early reduction of serum HBsAg levels in HBeAg-negative chronic hepatitis B patients achieving sustained virological response after peginterferon alfa-2a ± ribavirin treatment. Hepatology 2008; 48 Suppl 4:749A (AASLD 2008).

26. Lau GKK, Marcellin P, Brunetto M, Piratvisuth T, Kapprell H-P, Button P et al. On-treatment HBsAg decline during peginterferon alfa-2a (40KD) ± lamivudine in patients with HBeAg-positive CHB as a potential predictor of durable off-treatment response. Hepatology 2008; 48 Suppl 4:714A (AASLD 2008).

27. PEGASYS® Summary of Product Characteristics, June 2007.

28. Epstein M. International Society of Pharmacoepidemiology. Guidelines for good pharmacoepidemiology practices (GPP). Pharmacoepidemiol Drug Saf 2005 Aug; 14(8):589-95.

29. EASL Clinical Practice Guidelines: Management of Chronic Hepatitis B. J Hepatol 2009;50 in press.

30. Bonino F, Marcellin P, Lau GK, Hadziyannis S, Jin R, Piratvisuth T et al. Predicting response to peginterferon alfa-2a, lamivudine and the two combined for HBeAg-negative chronic hepatitis B. Gut 2007;56:699-705.

31. Fried MW, Piratvisuth T, Lau GK, Marcellin P,Chow WC, Cooksley G et al. HBeAg and hepatitis B virus DNA as outcome predictors during therapy with peginterferon alfa-2a for HBeAg-positive chronic hepatitis B. Hepatology 2008;47:428-34.

32. Roche Pharma Global Development PD GCP supporting document gcp_spt000144, International Standard for the Handling and Reporting of Laboratory Data - Clinical Operating Guideline No. 3007. December 13, 2005.

Appendix 1 Child-Pugh Classification of Severity of Liver Disease

Modified Assessment

Assessment	*Degree of abnormality*	*Score*
Encephalopathy	None<br>Grade 1-2<br>Grade 3-4*	1<br>2<br>3
Ascites	Absent<br>Slight<br>Moderate	1<br>2<br>3
S-Bilirubin (mg/dl)<br><br><br>SI unit = µmol/l)	<2<br>2.0-3<br>>3<br><br><34<br>34-51<br>>51	1<br>2<br>3<br><br>1<br>2<br>3
S-Albumin (g/dl)	>3.5<br>3.5-2.8<br><2.8	1<br>2<br>3
INR	<1.7<br>1.7-2.3<br>>2.3	1<br>2<br>3

*grading according to Trey, Burns and Saunders (1996)

Grade **A (mild)**: Total score of 5 or 6

Grade **B (moderate)**: Total score of 7 to 9

Grade **C (severe)**: Total score of 10 to 15

PART II: ETHICS AND GENERAL STUDY ADMINISTRATION

11. ETHICAL ASPECTS

11.1 Guidelines for Epidemiological Studies

The guidelines for GPP in non-interventional studies will be respected as well as recommendations for non-interventional trials and principles of epidemiology studies. This trial is not in the scope of Good Clinical Practice (GCP) studies.

11.2 Informed Consent

It is the responsibility of the investigator, or a person designated by the investigator (if acceptable by local regulations), to obtain written informed consent from each subject participating in this cohort study, after adequate explanation of the aims, methods, anticipated benefits, and potential hazards of the observation. The investigator or designee must also explain that the subjects are completely free to refuse to enter the cohort study or to withdraw from it at any time, for any reason. The eCRF for this study contains a section for documenting informed subject consent, and this must be completed appropriately where local regulations allow or require it. Informed consent may be given up to 4 weeks after treatment initiation.

Subjects must agree to the anonymous data collection, the pooling of data with similar scientific data, and the possibility of monitoring activities between his/her subject file and the eCRF by Roche personnel or Roche contracted monitors and regulatory personnel.

Appropriate forms for subject information and obtaining written informed consent will be provided by the investigator or by Roche/designee.

11.3 Independent Ethics Committees/Institutional Review Board

Independent Ethics Committees (non-US)

This protocol and any accompanying material provided to the subject (such as subject information sheets or descriptions of the study used to obtain informed consent) as well as any advertising or compensation given to the subject, will be submitted by the investigator to an Independent Ethics Committee (IEC). Approval from the committee must be obtained before starting the study, and should be documented in a letter to the investigator specifying the date on which the committee met and granted the approval.

Any modifications made to the protocol after receipt of the IEC approval must also be submitted by the investigator to the Committee in accordance with local procedures and regulatory requirements.

When no local review board exists, the investigator is expected to submit the protocol to a regional committee. If no regional committee exists, Roche will assist the investigator in submitting the protocol to the European Ethics Review Committee.

12. CONDITIONS FOR MODIFYING THE PROTOCOL

Protocol modifications to ongoing studies must be made only after consultation between an appropriate representative of the sponsor and the investigator. Protocol modifications must be prepared by a representative of the sponsor and initially reviewed and approved by the International Medical Leader and Biostatistician.

All protocol modifications must be submitted to the appropriate IEC or Institutional Review Board (IRB) for information and approval in accordance with local requirements, and to Regulatory Agencies if required. Approval must be awaited before any changes can be implemented, except for changes necessary to eliminate an immediate hazard to trial subjects, or when the change(s) involves only logistical or administrative aspects of the trial (e.g. change in monitor(s), change of telephone number(s).

13. CONDITIONS FOR TERMINATING THE STUDY

Both the sponsor and the investigator reserve the right to terminate the study at any time. Should this be necessary, both parties will arrange the procedures on an individual study basis after review and consultation. In terminating the study, Roche and the investigator will assure that adequate consideration is given to the protection of the subject's interests.

14. STUDY DOCUMENTATION, ECRFS AND RECORD KEEPING

14.1 Investigator's Files / Retention of Documents

The Investigator must maintain adequate and accurate records to enable the conduct of this cohort study and the study data to be subsequently verified. These documents should be classified into two different separate categories (1) Investigator's Study File, and (2) subject clinical source documents.

The Investigator's Study File will contain the protocol/amendments, IEC/IRB and governmental approval with correspondence, sample informed consent, staff curriculum vitae and authorization forms and other appropriate documents/correspondence etc.

Subject clinical source documents (usually defined by the project in advance to record key efficacy/safety parameters independent of the eCRFs) would include subject hospital/clinic records, physician's and nurse's notes, appointment book, original laboratory reports, ECG, EEG, X-ray, pathology and special assessment reports, signed informed consent forms, consultant letters, and subject screening and enrollment logs. The Investigator must keep these two categories of documents on file according to local regulations after completion or discontinuation of the study. After that period of time the documents may be destroyed, subject to local regulations.

Should the Investigator wish to assign the study records to another party or move them to another location, Roche must be notified in advance.

If the Investigator can not guarantee this archiving requirement at the investigational site for any or all of the documents, special arrangements must be made between the Investigator and Roche to store these in a sealed container(s) outside of the site so that they can be returned sealed to the Investigator in case of a regulatory audit. Where source documents are required for the continued care of the subject, appropriate copies should be made for storing outside of the site.

14.2 Source Documents and Background Data

The investigator shall supply the sponsor on request with any required background data from the study documentation or clinic records. This is particularly important when errors in data transcription are suspected. In case of special problems and/or governmental queries or requests for audit inspections, it is also necessary to have access to the complete study records, provided that subject confidentiality is protected.

14.3 Audits and Inspections

The investigator should understand that source documents for this trial should be made available to appropriately qualified personnel from the Roche Research Quality Assurance Unit or its designees, or to health authority inspectors after appropriate notification. The verification of the data entered into the eCRF data must be by direct inspection of source documents.

14.4 Electronic Case Report Forms

Data for this study will be entered into an eCRF via a web-based EDC system. For each subject enrolled, an eCRF must be completed by the principal investigator or an authorized delegate from the study staff. This also applies to records for those subjects who fail to complete the study. If a subject withdraws from the study, the reason must be noted on the eCRF.

The investigator should ensure the accuracy, completeness and timeliness of the data reported to the sponsor on the eCRF and in all required reports.

Access to the EDC system will be controlled through the use of unique individual user names and passwords for all entitled study personnel. The investigator will have continual oversight of the electronically reported data of his/her site. Study Clinical Research Associate (CRA) will review study data in the EDC system on an ongoing basis to identify and address data quality issues (e.g. protocol violations) and to ensure the site is continuously entering data into the EDC system. During site monitoring visits, the CRA will verify the data contained in the EDC system against the source documentation and medical records maintained by the investigator.

The system will maintain a full audit trail of entered and changed data along with data on the user.

14.5 Contract Research Organizations

The study protocol was set up in cooperation with Dr. med. [redacted], Dip.Pharm.Med., [redacted] Germany, tel.: [redacted], fax:+[redacted]

15. MONITORING THE STUDY

It is understood that the responsible Roche monitor (or designee) will contact and visit the investigator regularly and will be allowed, on request, to inspect the various records of the trial provided that subject confidentiality is maintained in accord with local requirements.

It will be the monitor's responsibility to inspect the electronic Case Report Forms for all subjects at regular intervals throughout the study, to verify the adherence to the protocol and the completeness, consistency and accuracy of the data being entered on them. The monitor should have access to laboratory test reports and other subject records needed to verify the entries on the eCRF. The investigator (or his/her deputy) agrees to cooperate with the monitor to ensure that any problems detected in the course of these monitoring visits are resolved.

The on-site monitoring may be conducted in 10-15% of subjects in each participating center. The verification of the eCRF data must be by direct inspection of source documents for the selected subjects.

16. CONFIDENTIALITY OF TRIAL DOCUMENTS AND SUBJECT RECORDS

The investigator must assure that subjects' anonymity will be maintained and that their identities are protected from unauthorized parties. On eCRFs or other documents submitted to the sponsor, subjects should not be identified by their names, but by an identification code. The investigator should keep a subject enrollment log showing codes, names and addresses. The investigator should maintain documents not for submission to Roche, e.g., subjects' written consent forms, in strict confidence.

17. PUBLICATION OF DATA AND PROTECTION OF TRADE SECRETS

The results of this study may be published or presented at scientific meetings. If this is foreseen, the investigator agrees to submit all manuscripts or abstracts to Roche prior to submission. This allows the sponsor to protect proprietary information and to provide comments based on information from other studies that may not yet be available to the investigator.

In accord with standard editorial and ethical practice, Roche will generally support publication of multicenter trials only in their entirety and not as individual center data. In this case, a coordinating investigator will be designated by mutual agreement.

Any formal publication of the study in which input of Roche personnel exceeded that of conventional monitoring will be considered as a joint publication by the investigator and the appropriate Roche personnel. Authorship will be determined by mutual agreement.
